# Mechanistic Insights into the Direct Partial Oxidation of Methane to Methanol Catalyzed by Single-Atom Transition Metals on Hydroxyapatite

**DOI:** 10.1021/acsomega.4c09442

**Published:** 2025-01-22

**Authors:** Albert F. B. Bittencourt, Pedro Ivo R. Moraes, Juarez L. F. Da Silva

**Affiliations:** † Institute of Science and Technology, Federal University of Jequitinhonha and Mucuri Valleys, Diamantina, Minas Gerais 39100-000, Brazil; ‡ São Carlos Institute of Chemistry, University of São Paulo, P.O. Box 780, São Carlos, São Paulo 13560-970, Brazil

## Abstract

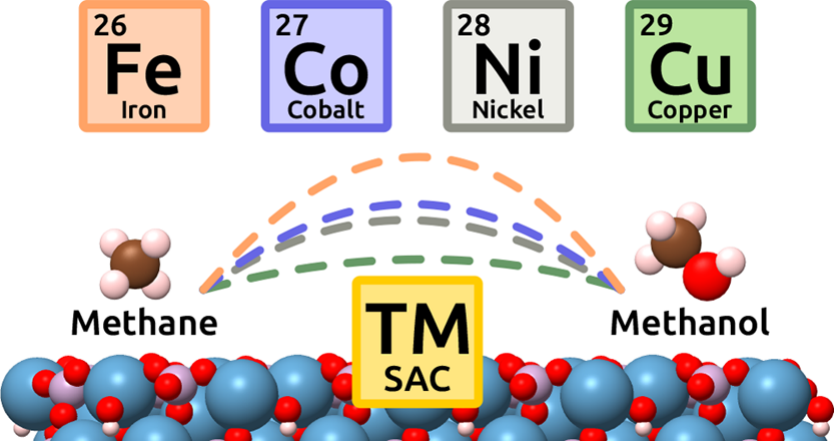

The direct conversion of methane to methanol offers a promising approach to utilize abundant natural gas resources; however, the finding of suitable low-cost catalysts remains challenging due to the chemical inertness of methane. In this study, we performed a theoretical investigation of the role of transition-metal single-atom catalysts (TM-SACs) anchored on the hydroxyapatite support, where TM = Fe, Co, Ni, and Cu. We examined adsorption properties, formation of oxidized active sites, methane activation, methanol formation, and its stability using density functional theory calculations with van der Waals corrections, combined with the climbing image nudged elastic band method for the localization of transition states. Our findings reveal that Cu/HAP exhibits the most favorable energy profile for the conversion of methane to methanol, offering lower activation barriers and a more exothermic reaction pathway compared to other systems. In contrast, Fe/HAP shows superior oxygen dissociation capabilities but faces challenges in methanol production due to higher reaction barriers. These findings provide valuable information for the future design of TM/HAP catalysts for sustainable methane utilization.

## 1 Introduction

A significant challenge in utilizing the abundance of natural gas feedstocks remains in the inherent difficulty of converting methane (CH_4_) into chemicals of higher value.^1,2^ For methanol (CH_3_OH) production, current industrial processes typically involve the synthesis of syngas by reforming, followed by its conversion under harsh conditions, increasing operational costs.^3,4^ In response to the urgent transition to sustainable energy, increasing interest has been placed in the development of alternative reaction routes. In particular, direct conversion of CH_4_ to CH_3_OH offers a potential solution; however, significant challenges hinder its large-scale implementation.^5^ The stability of CH_4_ and the need for continuous processes require highly active and selective catalysts that can efficiently convert CH_4_ while avoiding catalyst poisoning or overoxidation of CH_3_OH.^4,6,7^

Heterogeneous catalysts with supported nanostructures are commonly applied in direct methane-to-methanol conversion processes. The effectiveness of these catalysts is highly dependent on the size of the metal particles.^8^ Heterogeneous catalysts with atomically dispersed metal atoms, also called SACs, were first described by Qiao et al.,^9^ who reported high carbon monoxide (CO) oxidation activity using a Pt_1_/FeO_*x*_ catalyst. SACs have demonstrated potential in enhancing selectivity,^10^ metal-utilization efficiency,^11^ optimizing the interaction between metal and support,^12^ and occasionally even enhancing stability.^13^ Since Guo et al.^14^ used single iron sites implanted in a silica matrix for the direct nonoxidative conversion of CH_4_ into ethylene (C_2_H_4_), benzene (C_6_H_6_), and naphthalene (C_10_H_8_), the use of SACs in CH_4_ conversion has attracted a lot of attention.

In light of these advantages, calcium hydroxyapatite (HAP, Ca_10_(PO_4_)_6_(OH)_2_) has been identified as an effective support for metallic SACs. HAP-based materials exhibit unique bifunctional properties with acidic and basic properties available within their structure^15,16^ As reported in the pioneering work of Sugiyama et al.,^17^ this property can be advantageous in the oxidative activation of CH_4_, allowing adaptation of conversion and selectivity by modifying the acidic-basic surface distribution. In addition, HAP can form synergistic interactions with a variety of TM,^18−22^ thus revealing promising behavior as a support for TM single-atom and nanoparticle catalysts active in CH_4_ oxidation processes.

Previous studies have shown that HAP-based catalysts are also resistant to coke formation. Based on density functional theory (DFT) calculations, Akri et al. demonstrated that Ni-SACs supported on HAP exhibit enhanced stability and reduced coke formation during methane oxidation reactions, making these catalysts highly effective for prolonged use.^23^ Recent experimental and theoretical findings revealed that HAP basic sites were responsible for inhibiting coke formation on the catalyst, while the distribution of HAP surface sites assists in the stabilization of anchored Ni particles.^24^ Resistance to coke formation has also been reported for other TM/HAP-supported catalysts, such as Rh/HAP,^20^ Pd/HAP,^21^ and Co/HAP.^25^ Despite numerous studies on HAP support, to the best of our knowledge, the catalytic properties of TM/HAP SACs in the direct methane-to-methanol reaction have not yet been investigated.

Therefore, the present study aims to carry out a comparative study using DFT calculations to evaluate the physicochemical properties of four different TM-SACs (TM = Fe, Co, Ni, Cu) supported on the HAP(0001) surface, forming the TM/HAP systems for potential application in direct conversion of methane to methanol. These TM were selected for their distinct affinities for the adsorption and activation of small molecules, as well as their cost-effectiveness compared to noble metals, making them attractive for practical catalytic applications. In addition, the catalytic activity of these TM species has been widely reported in the literature, particularly in methane conversion reactions, further motivating their selection for this investigation.^4,5,26−28^ We first analyze the adsorption of TM, followed by its oxidation. Then, we investigate the sequence of elementary reactions involved in the conversion of methane to methanol and assess the stability of methanol on the catalyst surface. Activation energy barriers were estimated using the climbing image nudged elastic band (CI-NEB) method. In general, our results demonstrate that the formation of active sites and their reactivity in the conversion of methane to methanol are strongly influenced by the specific nature of TM-SAC.

## 2 Theoretical Approach and Computational Details

### 2.1 Total Energy Calculations

All total energy calculations were performed within the spin-polarized DFT framework,^29,30^ employing the Perdew–Burke–Ernzerhof (PBE) formulation of the semilocal generalized gradient approximation (GGA) for the exchange-correlation energy functional.^31^ To enhance the description of the long-range van der Waals (vdW) interactions, the Grimme D3 semiempirical correction was used for all calculations.^32^ The frozen-core projector augmented wave (PAW) method^33,34^ was used to model the interactions between core and valence electrons, while the Kohn–Sham states were expanded in plane-wave. All calculations were performed using the Vienna Ab initio Simulation Package (VASP), version 5.4.4.^35,36^

To ensure the precision required to describe chemical reactions on solid surfaces, all calculations used a plane-wave cutoff energy of 489 eV, which exceeds the maximum recommended plane-wave cutoff energy by 12.5% considering all selected PAW projectors. For the integration of the Brillouin zone (BZ), a 2 × 2 × 1 Monkhorst–Pack^37^**k**-point mesh was employed for structure optimizations and a 4 × 4 × 1 **k**-point mesh for calculations of the density of states. For gas-phase molecules and isolated atoms, only the Γ-point was considered due to the lack of dispersion in the electronic states within the BZ. The equilibrium configurations were obtained once the atomic forces were smaller than 0.025 eV Å^–1^ on each atom, using a total energy convergence criterion of 1 × 10^–5^ eV.

### 2.2 Atomic Structure Configurations

The modeling of the HAP support was based on previous studies conducted within our research group, where we investigated the effect of the bifunctional properties of HAP on the adsorption of probe molecules^16^ and the catalytic valorization of ethanol for biofuel production.^38^ The slab model was constructed considering the hexagonal bulk structure of HAP,^39^ with the stoichiometric HAP(0001) termination selected to support the investigated TM SAC. This surface termination contains two types of adsorption sites: positively charged Ca^2+^ ions and negatively charged PO_4_^3–^ groups, which is advantageous for modeling the bifunctional properties found in materials based on HAP. For simplicity, we will write only HAP when referencing the HAP(0001) surface.

To accurately represent the surface properties, a 1 × 1 surface unit cell was built with dimensions of *a*_0_ = *b*_0_ = 9.497 Å, consisting of four formula units (one formula unit, Ca_5_(PO_4_)_3_(OH), per layer), thickness of 12.88 Å, and a vacuum region of 15 Å. After fully optimizing the atomic positions of the clean surface slab, the bottom atomic layer was kept frozen. For the adsorption of the TM-SACs, a set of 15 unique distinct structures were designed for each adsorption system as initial configurations for the search for the lowest energy adsorption structures.

Due to the large number of calculations, a screening process was performed using smaller computational parameters (cutoff energy of 380 eV with Γ-point only, total energy and force convergence criteria of 1 × 10^–4^ eV and 0.050 eV Å^–1^, respectively). Then, after identifying the lowest energy structures, each adsorption system was reoptimized using the standard computational parameters aforementioned, i.e., cutoff energy of 489 eV with 2 × 2 × 1 **k**-point mesh, total energy and force convergence criteria of 1 × 10^–5^ eV and 0.025 eV Å^–1^, respectively.

The lowest energy adsorption configuration for each TM-SAC was selected as the substrate in which the reaction will take place. The molecules and molecular fragments were then initially positioned at a distance of about 2 Å, generating a set of 15 structures for each adsorption system. After completing atomic optimization with smaller computational parameters for the entire set, the lowest energy configurations were identified and reoptimized using standard computational parameters defined above. It should be noted that all adsorbates were placed on the top side of the HAP(0001) surface while keeping the bottom atomic layer frozen. This approach could lead to a net dipole moment across the slab. To access this effect on the adsorption properties of our systems, an evaluation of the slab model parameters is presented in the Supporting Information file.

Fully optimization was carried out for all isolated gas-phase molecules and molecular fragments using a 20 Å cubic box. The initial geometries were obtained from the NIST Computational Chemistry Comparison and Benchmark Database.^40^ For isolated atoms, an orthorhombic box of 20 × 21 × 22 Å was used to break the electron density symmetry, which is essential to prevent spherical electron densities and fractional occupation of electronic states.

### 2.3 Proposed Reactions

This study aims to achieve two primary objectives: (i) characterization of the direct conversion of methane to methanol by partial oxidation, and (ii) evaluation of the methanol stability on TM/HAP substrate. To this end, the following reaction sequence was proposed:
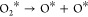
R1
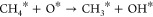
R2
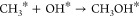
R3
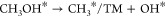
R4
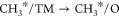
R5

**Figure 1:**
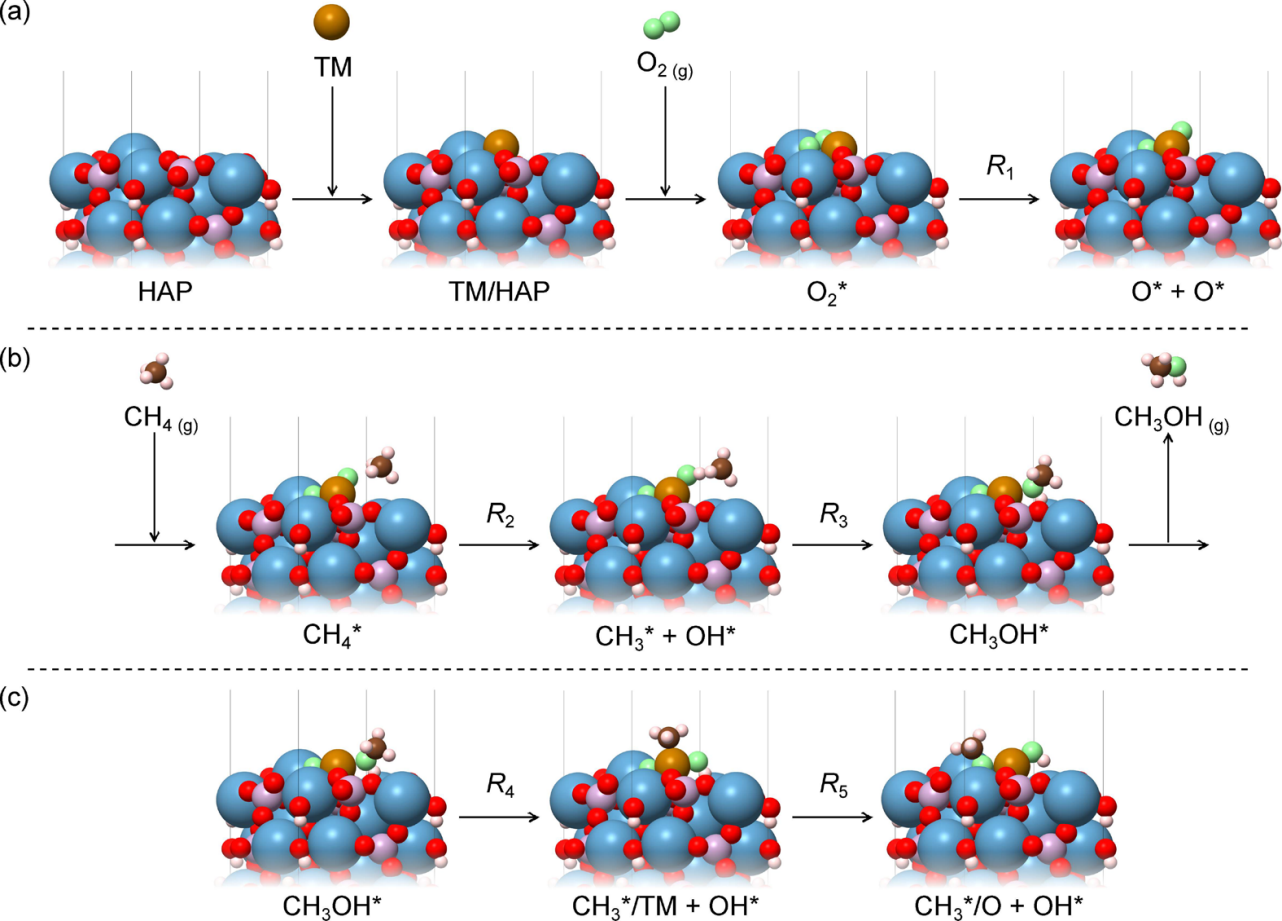
Schematic representation of the elementary reactions proposed for investigating the direct methane conversion to methanol on TM/HAP catalysts. (a) Adsorption of TM and O_2_ followed by dissociation of the O_2_* molecule (reaction *R*_1_). (b) Adsorption of methane followed by its activation (reaction *R*_2_), methanol formation (reaction *R*_3_) and methanol desorption. (c) Methanol decomposition (reaction *R*_4_) and CH_3_* diffusion to a neighboring oxygen site (reaction *R*_5_).

The schematic representation of the elementary reactions is shown in Figure 1. The catalytic surface for each reaction system was constructed by adsorbing one TM-SAC on the HAP clean surface. Subsequently, the TM-SAC was then oxidized by O_2_. The adsorption of this molecular oxidant is crucial in the formation of O/TM active sites, obtained via oxygen dissociation (reaction *R*_1_). Then, the direct conversion of methane to methanol proceeds through a homolytic pathway, in which one of the C–H bonds within the CH_4_ molecule is cleaved through a transition state (reaction *R*_2_). This step leads to the formation of an intermediate composed of an OH moiety coordinated to the TM-SAC and an uncoordinated CH_3_ radical, which recombines directly with the coordinated OH moiety to form the methanol molecule (reaction *R*_3_). This reaction pathway has been recently reported for other SACs.^28,41,42^

Given the concern of catalyst deactivation due to the formation of carbonaceous species,^43,44^ we investigated the stability of methanol by examining its dissociation into CH_3_ and OH. This dissociation proceeds through a transition state, leading to both species bonded to the TM-SAC (reaction *R*_4_). Subsequently, CH_3_ undergoes diffusion to an adjacent oxygen site (reaction *R*_5_), yielding a more thermodynamically favorable adsorption configuration.

### 2.4 Localization of Transition States

The transition state (TS) structures were located using the CI-NEB method, which ensures that the climbing image reaches the exact saddle point upon convergence.^45,46^ For each elementary reaction, a set of 12 images was created by performing a linear interpolation between the optimized structure of the initial and final adsorption systems. The reaction path was then determined by simultaneously optimizing all 12 images using the quick-min force-based optimizer^47,48^ with a force convergence criteria of 0.025 eV Å^–1^. Subsequently, the TS structures were confirmed by identifying a single imaginary mode. A comprehensive description of the converged reaction paths and TS structures is provided in the Supporting Information file.

## 3 Results and Discussion

### 3.1 Transition Metal Single-Atom Catalysts

The TM/HAP-supported catalysts exhibited a preference for anchoring the TM on top of surface-exposed oxygen atoms, with the shortest TM–O bond distances of 2.03, 1.84, 1.84, and 1.97 Å for Fe, Co, Ni, and Cu, respectively. To further characterize the coordination environments, the effective coordination numbers were evaluated using the Critic2 program.^49,50^ The results showed effective coordination numbers of 1.8, 2.0, 2.0, and 1.0 for Fe, Co, Ni and Cu, respectively. As recently reported,^51^ the PO_4_^3–^ groups within the HAP structure are negatively charged, resulting in a Lewis basic character. Consequently, these species can act as charge donors, which aligns with the atomic net charges found for TM-SAC, exhibiting values of −0.10, −0.16, −0.17, and −0.13 *e* for Fe, Co, Ni, and Cu, respectively. The analysis of the local density of states also supports this observation, showing that the Lewis basic character associated with the valence band is largely dominated by the oxygen *p*-states within PO_4_^3–^ groups. Furthermore, anchoring of the TM-SAC creates localized states near the Fermi level, resulting in distinct active sites on the catalyst surface.

**Figure 2:**
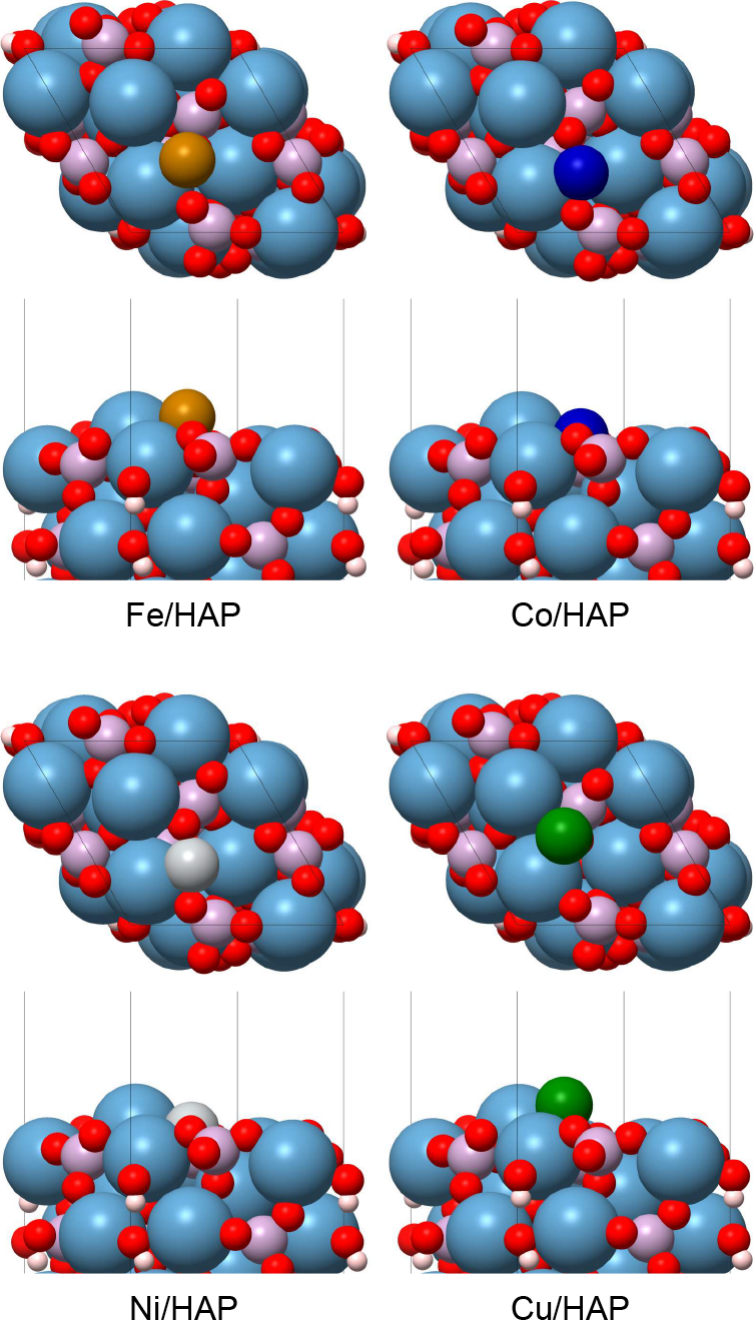
Top and side views of the optimized adsorption configurations for the TM-SACs on the HAP(0001) surface. Light blue, pink, red, and white spheres represent Ca, P, O, and H atoms in the substrate, while ocher, dark blue, silver, and dark green spheres represent Fe, Co, Ni, and Cu SACs, respectively.

The stability of TM-SACs was evaluated on the basis of the adsorption energy criteria. For the lowest-energy configurations, shown in Figure 2, the adsorption energy values were significantly negative, with −1.48, −1.47, −2.19, and −1.13 eV for Fe, Co, Ni and Cu, respectively. This finding indicates that TM-SACs are tightly bound to the HAP Lewis basic sites, which could be advantageous to stabilize the anchored atomic species. Among the studied metals, Ni exhibited the strongest metal–support interaction, as evidenced by its most negative adsorption energy. However, previous research by Akri et al.^23^ suggests that Ni/HAP SACs are susceptible to sintering at high temperatures. To address this limitation, doping the HAP support with cerium has been identified as a promising solution.

### 3.2 Oxygen Adsorption and Dissociation

**Figure 3:**
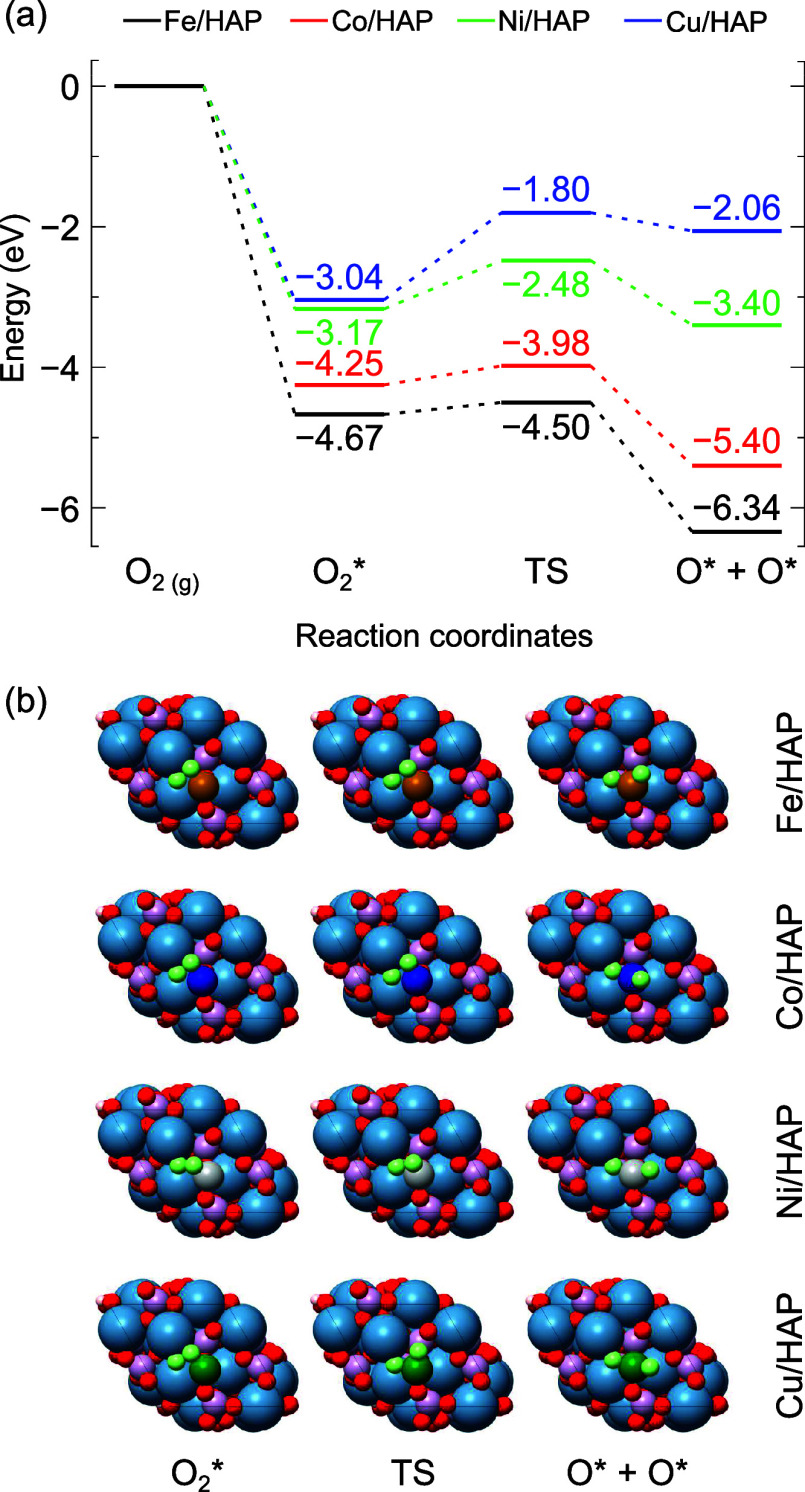
(a) Reaction energy profile for the oxygen dissociation reaction on TM/HAP catalysts. (b) Top views of the adsorbed oxygen molecule (O_2_*), the transition state structure TS, and the dissociated oxygen fragments (O* + O*). All energies are relative to O_2_ in the gas phase and are presented in eV.

Oxygen was selected as the oxidant for the direct conversion of methane to methanol. This conversion process begins with the adsorption of O_2_ onto TM/HAP substrates, followed by its dissociation to form the O/TM active sites. Multiple adsorption configurations of O_2_ on the TM-SACs were explored (Figures S6–S9), with the most stable structures selected for detailed analysis. As depicted in Figure 3, the O_2_ molecule is preferentially adsorbed between the most exposed Ca^2+^ ion and the TM-SAC. All TM-SACs exhibited favorable O_2_ adsorption, with energies values of −4.67, −4.25, −3.17, and −3.04 eV for Fe, Co, Ni, and Cu, respectively. This interaction between adsorbed O_2_ molecule and the substrate resulted in elongation of the O–O bond, which followed a decreasing trend of 0.29, 0.28, 0.21, and 0.22 Å from Fe to Cu, suggesting that the degree of activation of O_2_ decreases from Fe to Cu.

The activation energies for O_2_ dissociation (*R*_1_, Table 1) increased in the order of Fe, Co, Ni, and Cu, with reaction barriers of 0.17, 0.27, 0.69, and 1.24 eV, respectively. This trend is consistent with the findings of Arachchige et al.,^28^ who reported activation energies of 0.13, 0.66, 2.17, and 2.56 eV for Fe, Co, Ni, and Cu TM-SACs supported on graphyne, respectively. Although the activation energies reported for Ni and Cu were significantly higher than those observed in our study, both studies demonstrate a decrease in the activation efficiency O_2_ from Fe to Cu.

**Table 1:** Activation Energies for Each Elementary Reaction on TM/HAP Systems^a^

catalyst	*R*_1_	*R*_2_	*R*_3_	*R*_4_	*R*_5_
Fe/HAP	0.17	1.44	0.23	1.73	1.18
Co/HAP	0.27	0.83	0.05	1.65	0.95
Ni/HAP	0.69	0.70	0.08	2.15	0.71
Cu/HAP	1.24	0.31	0.06	2.28	0.35

a All values are presented in eV.

Interestingly, the dissociation reactions were exothermic for Fe, Co, and Ni (−1.67, −1.15, and −0.23 eV, respectively), while Cu exhibited an endothermic dissociation process with an energy of 0.98 eV (see energy profiles in Figure 3). Due to the higher O_2_ activation energy and the endothermic nature of the dissociation, the Cu/HAP substrate faces significant limitations in initiating the partial oxidation of methane compared to the other substrates.^52,53^ At the transition state, the O–O bond length was measured as 1.74, 1.81, 1.90, and 2.17 Å for Fe, Co, Ni, and Cu, respectively.

For comparison, we calculated the dissociation O_2_ on the clean HAP(0001) support. The barrier for this process is 4.44 eV, which highlights the crucial role of TM-SACs to form active sites on the HAP support. Given this high barrier on the pristine substrate, subsequent steps for the conversion of methane were not addressed on the clean HAP support. After O_2_ dissociation, the electronic structure of the substrates changes notably. The TM-SACs acquire positive effective charges that decrease from Fe to Cu. The dissociated oxygen radicals exhibit negative charges, though less negative than structural oxygen. Importantly, the average charges of Ca, P, O, and H in the HAP surface remain unchanged (Table S4). In addition, near the Fermi level, the *p*-states of dissociated oxygen hybridize with the transition metal *d*-states, which enables the metallic single-atom site to generate active oxygen intermediates for methane activation.^54^

### 3.3 Methane Conversion into Methanol

Methane was absorbed onto O/TM active sites through a C–H···O interaction, with bond distances of 2.54, 2.72, 3.12, and 2.73 Å for Fe, Co, Ni, and Cu, respectively. In all cases, the adsorption resulted in only a slight distortion of the tetrahedral geometry of methane. In addition, the low adsorption energy values (−0.17, −0.22, −0.20, and −0.31 eV for Fe, Co, Ni, and Cu, respectively) indicate weak physisorption across all catalysts.

**Figure 4:**
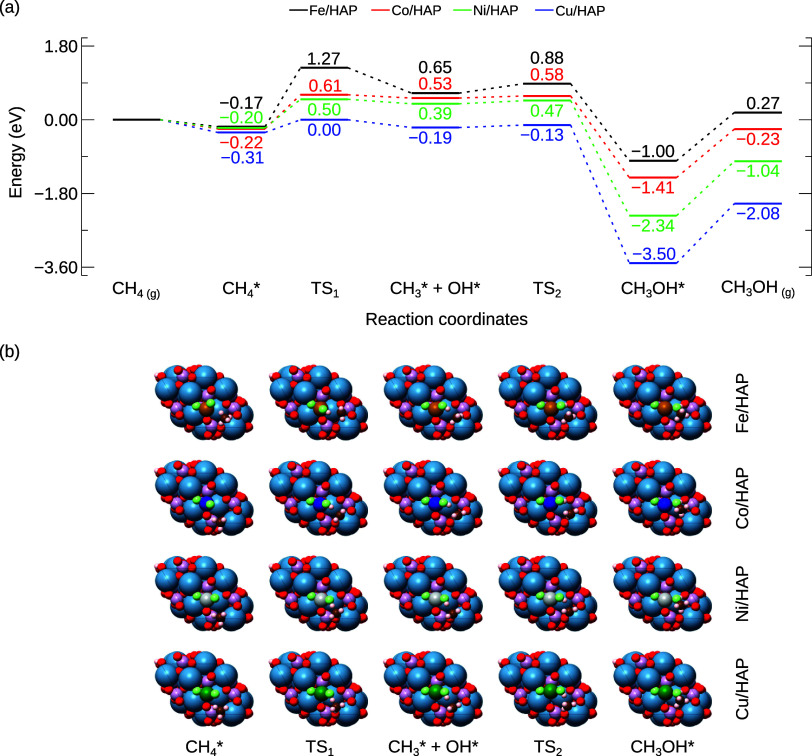
(a) Reaction energy profile for the conversion of methane to methanol on TM/HAP catalysts. (b) Top views of the adsorbed methane molecule (CH_4_*), the transition state structure (TS_1_), the intermediate state (CH_3_* + OH*), the transition state structure (TS_2_), and the formed methanol molecule (CH_3_OH*). All energies are relative to CH_4_ in the gas phase and are presented in eV.

As depicted in Figure 4, the conversion of methane to methanol proceeds through the cleavage of a C–H bond, generating a methyl radical directed toward a hydroxyl coordinated with the TM-SAC. In many cases, the rate-determining step in methane conversion is considered to be the cleavage of the C–H bond in the CH_4_ molecule.^54−56^ This behavior is also observed during the conversion of methane to methanol on TM/HAP substrates. The activation energy barriers decrease from Fe to Cu (*R*_2_, Table 1). Interestingly, the substrate with the strongest interaction with methane (Cu/HAP with −0.31 eV) exhibited the lowest reaction barrier (0.31 eV), while the substrate with the weakest interaction (Fe/HAP with −0.17 eV) resulted in the highest reaction barrier (1.44 eV). In the transition state TS_1_, the methyl radical is formed with a distance CH_3_···HO/TM measuring 2.14, 2.02, 1.43, and 1.32 Å for the Fe, Co, Ni, and Cu substrates, respectively.

To further elucidate the catalytic behavior of TM/HAP systems, we compared our results with previous studies on other catalytic systems. In the work of Mahyuddin et al., the direct conversion of methane to methanol was investigated using TM-exchanged ZSM-5 zeolite with TM = Fe, Co, Ni, Cu.^27^ Their findings indicate that the reactivity toward C–H bond cleavage of methane increases in the order of Co-ZSM-5 (0.75 eV), Ni-ZSM-5 (0.69 eV), Fe-ZSM-5 (0.56 eV), and Cu-ZSM-5 (0.28 eV). These calculated barriers are relatively similar to values obtained for our HAP-supported systems, with the exception of Fe-ZSM-5, which exhibits a lower barrier. Exploring another class of materials of interest for the conversion of methane to methanol, Arachchige et al. reported values of 1.21 and 0.82 eV for graphyne-modified SACs with Fe and Co, respectively.^28^ For comparison, the activation energy barrier for methane C–H bond cleavage on pure metallic surfaces, such as Co(111), Ni(111), and Cu(111), have been estimated as 1.02, 0.89, and 1.64 eV, respectively.^57^

Following the C–H bond cleavage, the CH_3_ fragment interacts with OH moiety coordinated to the TM, adopting an sp^2^ hybridized geometry. This state represents a reaction intermediate, acting as a local minimum between the initial state (adsorbed methane) and the final state (adsorbed methanol). The energy of this intermediate state is higher than that of both the initial and final states. The next step in the formation of methanol involves the coupling of the CH_3_ and OH species. The low reaction barriers observed for TS_2_ correspond to the reorientation of the hydroxyl bonded to the TM and the subsequent bond formation with the methyl radical (*R*_3_, Table 1). Our findings indicate that the Fe/HAP substrate exhibited the highest barrier for this step at 0.23 eV, while Co, Ni, and Cu substrates showed similar barriers of 0.05, 0.08, and 0.06 eV, respectively. Lastly, it should be mentioned that the partial oxidation of methane to methanol is energetically favored across all TM/HAP catalysts, with Cu/HAP being the most favorable substrate for this reaction.

### 3.4 Methanol Stability

**Figure 5:**
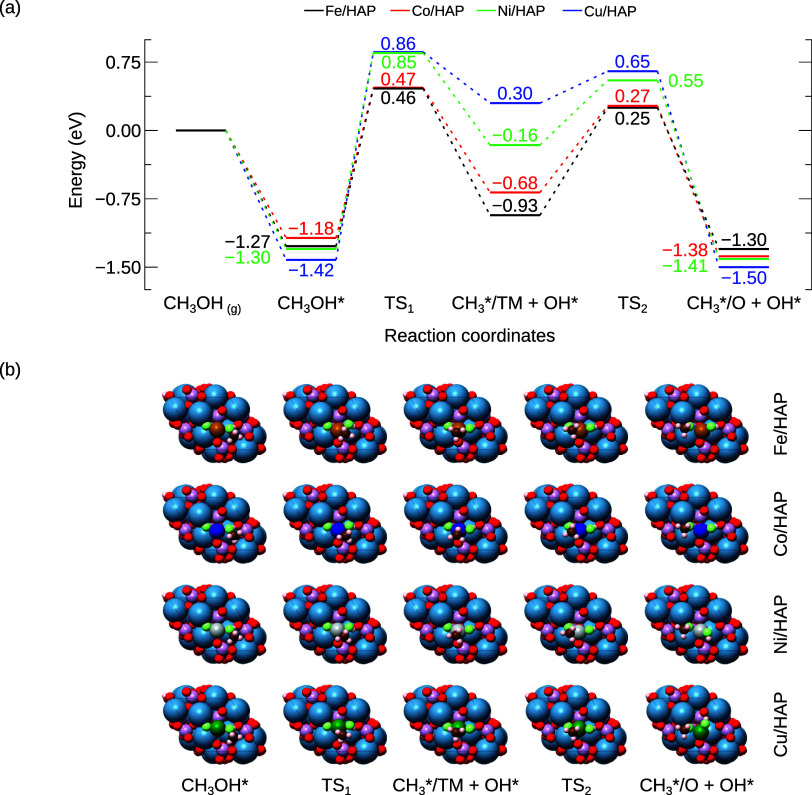
(a) Reaction energy profile for the methanol decomposition reaction and the diffusion of the CH_3_* on TM/HAP catalysts. (b) Top views of the adsorbed methanol molecule (CH_3_OH*), the transition state structure (TS_1_), the dissociated fragments (CH_3_*/TM + OH*), the transition state structure (TS_2_), and the CH_3_* fragment adsorbed on a neighboring oxygen site (CH_3_*/O + OH*). All energies are relative to CH_3_OH in the gas phase and are presented in eV.

The formed methanol is adsorbed onto the substrate via hydroxyl interactions with the TM-SAC. As illustrated in Figure 5, the negative values of the adsorption energies indicate that the methanol adsorption is energetically favorable across all substrates. To assess its stability on TM/HAP surfaces, we evaluated the dissociation of methanol through cleavage of the C–OH bond, resulting in the formation of fragments CH_3_ and OH, both coordinated with the active site TM. This step is followed by the diffusion of the CH_3_ radical to a slightly more stable configuration.

The cleavage of the methanol C–OH bond (*R*_4_, Table 1) exhibits the highest energy barrier among all the reactions evaluated. Cu/HAP shows the highest barrier at 2.28 eV, followed by Ni, Fe, and Co with energy barriers of 2.15, 1.73, and 1.65 eV, respectively. In the transition state, C–OH bond lengths increase to 1.88, 1.91, 1.96, and 2.17 Å, compared to the predissociation lengths of 1.45, 1.44, 1.44, and 1.45 Å for the Fe, Co, Ni, and Cu substrates, respectively.

In the subsequent step (*R*_5_, Table 1), the diffusion barrier of the CH_3_ fragment decreases progressively from Fe to Cu, with values of 1.18, 0.95, 0.71, and 0.35 eV for Fe, Co, Ni, and Cu, respectively. Although diffusion of the CH_3_ fragment leads to more energetically favorable structures, the cleavage of the C–OH bond remains endothermic, requiring more energy than the cleavage of the methane C–H bond. This finding suggests that TM-SACs supported on HAP substrates may enable stable methane-to-methanol conversion, from an energetic perspective.

## 4 Insights into the Assessment of Activation Energy Barriers: NEB versus UBI-QEP

The CI-NEB method has been consistently used to elucidate reaction pathways, particularly to identify transition states and calculate activation energy barriers.^58^ However, this approach is computationally demanding due to the large number of intermediate images required to accurately interpolate between initial and final reaction states. As an alternative to reduce computational cost, the unity bond index-quadratic exponential potential (UBI-QEP) method has been employed in studies of reactions on solid surfaces.^59^ This method simplifies the calculation of activation energies by relying on adsorption energies and gas-phase bond dissociation energies. In its standard formulation, the activation energy is given by
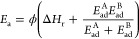
1where Δ*H*_r_ is the enthalpy of the surface reaction, which includes the binding energy of molecule AB, the energy needed to dissociate gas-phase AB into fragments A and B, and the binding energies of these fragments, defined as .

Building on this model, Maestri and Reuter introduced a modified parametrization to improve the accuracy of activation energy barriers.^60^ Their revised formulation for the activation energy is given by the following equation,
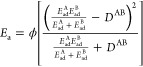
2This framework removes the direct dependence on the binding energy of AB, which can sometimes introduce spurious contributions.^60^ In both equations, the interpolation parameter ϕ accounts for the nature of the TS and typically ranges from 0 to 1. As Maestri and Reuter pointed out, the empirical choice of ϕ = 0.5 in the standard UBI-QEP model does not capture any specific characteristics of the TS. To address this, they recommend adjusting ϕ to align the UBI-QEP barrier predictions more closely with ab initio references, enabling more accurate modeling of complex reaction mechanisms.^60^

To evaluate the performance of the UBI-QEP method in predicting activation energies relative to those calculated using the CI-NEB method, we initially applied the standard empirical value of 0.5 for the interpolation parameter ϕ. Subsequently, we adjusted this parameter to optimize the correlation between CI-NEB results and UBI-QEP predictions obtained using both frameworks. Our analysis included the calculated activation barriers for the dehydrogenation of methane, the formation of methanol, and the reactions of bond cleavage of methanol C–OH.

**Figure 6:**
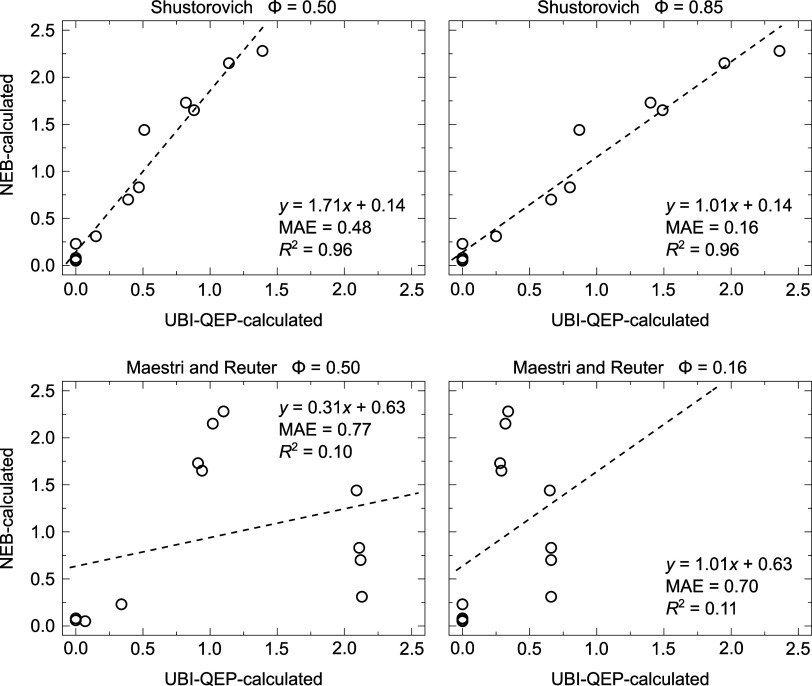
Correlation for activation energies calculated using CI-NEB and UBI-QEP methodologies.

Figure 6 illustrates the correlation between the activation energies calculated using the CI-NEB and UBI-QEP methods. The standard Shustorovich approach, with the empirical interpolation parameter ϕ = 0.50, shows a strong linear correlation, achieving a *R*^2^ value of 0.96. However, the slope (1.71) and the mean absolute error (MAE = 0.48) suggest that UBI-QEP tends to overestimate the activation barriers relative to those obtained using CI-NEB. Upon adjusting the ϕ parameter to 0.85, the correlation improves significantly. The slope (1.01) is closely aligned with unity, indicating better agreement between the UBI-QEP and CI-NEB methods. The MAE also decreases to 0.16, further demonstrating that this adjustment more accurately captures the nature of the TS in the reactions evaluated. In contrast, the Maestri and Reuter approach, with empirical ϕ = 0.50, shows a much weaker correlation, as reflected by a lower *R*^2^ value of 0.10 and a higher MAE of 0.77. Even after adjusting ϕ to 0.16, the correlation remains poor, with a *R*^2^ of 0.11 and a MAE of 0.70, offering limited improvement compared to the standard Shustorovich approach.

Given these findings, we believe that the poorer performance of the Maestri and Reuter modified parametrization can be attributed to the empirical refinement that eliminates the direct dependence on the binding energy of the undissociated species. Although this refinement can be effective in specific cases, it does not always capture the variability in the transition state characteristics across different reaction systems. Consequently, the modified method may suffer a loss of generality. The refinements introduced by Maestri and Reuter, while intended to improve accuracy, can lead to overfitting for specific conditions and a loss of general applicability compared to the simpler and more robust Shustorovich method.

## 5 Conclusions

This study provides a comprehensive analysis of TM-SACs supported on the HAP surface for the conversion of methane to methanol. By evaluating the interaction between the TM atoms and the substrate, it was found that the TM/HAP-supported catalysts exhibit strong adsorption energies, indicating their stability under reaction conditions.

For the formation of active sites via oxygen dissociation, Fe/HAP demonstrated the lowest energy barrier. The activation energies value increased consistently from Fe to Cu, highlighting that the tuning of the catalytic properties depends on the transition metal used. In contrast, in the conversion of methane to methanol, a decreasing trend was observed in the activation energy barriers from Fe to Cu for the cleavage of the C–H bond determining the rate. For this reaction step, Cu/HAP was determined as the catalyst with the lowest activation energy barrier and the most favorable energy profile. Furthermore, the evaluation of methanol stability through C–OH bond cleavage revealed that this reaction step has the highest energy barrier among all the reactions evaluated, suggesting stable methanol formation in the TM/HAP catalysts.

The assessment of the selected activation energy barriers using the UBI-QEP method demonstrated its potential as a cost-effective alternative to CI-NEB. Adjusting the interpolation parameter ϕ, an improvement in the correlation between the UBI-QEP and CI-NEB results can be obtained, leading to more accurate predictions of activation energies, especially for the Shustorovich approach which provided a more reliable correlation with the NEB-calculated activation energies for our systems. These findings are expected to be valuable in the development of new TM/HAP catalysts, which contribute to the direct conversion of methane to methanol.

## Data Availability

All DFT calculations were conducted using the VASP software (version 5.4.4), which is available under a nonfree academic license. For more information, please visit the VASP Web site at https://www.vasp.at/. Additional details are available in the Supporting Information, and additional raw data can be obtained from the authors upon request.

## Supporting Information Available

Additional technical details and complementary analyzes on the screening set of adsorption structures, electronic and adsorption properties of TM-SACs, activation energy barriers, reaction paths, and confirmation of TS structures (PDF)
O_2_ dissociation (MP4)
CH_3_OH dissociation (MP4)
CH_3_OH formation (MP4)
