# Supplementary material for: Mechanistic Insights into the Direct Partial Oxidation of Methane to Methanol Catalyzed by Single-Atom Transition Metals on Hydroxyapatite
Source: ACS Omega. 2025 Jan 22;10(4):3868–77. doi: 10.1021/acsomega.4c09442 (PMC11800041; doi:10.1021/acsomega.4c09442)
Supplement: Supplementary file 1 — ao4c09442_si_001.pdf [file ao4c09442_si_001.pdf]

Supporting Information: Mechanistic Insights into the Direct Partial Oxidation of Methane to Methanol Catalyzed by Single-Atom Transition Metals on Hydroxyapatite

Albert F. B. Bittencourt,[*,†,‡] Pedro Ivo R. Moraes,[*,‡] and Juarez L. F. Da Silva[*,‡]

†*Institute of Science and Technology, Federal University of Jequitinhonha and Mucuri Valleys,* 39100-000, *Diamantina, MG, Brazil*

‡*São Carlos Institute of Chemistry, University of São Paulo, P.O. Box* 780, 13560-970, *São Carlos, SP, Brazil*

E-mail: albertbittenc@ict.ufvjm.edu.br; pedroivormm@gmail.com; juarez_dasilva@iqsc.usp.br

Contents

S-1 Introduction **S-4**

S-2 Additional PAW Projector Details **S-4**

S-3 Additional Computational Tests on Selection of Slab Models **S-5**

S-4 Complete Set of Optimized Adsorption Structures **S-7**

S-4.1 Optimized Configurations for TM-SACs on HAP S-7

S-4.2 Optimized Configurations for O_2 on TM-SACs S-11

S-4.3 Optimized Configurations for Dissociated O_2 on TM-SACs S-15

S-4.4 Optimized Configurations for CH_4 on TM-SACs S-19

S-4.5 Optimized Configurations for Methanol on TM-SACs S-23

S-4.6 Optimized Configurations for Dissociated Methanol on TM-SACs . . S-27

S-5 Electronic Properties of HAP-based Systems **S-30**

S-6 Adsorption Properties of HAP-based Systems **S-32**

S-7 Activation Energy Barriers **S-33**

S-7.1 Reaction Path for Oxygen Dissociation S-35

S-7.1.1 $O_2^* \xrightarrow[\text{Fe/HAP}]{\text{TS}} O^* + O^*$ S-35

S-7.1.2 $O_2^* \xrightarrow[\text{Co/HAP}]{\text{TS}} O^* + O^*$ S-36

S-7.1.3 $O_2^* \xrightarrow[\text{Ni/HAP}]{\text{TS}} O^* + O^*$ S-37

S-7.1.4 $O_2^* \xrightarrow[\text{Cu/HAP}]{\text{TS}} O^* + O^*$ S-38

S-7.2 Reaction Path for C – H Bond Cleavage S-39

S-7.2.1 $CH_4^* + O^* \xrightarrow[\text{Fe/HAP}]{\text{TS}_1} CH_3^* + OH^*$ S-39

S-7.2.2 $CH_4^* + O^* \xrightarrow[\text{Co/HAP}]{\text{TS}_1} CH_3^* + OH^*$ S-40

S-7.2.3 $CH_4^* + O^* \xrightarrow[\text{Ni/HAP}]{\text{TS}_1} CH_3^* + OH^*$ S-41

S-7.2.4 $CH_4^* + O^* \xrightarrow[\text{Cu/HAP}]{\text{TS}_1} CH_3^* + OH^*$ S-42

S-7.3 Reaction Path for Methanol Formation S-43

S-7.3.1 $CH_3^* + OH^* \xrightarrow[\text{Fe/HAP}]{\text{TS}_2} CH_3OH^*$ S-43

S-7.3.2 $CH_3^* + OH^* \xrightarrow[\text{Co/HAP}]{TS_2} CH_3OH^*$ S-44
S-7.3.3 $CH_3^* + OH^* \xrightarrow[\text{Ni/HAP}]{TS_2} CH_3OH^*$ S-45
S-7.3.4 $CH_3^* + OH^* \xrightarrow[\text{Cu/HAP}]{TS_2} CH_3OH^*$ S-46
S-7.4 Reaction Path for Methanol Dissociation S-47
S-7.4.1 $CH_3OH^* \xrightarrow[\text{Fe/HAP}]{TS_1} CH_3^*/TM + OH^*$ S-47
S-7.4.2 $CH_3OH^* \xrightarrow[\text{Co/HAP}]{TS_1} CH_3^*/TM + OH^*$ S-48
S-7.4.3 $CH_3OH^* \xrightarrow[\text{Ni/HAP}]{TS_1} CH_3^*/TM + OH^*$ S-49
S-7.4.4 $CH_3OH^* \xrightarrow[\text{Cu/HAP}]{TS_1} CH_3^*/TM + OH^*$ S-50
S-7.5 Reaction Path for Diffusion of the CH_3 Fragment S-51
S-7.5.1 $CH_3^*/TM \xrightarrow[\text{Fe/HAP}]{TS_2} CH_3^*/O$ S-51
S-7.5.2 $CH_3^*/TM \xrightarrow[\text{Co/HAP}]{TS_2} CH_3^*/O$ S-52
S-7.5.3 $CH_3^*/TM \xrightarrow[\text{Ni/HAP}]{TS_2} CH_3^*/O$ S-53
S-7.5.4 $CH_3^*/TM \xrightarrow[\text{Cu/HAP}]{TS_2} CH_3^*/O$ S-54

S-8 Transition State Structures and Vibrational Frequencies S-55

S-9 Additional Computational Parameters and Input Files S-61
S-9.1 Input File for the Structural Optimization Calculations S-62
S-9.2 Input File for the CI-NEB Calculations S-63

References S-64

S-1 Introduction

This supplementary document provides detailed technical information to support the findings and discussions presented in the main manuscript. Specifically, it includes (*i*) technical details on the selected PAW projectors (Section S-2), (*ii*) a comprehensive evaluation of the slab models used for modeling catalyst surfaces (Section S-3), (*iii*) a complete set of structures used to explore various adsorption sites on the catalyst surface (Section S-4), (*iv*) selected properties used for evaluating HAP-based systems (Section S-5 and S-6), (*v*) a detailed description of the reaction paths and activation energy barriers (Section S-7), (*vi*) transition state structures and associated vibrational frequencies (Section S-8), (*vii*) and finally, examples of input files used for calculations of structural optimization and localization of transition states (Section S-9).

S-2 Additional PAW Projector Details

As described in the main article, all calculations were performed using the Vienna *Ab initio* Simulation Package (VASP), version 5.4.4.[1,2] Within this software, the Kohn–Sham (KS) equations are solved using the frozen-core projector augmented wave (PAW) method[3,4] to model interactions between core and valence electrons, with the KS states expanded in plane-wave basis sets. In Table S-1, we provide additional details on the specifications of the PAW projectors of each chemical species selected in this study.

Table S-1: Technical details of the PAW-PBE projectors selected for this study. Recommended cutoff energy for the plane-wave basis set, `ENMAX` (eV), and number of valence electrons, `ZVAL`.

Element	PAW Projector	`ENMAX`	`ZVAL`	Valence
H	H_GW 21Apr2008	300.000	1	$1s^1$
C	C_GW_new 19Mar2012	413.992	4	$2s^2 2p^2$
P	P_GW 19Mar2012	255.040	5	$3s^2 3p^3$
O	O_GW_new 19Mar2012	434.431	6	$2s^2 2p^4$
Fe	Fe_GW 31Mar2010	321.007	8	$3d^7 4s^1$
Co	Co_GW 31Mar2010	323.400	9	$3d^8 4s^1$
Ni	Ni_GW 31Mar2010	357.323	10	$3d^9 4s^1$
Ca	Ca_sv_GW 31Mar2010	281.430	10	$3s^2 3p^6 3d^2$
Cu	Cu_GW 19May2006	417.039	11	$3d^{10} 4s^1$

S-3 Additional Computational Tests on Selection of Slab Models

The construction of slab models to investigate single-atom transition metal catalysts supported on hydroxyapatite (TM/HAP SACs) was based on our previous experience using hydroxyapatite as substrate.[5–7] The slab model used to model the termination of the stoichiometric surface HAP(0001) is depicted in Figure S-1a. This model presents four formula units $Ca_5(PO_4)_3(OH)$ stacked along the z-axis, a total of 88 atoms, lattice parameters $a_0 = b_0 = 9.497$ Å, and a thickness of 12.88 Å separated by vacuum region of 15 Å.

In search of an appropriate model for the TM/HAP catalysts, we tested three different sets of parameters: (i) the TM-SAC adsorbed on one side of the HAP(0001) slab model with dipole correction; (ii) the TM-SAC adsorbed on one side of the slab model HAP(0001) without dipole correction; and (iii) the TM-SAC adsorbed on both sides of the HAP(0001) slab model without dipole correction. The atomic arrangement for these confirations are depicted in in Figure S-1b and S-1c.

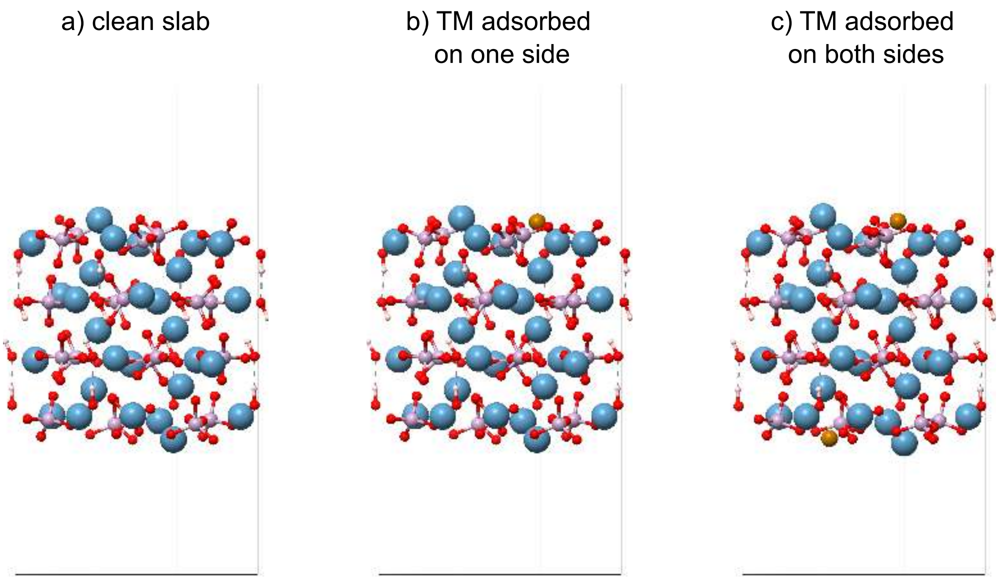

Figure S-1: Slab models evaluated for modeling the HAP(0001) surface and TM/HAP catalysts. Light blue, pink, red, and white spheres represent Ca, P, and O atoms in the substrate, respectively; while ocher spheres represent the Fe SAC.

As shown in Table S-2, the impact on the values of adsorption energy for all TM selected for this study is minimum. Therefore, we selected the set of parameters that had the lowest computational cost: the TM-SAC adsorbed on one side of the HAP(0001) slab model without dipole correction. This configuration is also advantageous for the conduction of climbing-image nudged elastic band (CI-NEB) calculations.

Table S-2: Adsorption energies for the TM-SACs on the HAP(0001) surface. Three distinct configurations were evaluated: (i) the TM-SAC adsorbed on one side of the slab model with dipole correction (Slab Model I); (ii) the TM-SAC adsorbed on one side of the slab model without dipole correction (Slab Model II), and (iii) the TM-SAC adsorbed on both sides of the slab model without dipole correction (Slab Model III).

TM/HAP	Slab Model I	Slab Model II	Slab Model III
Fe/HAP	−1.19 eV	−1.20 eV	−1.28 eV
Co/HAP	−1.47 eV	−1.47 eV	−1.46 eV
Ni/HAP	−2.19 eV	−2.19 eV	−2.19 eV
Cu/HAP	−0.73 eV	−0.75 eV	−0.73 eV

S-4 Complete Set of Optimized Adsorption Structures

The Figures S-2 to S-25 provide a visual representation of the entire set of adsorption structures optimized to find the lowest energy configurations. The atoms are colored as follows: light blue, pink, and red spheres represent Ca, P, and O atoms in the substrate, respectively; brown and white spheres indicate C and H atoms; light green spheres represent O atoms from the O_2 molecule; and ocher, dark blue, silver, and dark green spheres represent Fe, Co, Ni, and Cu transition metal SACs, respectively.

S-4.1 Optimized Configurations for TM-SACs on HAP

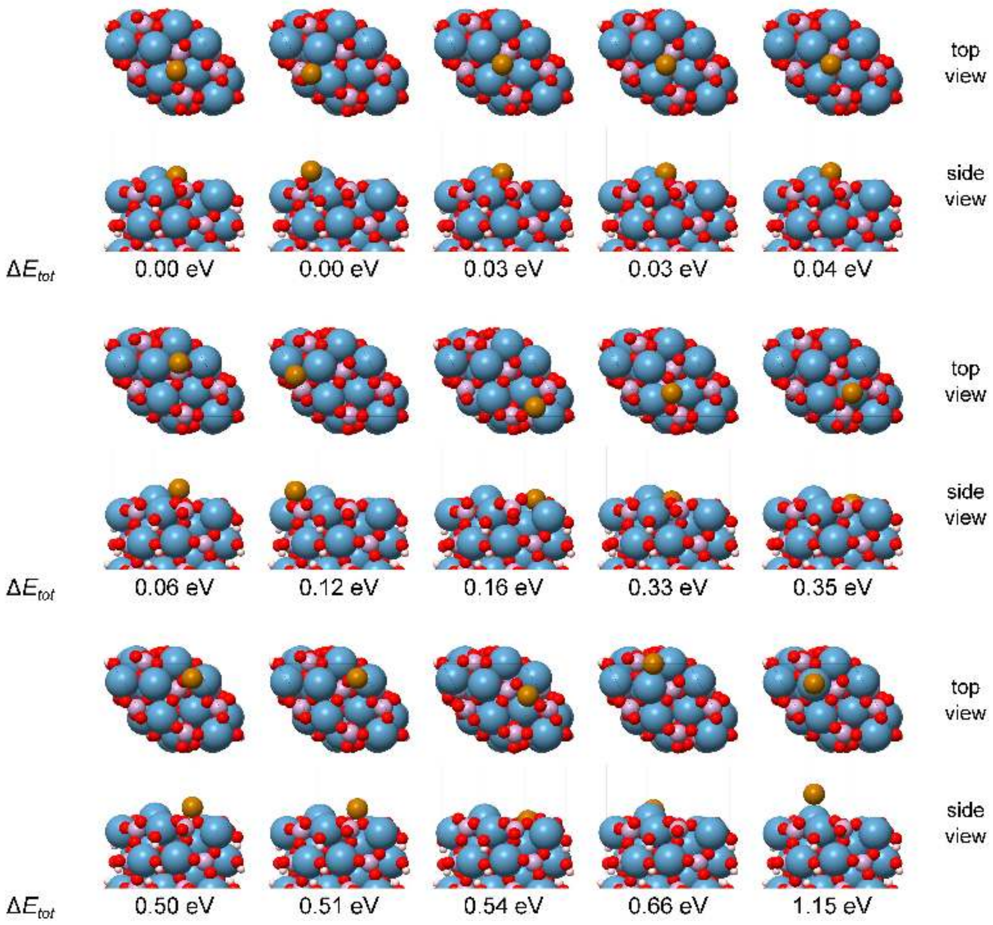

Figure S-2: Optimized adsorption configurations for Fe on the HAP(0001) surface. Below each model is the total energy relative to the lowest energy configuration.

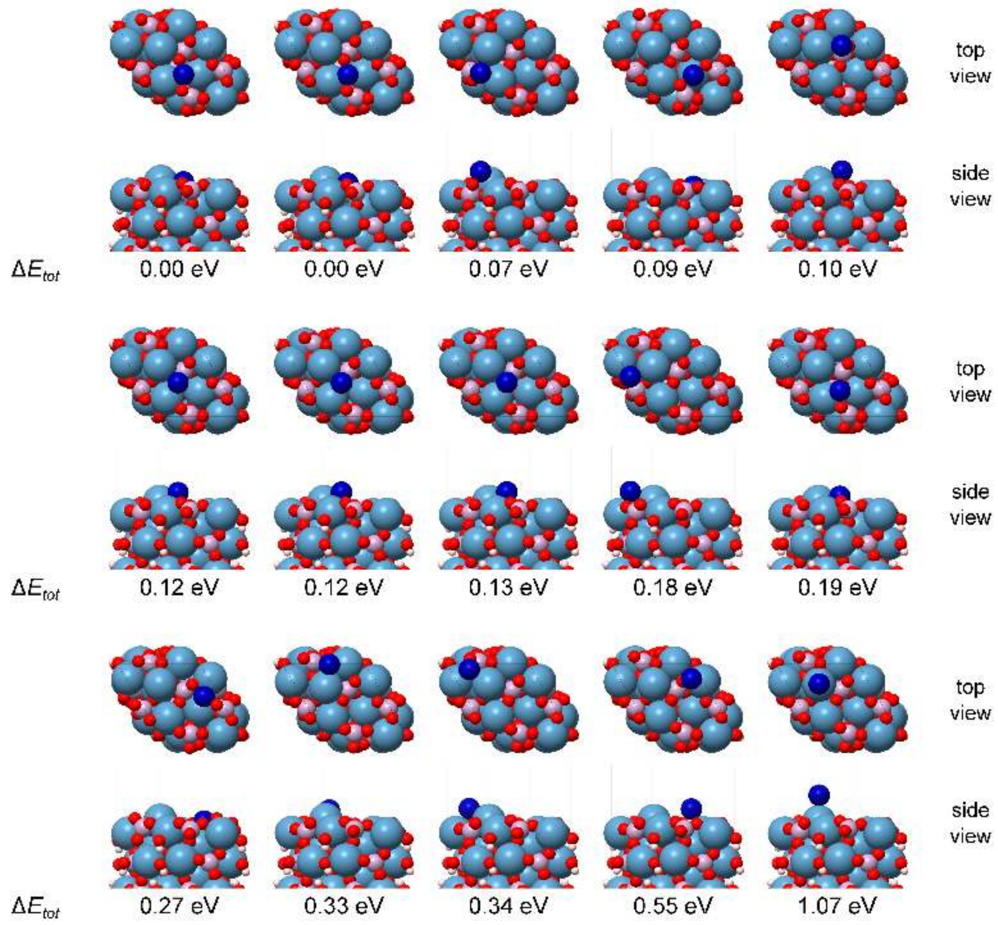

Figure S-3: Optimized adsorption configurations for Co on the HAP(0001) surface. Below each model is the total energy relative to the lowest energy configuration.

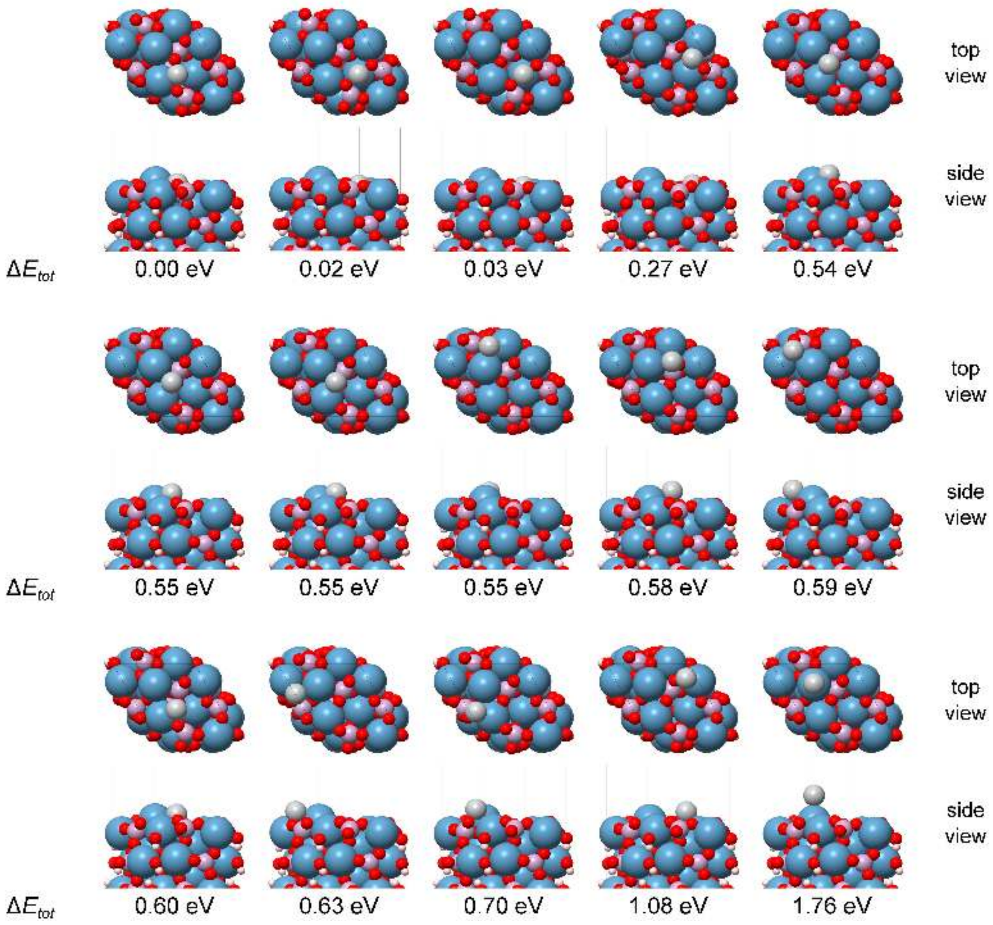

Figure S-4: Optimized adsorption configurations for Ni on the HAP(0001) surface. Below each model is the total energy relative to the lowest energy configuration.

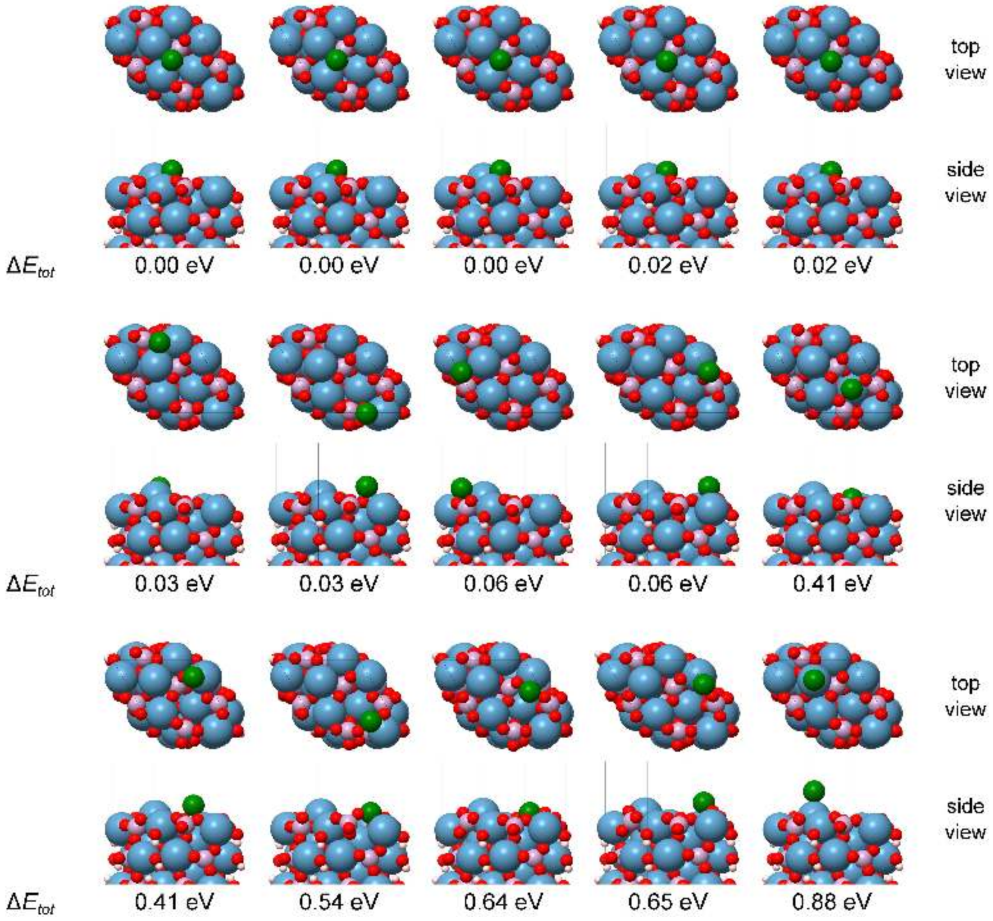

Figure S-5: Optimized adsorption configurations for Cu on the HAP(0001) surface. Below each model is the total energy relative to the lowest energy configuration.

S-4.2 Optimized Configurations for O_2 on TM-SACs

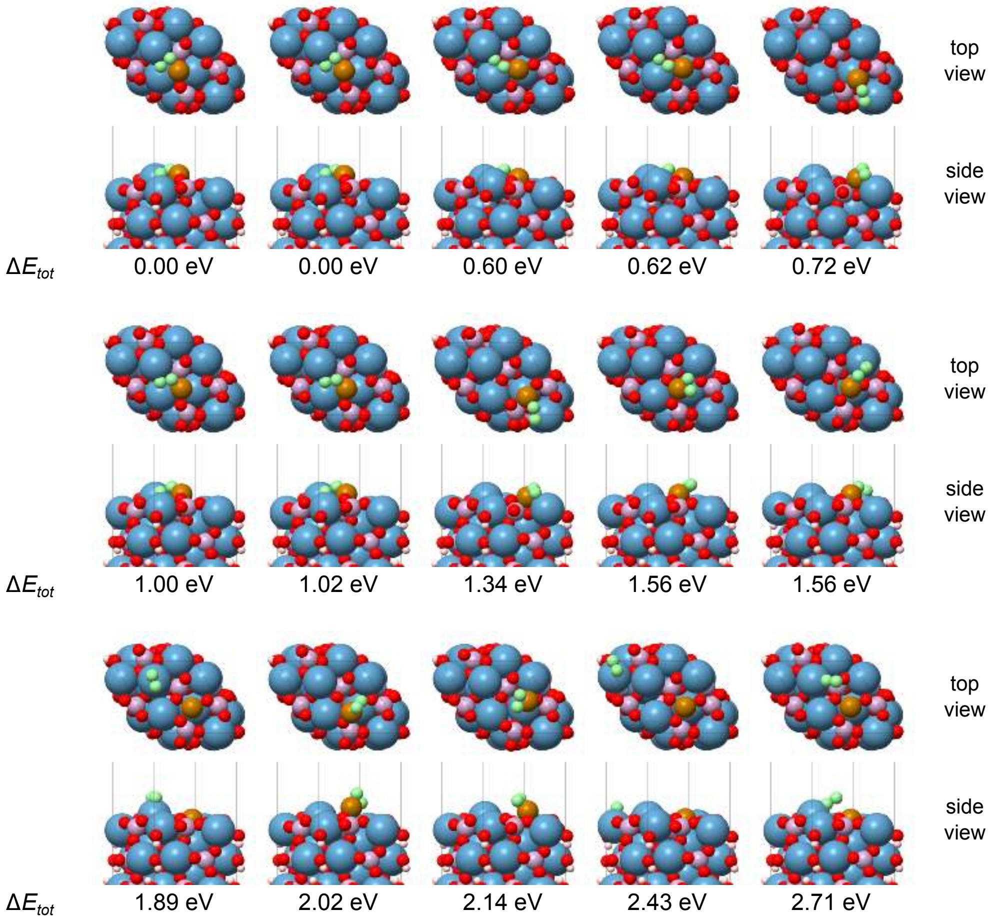

Figure S-6: Optimized adsorption configurations for O_2 on the Fe/HAP(0001) surface. Below each model is the total energy relative to the lowest energy configuration.

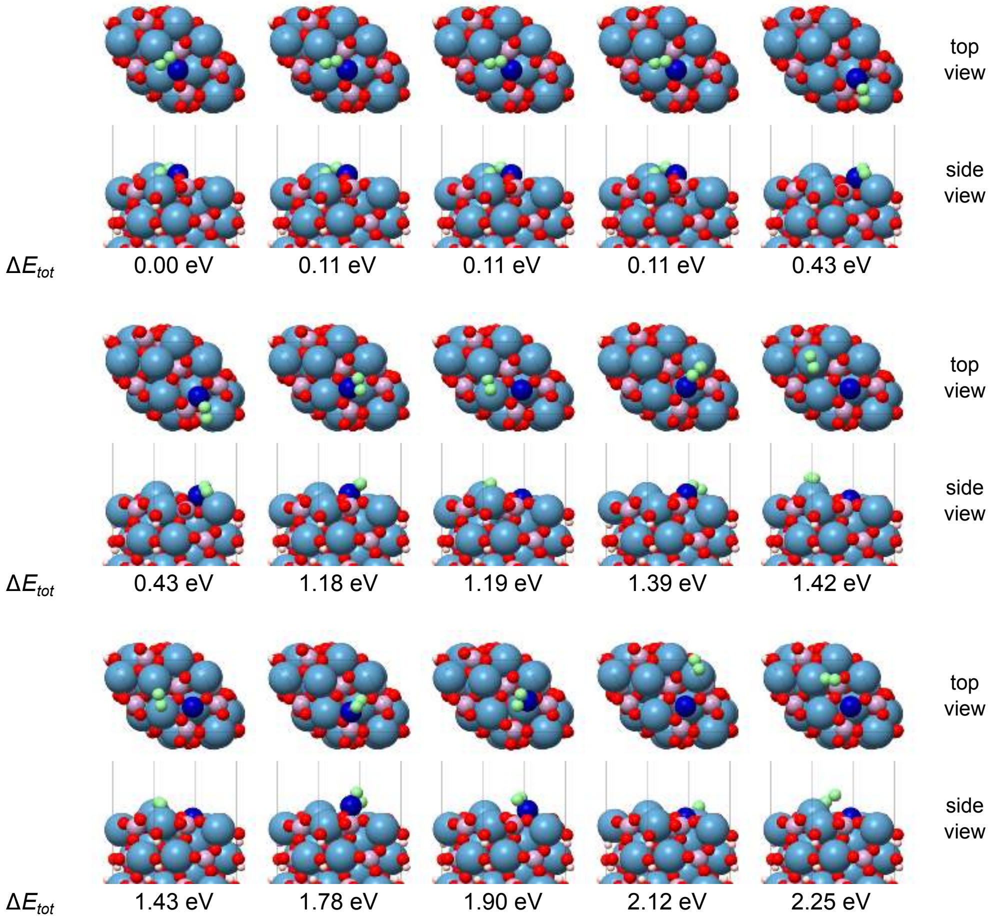

Figure S-7: Optimized adsorption configurations for O_2 on the Co/HAP(0001) surface. Below each model is the total energy relative to the lowest energy configuration.

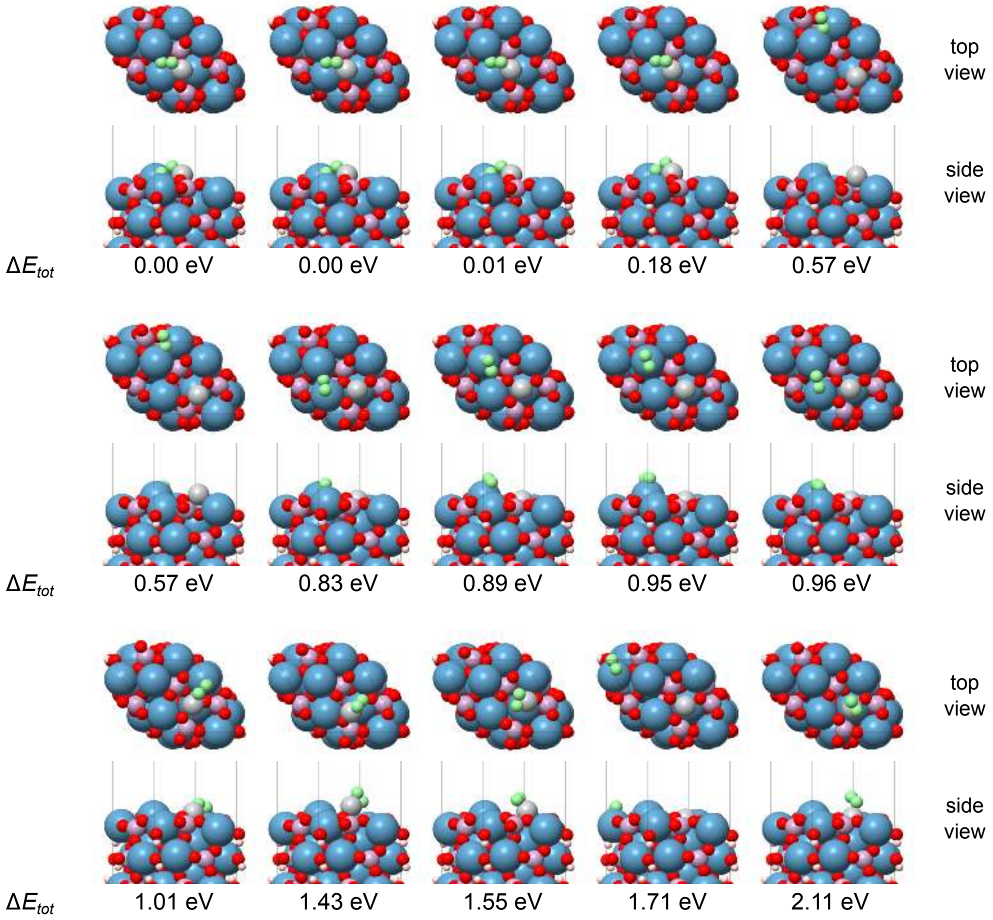

Figure S-8: Optimized adsorption configurations for O_2 on the Ni/HAP(0001) surface. Below each model is the total energy relative to the lowest energy configuration.

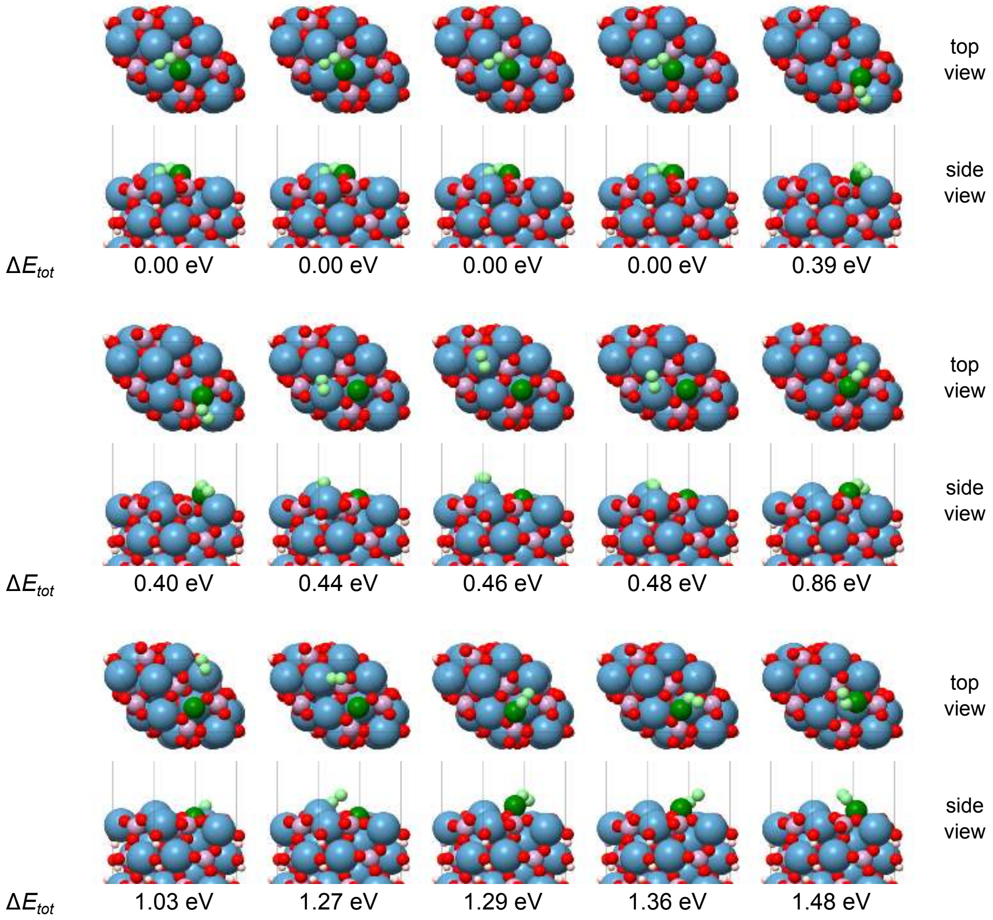

Figure S-9: Optimized adsorption configurations for O_2 on the Cu/HAP(0001) surface. Below each model is the total energy relative to the lowest energy configuration.

S-4.3 Optimized Configurations for Dissociated O_2 on TM-SACs

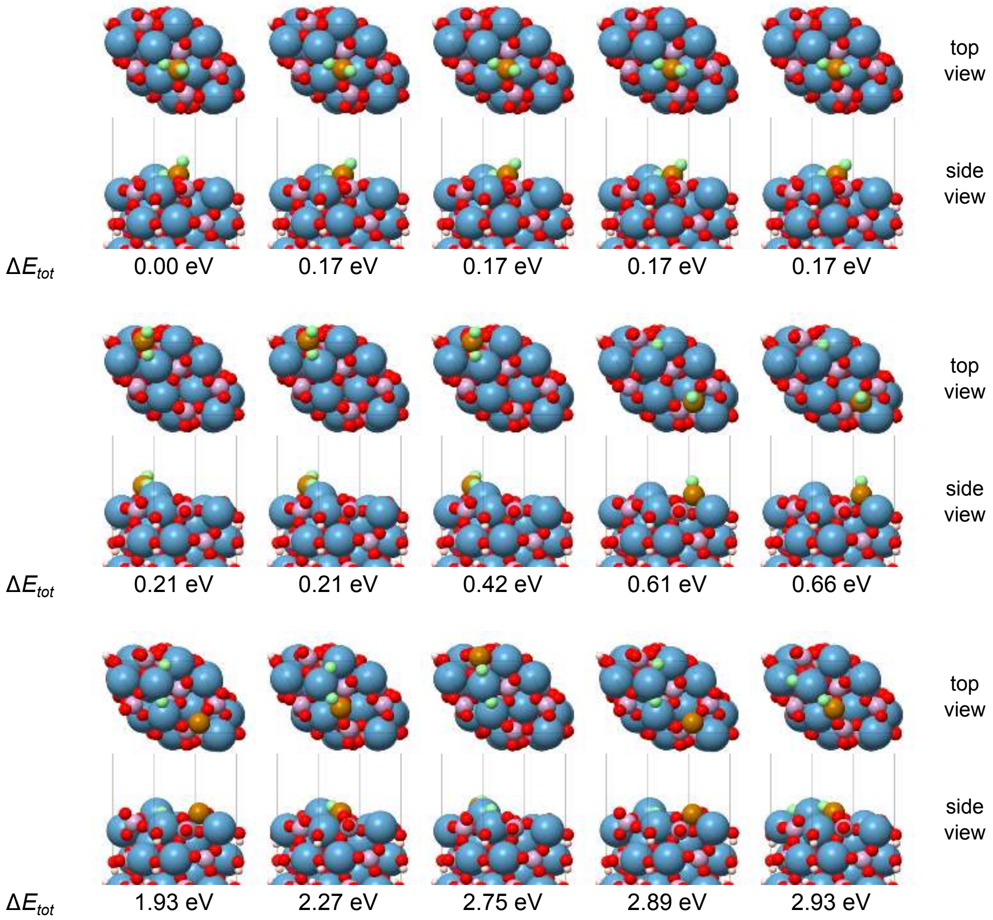

Figure S-10: Optimized adsorption configurations for O+O on the Fe/HAP(0001) surface. Below each model is the total energy relative to the lowest energy configuration.

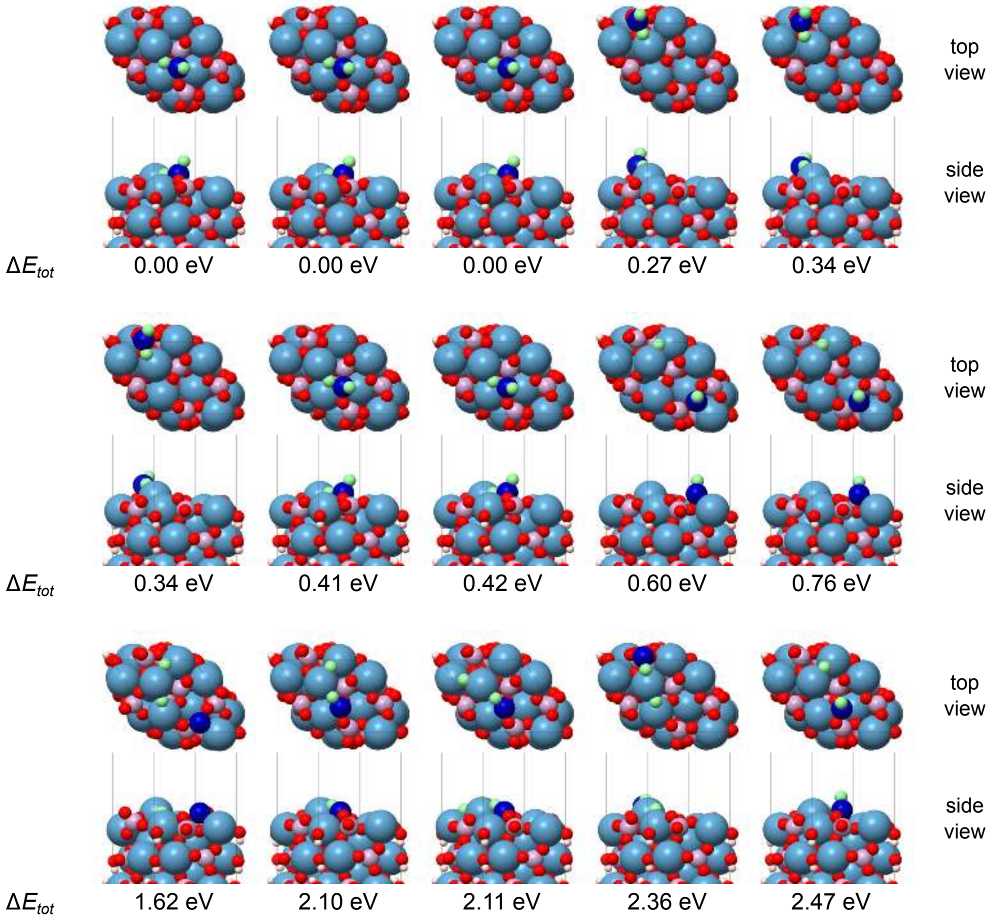

Figure S-11: Optimized adsorption configurations for O+O on the Co/HAP(0001) surface. Below each model is the total energy relative to the lowest energy configuration.

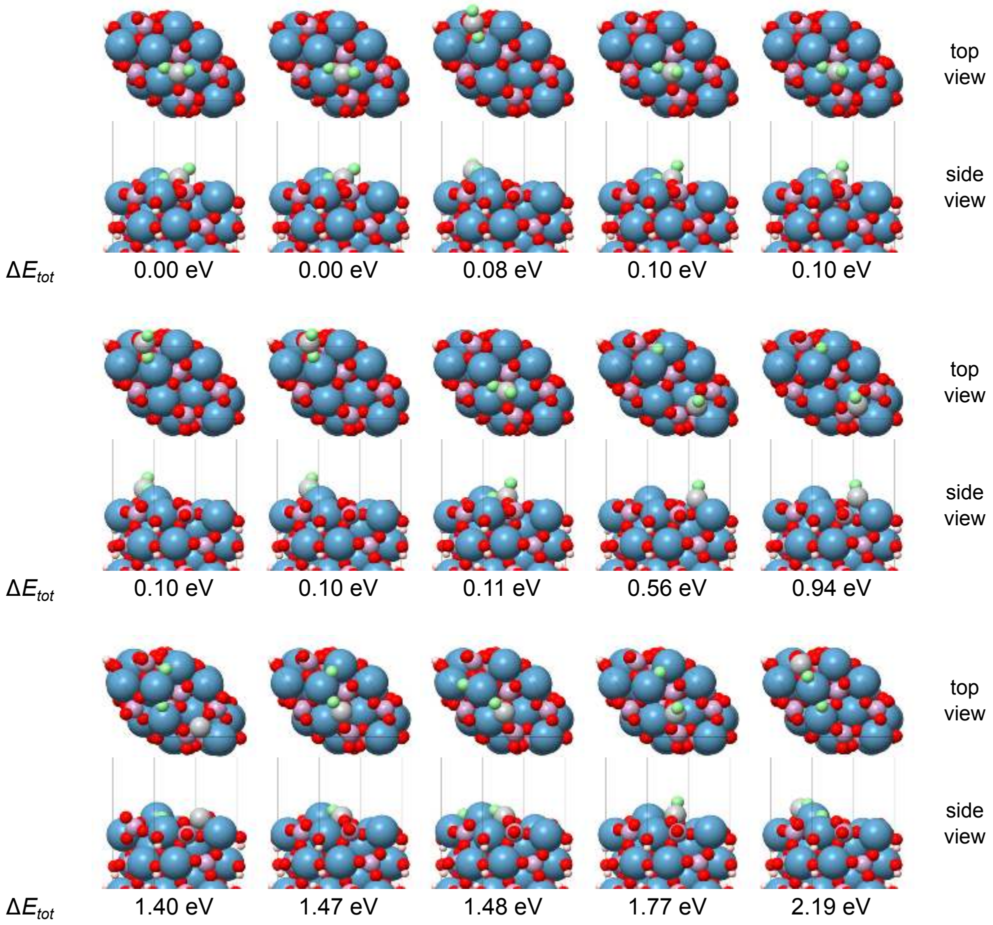

Figure S-12: Optimized adsorption configurations for O+O on the Ni/HAP(0001) surface. Below each model is the total energy relative to the lowest energy configuration.

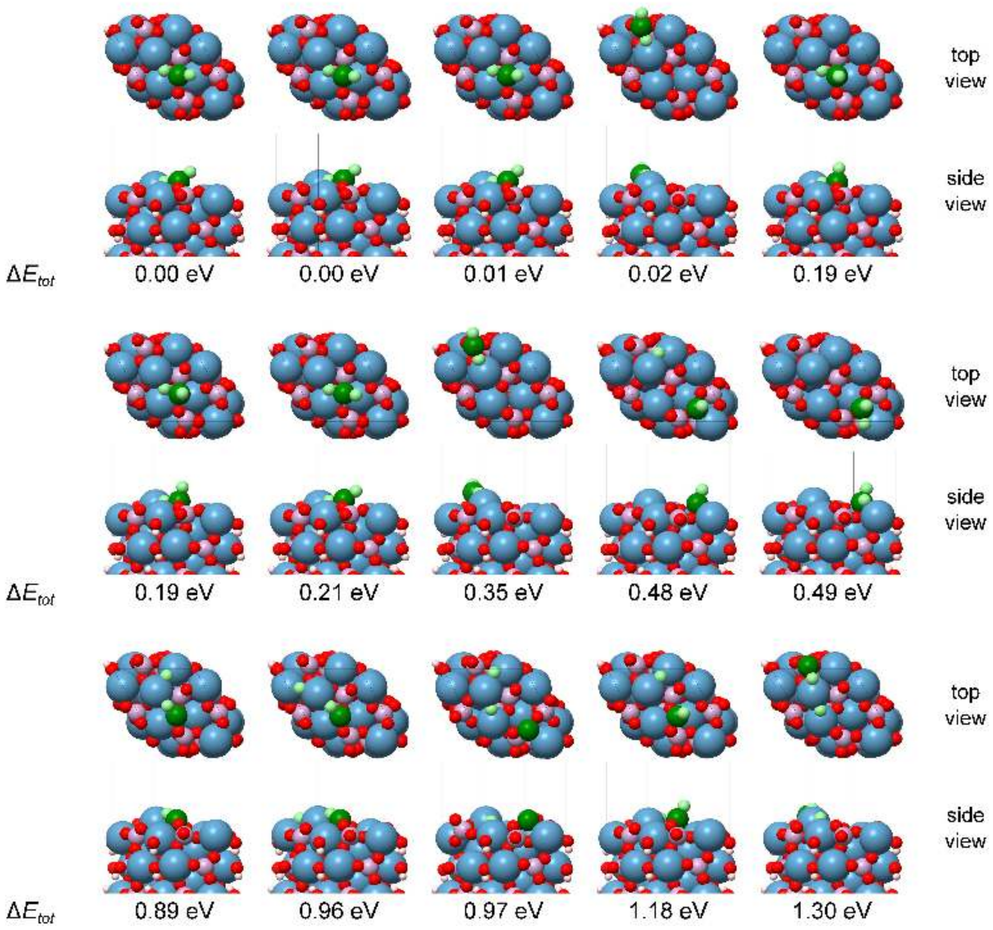

Figure S-13: Optimized adsorption configurations for O+O on the Cu/HAP(0001) surface. Below each model is the total energy relative to the lowest energy configuration.

S-4.4 Optimized Configurations for CH_4 on TM-SACs

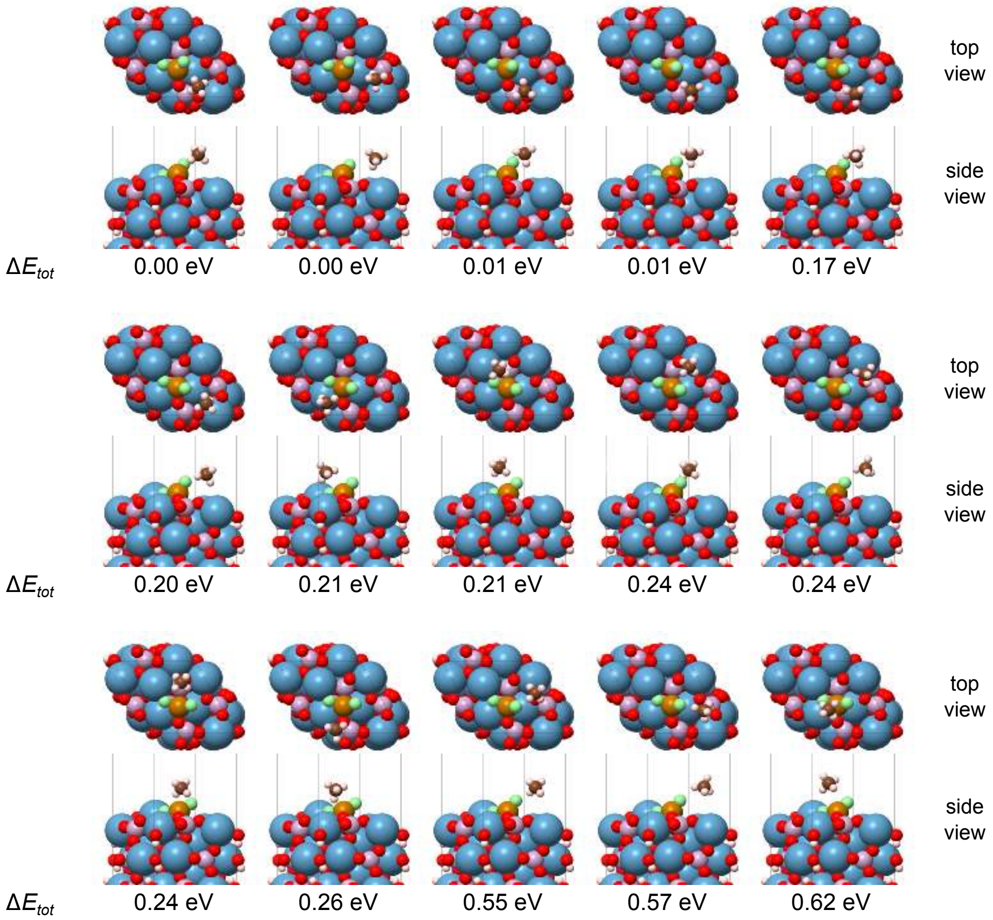

Figure S-14: Optimized adsorption configurations for CH_4 on the Fe/HAP(0001) surface. Below each model is the total energy relative to the lowest energy configuration.

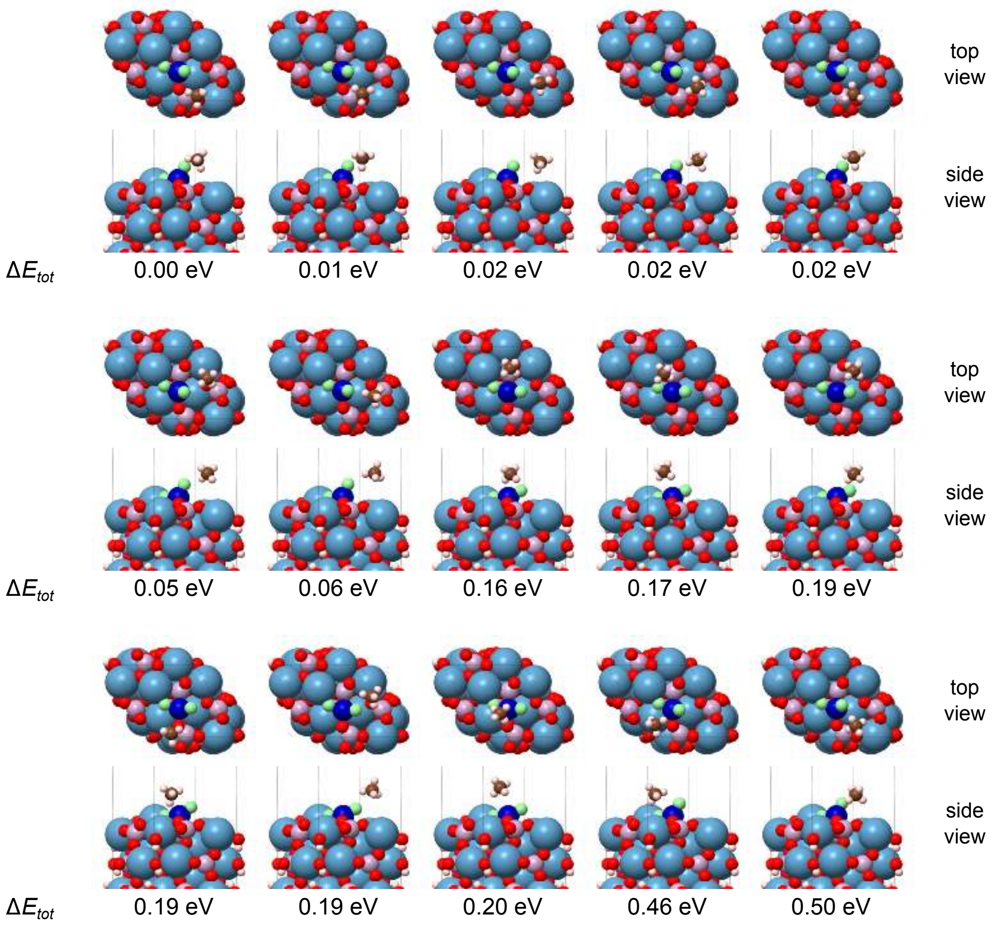

Figure S-15: Optimized adsorption configurations for CH_4 on the Co/HAP(0001) surface. Below each model is the total energy relative to the lowest energy configuration.

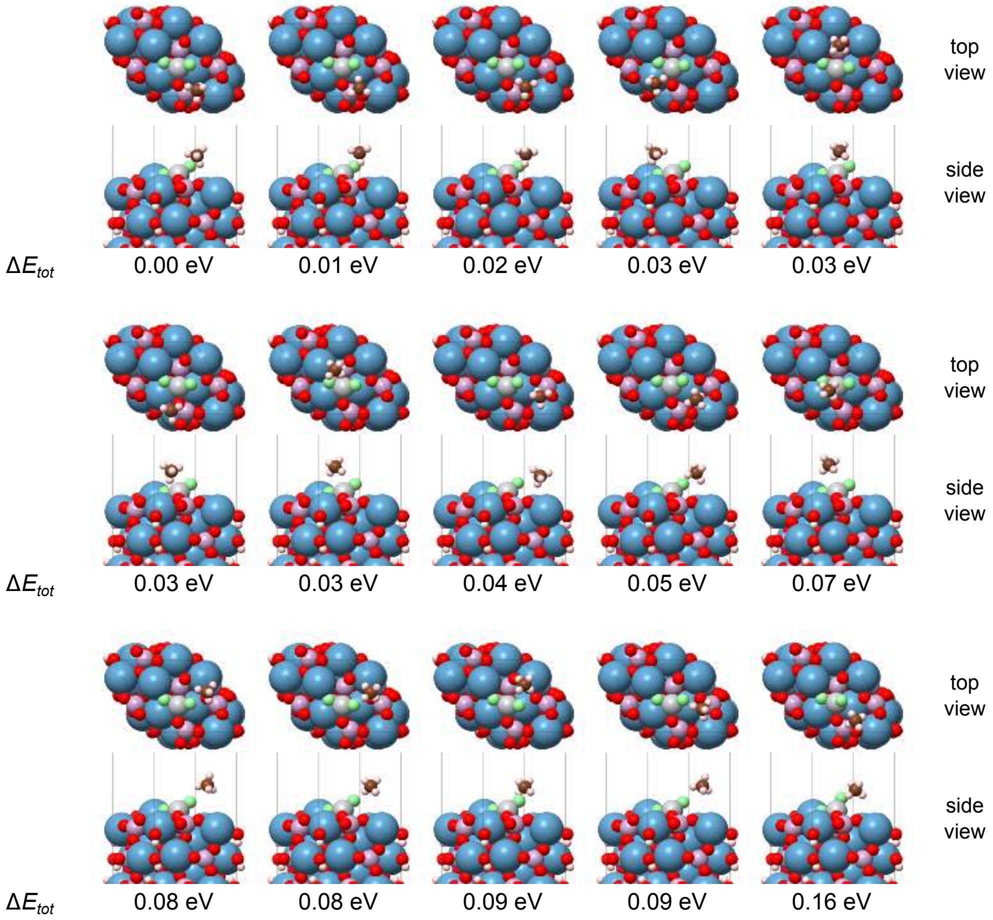

Figure S-16: Optimized adsorption configurations for CH_4 on the Ni/HAP(0001) surface. Below each model is the total energy relative to the lowest energy configuration.

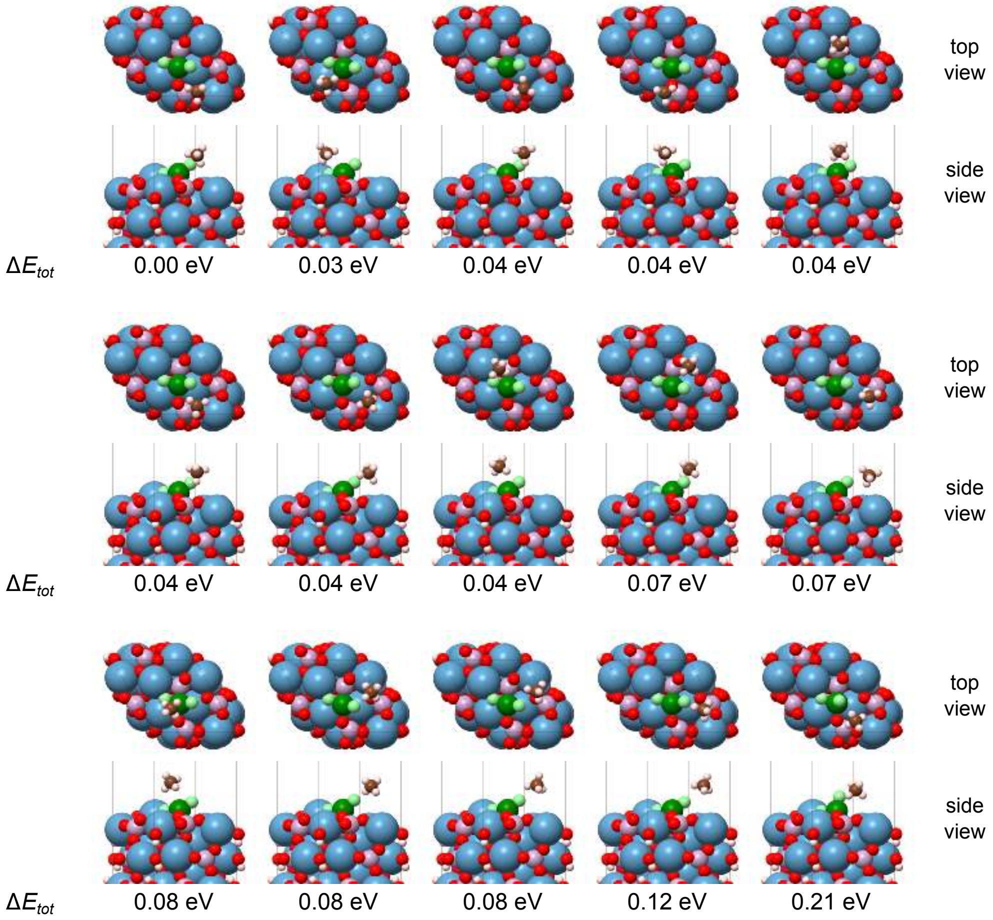

Figure S-17: Optimized adsorption configurations for CH_4 on the Cu/HAP(0001) surface. Below each model is the total energy relative to the lowest energy configuration.

S-4.5 Optimized Configurations for Methanol on TM-SACs

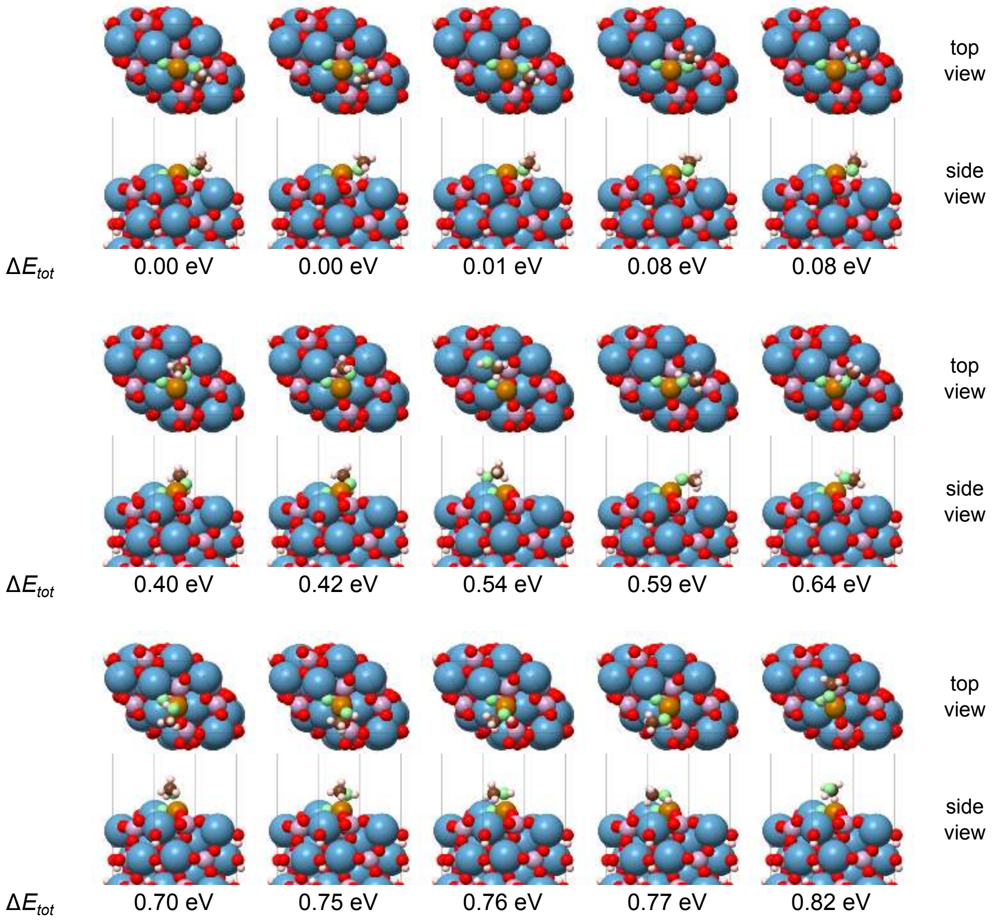

Figure S-18: Optimized adsorption configurations for CH_3OH on the Fe/HAP(0001) surface. Below each model is the total energy relative to the lowest energy configuration.

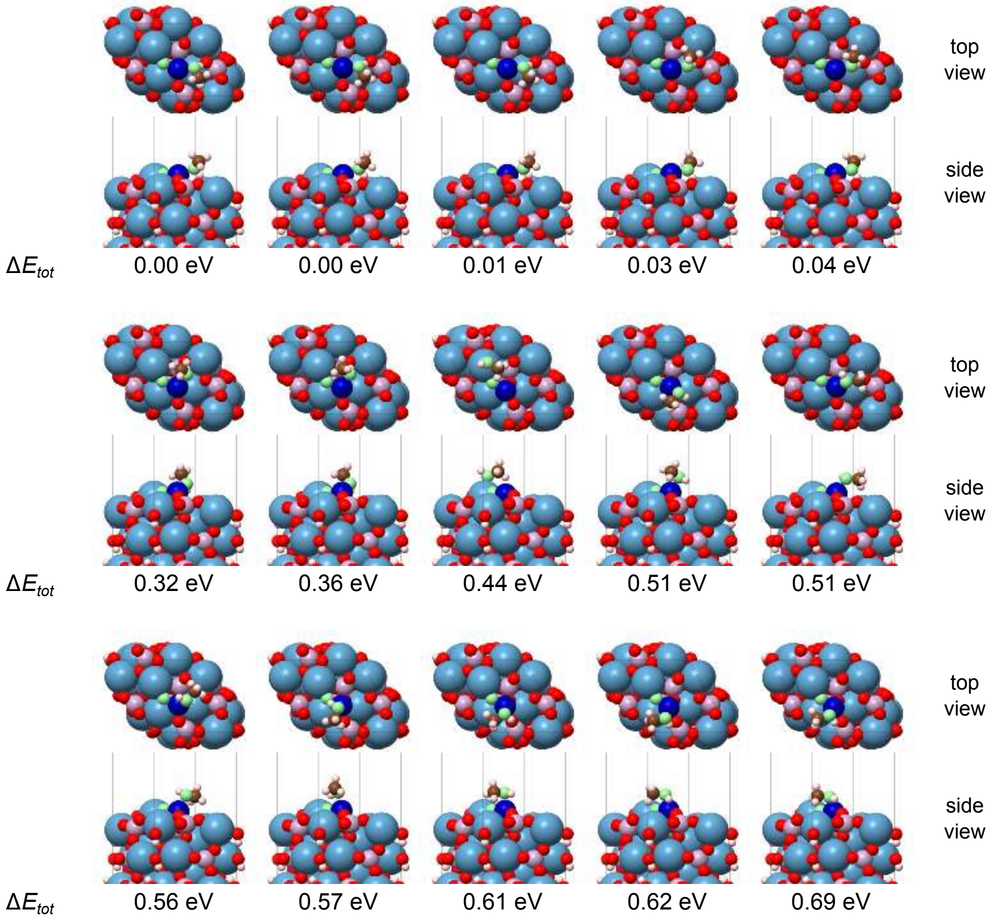

Figure S-19: Optimized adsorption configurations for CH_3OH on the Co/HAP(0001) surface. Below each model is the total energy relative to the lowest energy configuration.

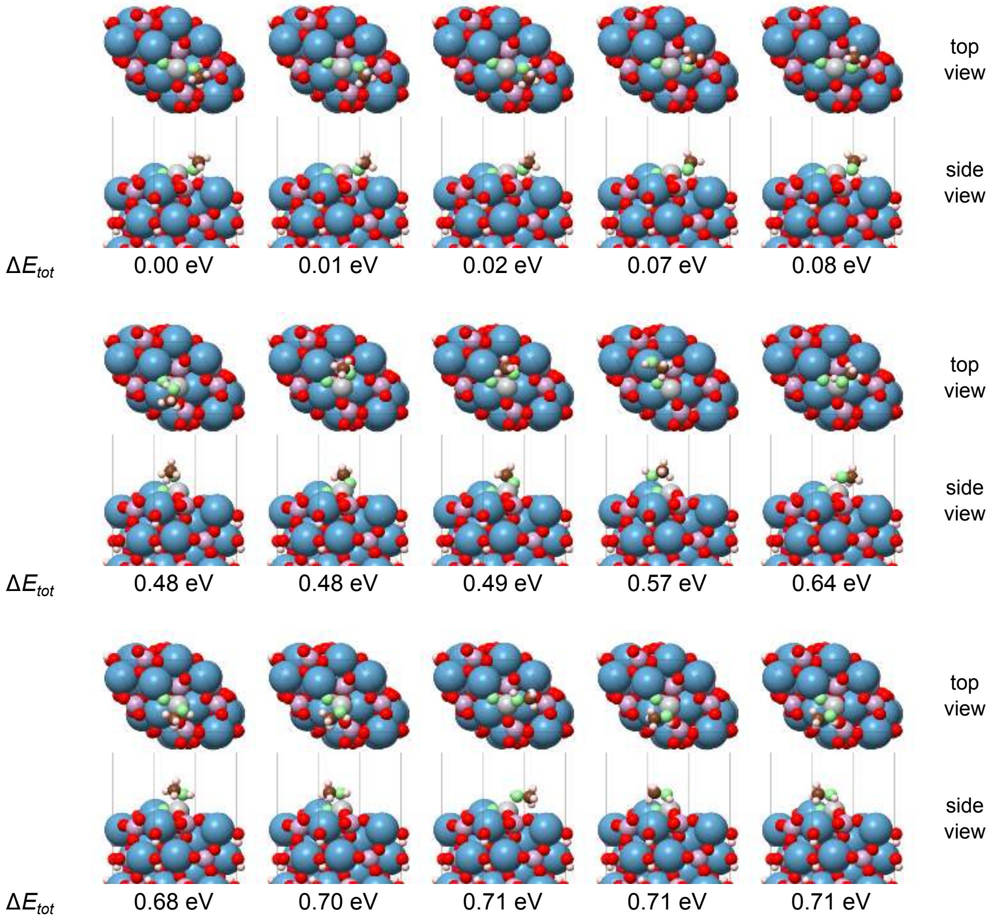

Figure S-20: Optimized adsorption configurations for CH_3OH on the Ni/HAP(0001) surface. Below each model is the total energy relative to the lowest energy configuration.

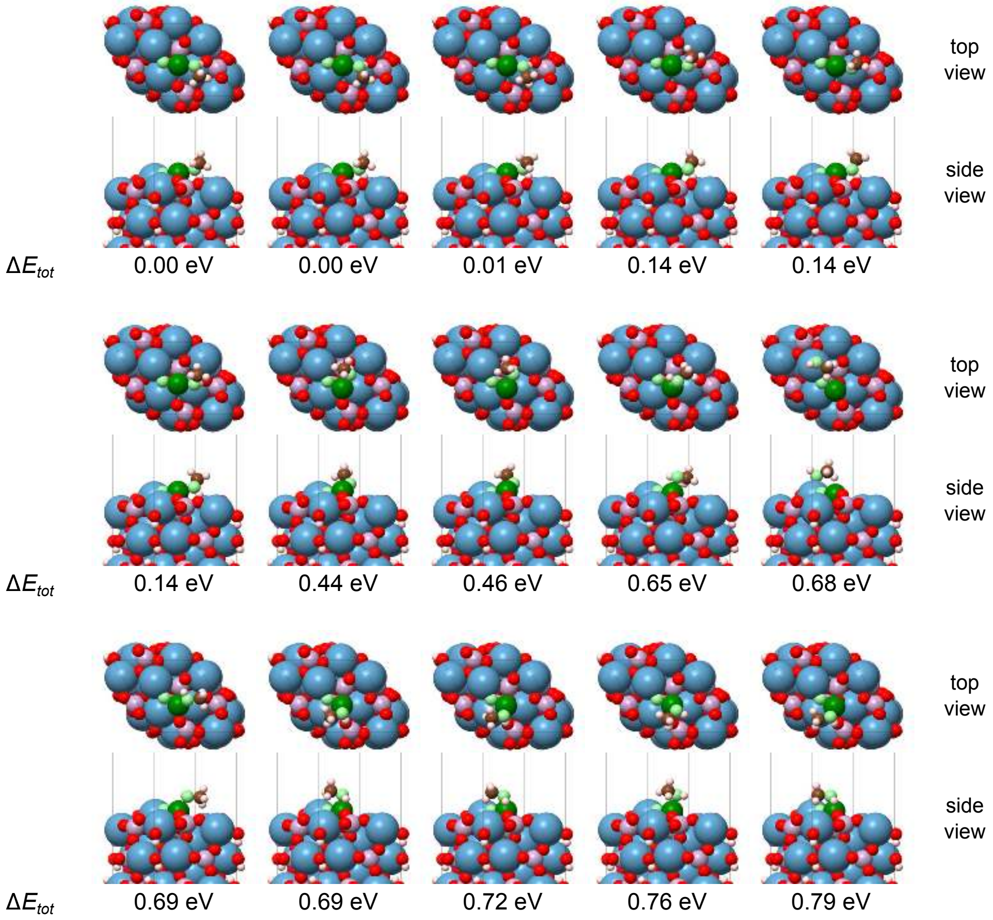

Figure S-21: Optimized adsorption configurations for CH_3OH on the Cu/HAP(0001) surface. Below each model is the total energy relative to the lowest energy configuration.

S-4.6 Optimized Configurations for Dissociated Methanol on TM-SACs

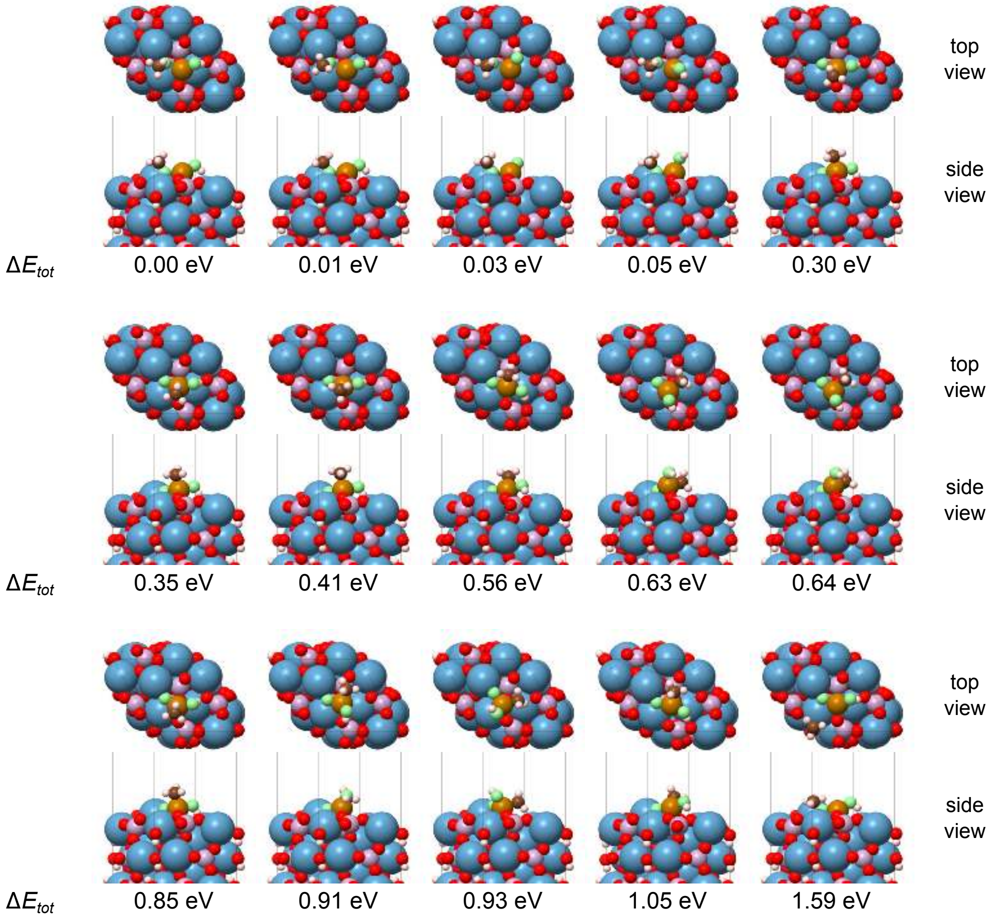

Figure S-22: Optimized adsorption configurations for CH_3+OH on the Fe/HAP(0001) surface. Below each model is the total energy relative to the lowest energy configuration.

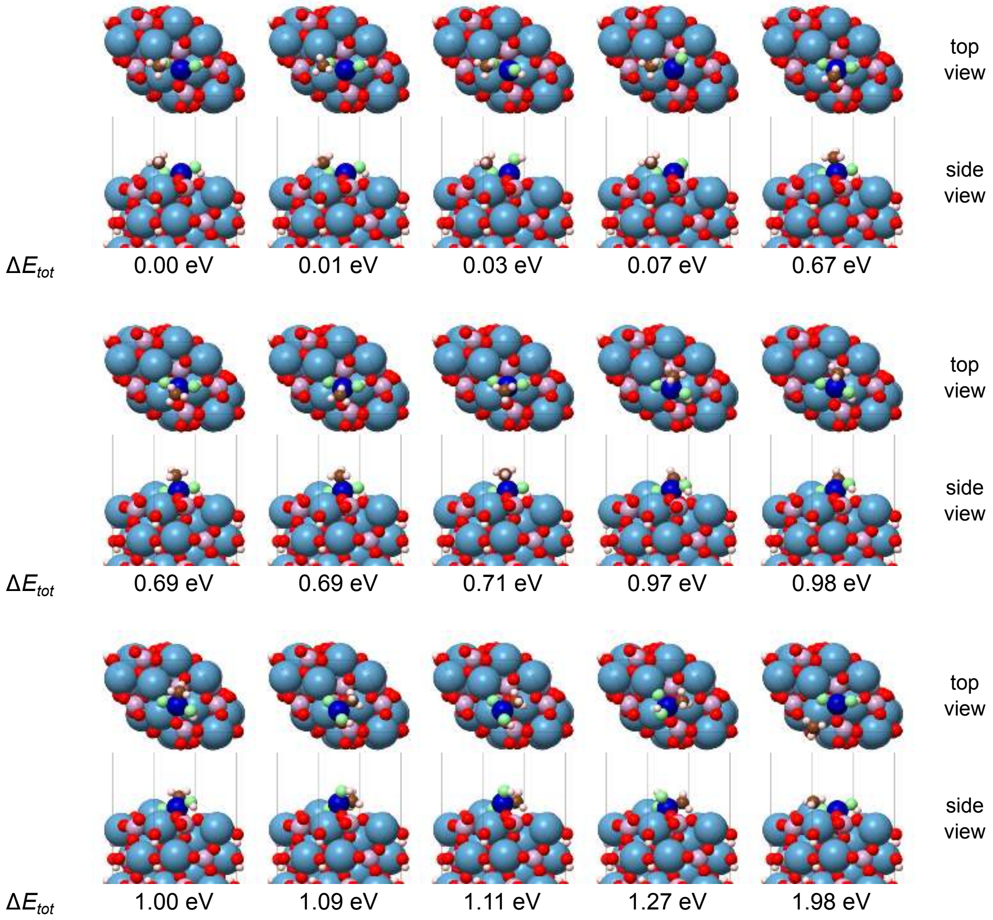

Figure S-23: Optimized adsorption configurations for CH_3+OH on the Co/HAP(0001) surface. Below each model is the total energy relative to the lowest energy configuration.

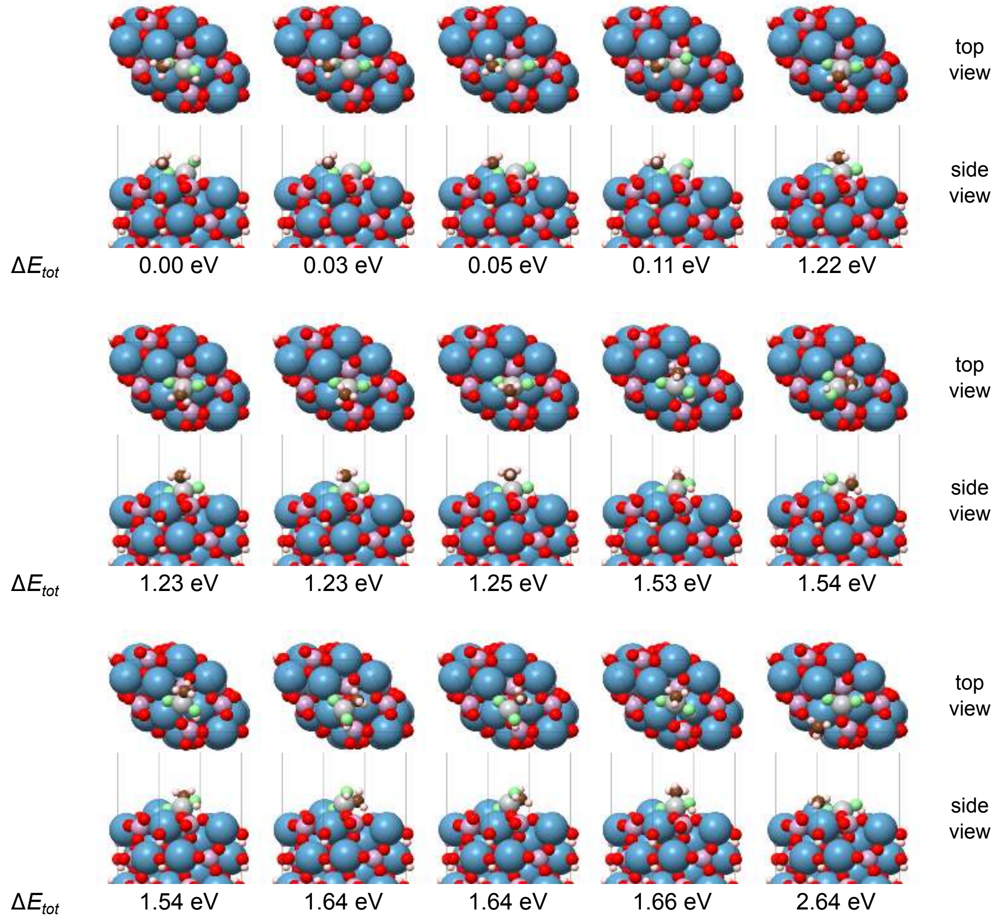

Figure S-24: Optimized adsorption configurations for CH_3+OH on the Ni/HAP(0001) surface. Below each model is the total energy relative to the lowest energy configuration.

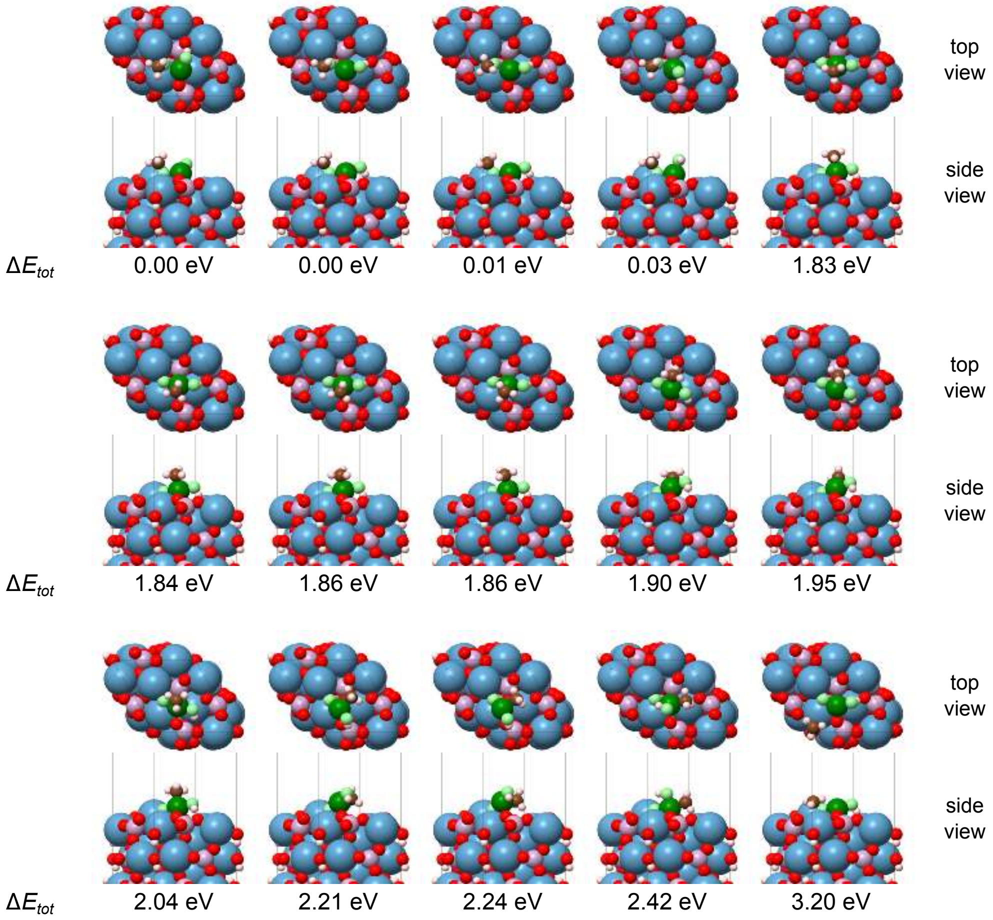

Figure S-25: Optimized adsorption configurations for CH_3+OH on the Cu/HAP(0001) surface. Below each model is the total energy relative to the lowest energy configuration.

S-5 Electronic Properties of HAP-based Systems

Table S-3: Average net atomic charges of HAP(0001) and TM/HAP substrates.

Substrate	TM	Ca	P	O	H
HAP(0001)		1.46	1.50	−0.94	0.35
Fe/HAP	−0.10	1.45	1.50	−0.93	0.35
Co/HAP	−0.16	1.45	1.49	−0.92	0.35
Ni/HAP	−0.17	1.45	1.49	−0.93	0.35
Cu/HAP	−0.13	1.45	1.50	−0.93	0.35

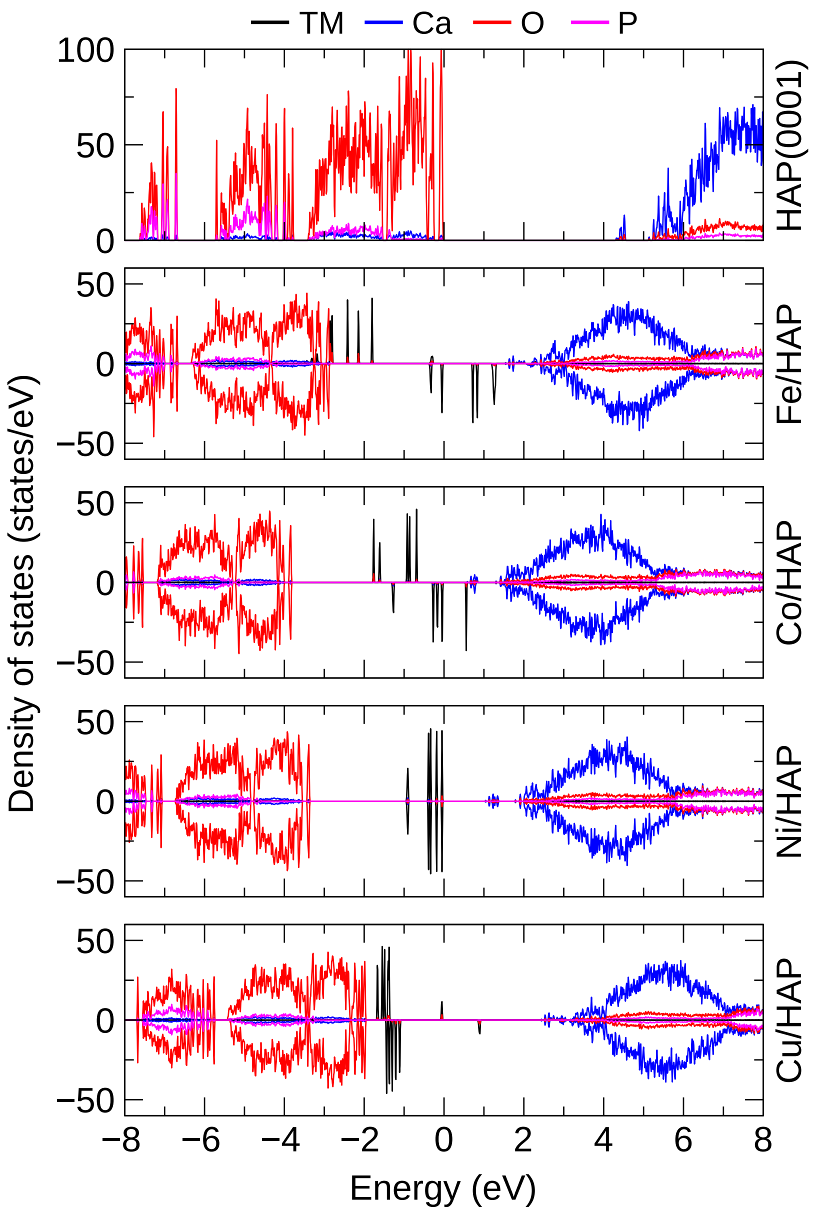

Figure S-26: Density of states of HAP(0001) and TM/HAP substrates.

Table S-4: Average net atomic charges of HAP(0001) and O+O/TM/HAP substrates.

Substrate	TM	Ca	P	O^{HAP}	O^{O_2}	H
HAP(0001)		1.46	1.50	−0.94		0.35
O+O/Fe/HAP	1.27	1.46	1.50	−0.93	−0.77	0.35
O+O/Co/HAP	1.19	1.46	1.50	−0.93	−0.72	0.35
O+O/Ni/HAP	1.12	1.46	1.50	−0.93	−0.70	0.35
O+O/Cu/HAP	1.09	1.46	1.50	−0.93	−0.65	0.35

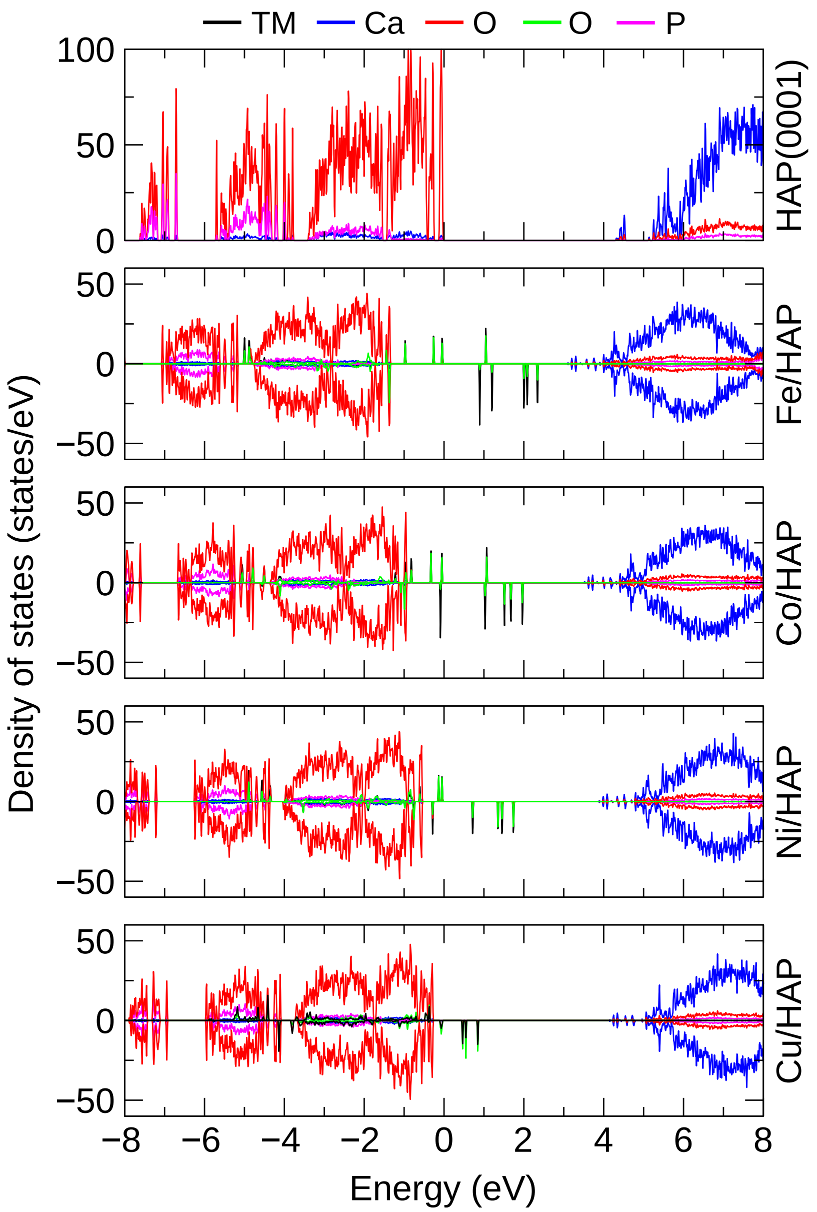

Figure S-27: Density of states of HAP(0001) and O+O/TM/HAP substrates. Oxygen atoms of phosphate groups are presented in red while oxygen atoms of the dissociated O_2 molecule are presented in green.

S-6 Adsorption Properties of HAP-based Systems

To characterize the strength of the interaction between the TM-SACs and molecules adsorbed on the HAP(0001) surface, we evaluated the adsorption energy as:

$$E_{ad} = E_{tot}^{\text{adsorbate/substrate}} - E_{tot}^{\text{adsorbate}} - E_{tot}^{\text{substrate}}\,, \tag{1}$$

where $E_{tot}^{\text{adsorbate/substrate}}$ is the total energy of the adsorbed system, $E_{tot}^{\text{adsorbate}}$ is the total energy of the isolated adsorbate in gas-phase (TM-SACs, molecules, and molecular fragments), and $E_{tot}^{\text{substrate}}$ is the total energy of the substrate.

Table S-5: Adsorption energies of selected adsorbates on the HAP(0001). To facilitate the definition of adsorbate and substrate for each system, species highlighted in bold represents the adsorbate, while species in regular text indicate the substrate.

Adsorption System	E_{ad} (eV)
**Fe**/HAP	−1.48
**Co**/HAP	−1.47
**Ni**/HAP	−2.19
**Cu**/HAP	−1.13
**O_2**/Fe/HAP	−4.67
**O_2**/Co/HAP	−4.25
**O_2**/Ni/HAP	−3.17
**O_2**/Cu/HAP	−3.04
**CH_4**/O+O/Fe/HAP	−0.17
**CH_4**/O+O/Co/HAP	−0.22
**CH_4**/O+O/Ni/HAP	−0.20
**CH_4**/O+O/Cu/HAP	−0.31
**CH_3OH**/O/Fe/HAP	−1.27
**CH_3OH**/O/Co/HAP	−1.18
**CH_3OH**/O/Ni/HAP	−1.30
**CH_3OH**/O/Cu/HAP	−1.42

S-7 Activation Energy Barriers

In this section, we detail the energy profiles predicted by the CI-NEB calculations for the reactions proposed to elucidate the direct oxidation of methane to methanol on the surface of the TM/HAP catalysts. The first stage corresponds to the formation of O/TM active sites obtained via oxygen dissociation:

$$O_2{}^* \xrightarrow[\text{TM/HAP}]{\text{TS}} O^* + O^* \ ,$$

where the O_2 molecule breaks apart forming two oxygen radicals, with and potential energy path passing through only one transition state TS. The data corresponding to this reaction are depicted in Figures S-28 – S-35.

For the formation of methanol, two steps are proposed:

$$CH_4{}^* + O^* \xrightarrow[\text{TM/HAP}]{\text{TS}_1} CH_3{}^* + OH^* \,,$$
$$CH_3{}^* + OH^* \xrightarrow[\text{TM/HAP}]{\text{TS}_2} CH_3OH^* \,.$$

First, the CH_4 molecule is dehydrogenated by losing an hydrogen atom to an oxygen radical. This forms an intermediate state composed of a planar CH_3 oriented towards a OH radical adsorbed on the TM. Then, after a re-orientation between these two radicals, methanol is obtained. The data corresponding to these steps are depicted in Figures S-36 – S-51.

Finally, we investigate the stability of the CH_3OH molecule formed:

$$CH_3OH^* \xrightarrow[\text{TM/HAP}]{\text{TS}_1} CH_3{}^*/\text{TM} + OH^* \,,$$
$$CH_3{}^*/\text{TM} \xrightarrow[\text{TM/HAP}]{\text{TS}_2} CH_3{}^*/\text{O} \,.$$

Thus, the CH_3OH molecule is decomposed into CH_3 and OH radicals, both adsorbed on the TM-SAC. Then, the CH_3 radical diffuses to an oxygen site to reach a more stable configuration. The data corresponding to these steps are depicted in Figures S-52 – S-67. The light blue, pink, and red spheres represent Ca, P, and O atoms in the substrate, respectively; brown and white spheres indicate C and H atoms; light green spheres represent O atoms from the O_2 molecule; and ocher, dark blue, silver, and dark green spheres represent Fe, Co, Ni, and Cu transition metal SACs, respectively.

S-7.1 Reaction Path for Oxygen Dissociation

S-7.1.1 $O_2^* \xrightarrow[\text{Fe/HAP}]{\text{TS}} O^* + O^*$

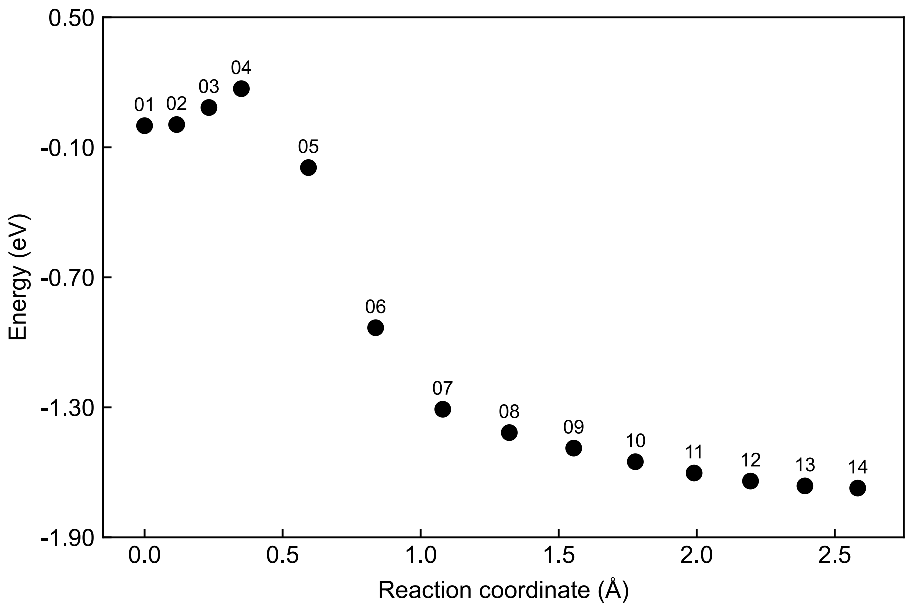

Figure S-28: Energy profile predicted by CI-NEB for the dissociation of O_2 on a Fe-SAC supported on the HAP(0001) surface. The top and side views of each CI-NEB image is shown in Figure S-29, as indicated by their respective labels (numbers above markers).

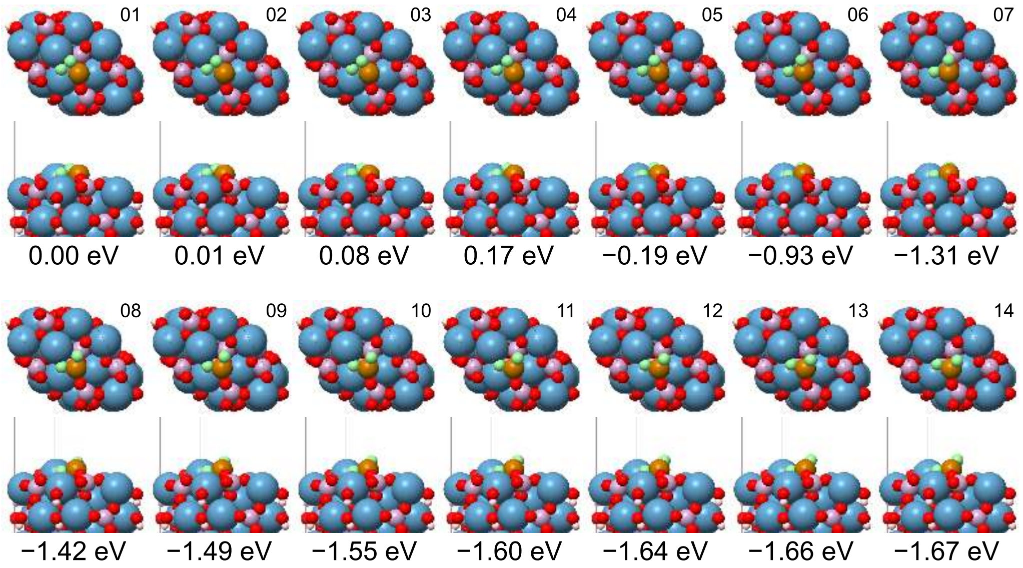

Figure S-29: Top and side views of each CI-NEB image predicted for the dissociation of O_2 on a Fe-SAC supported on the HAP(0001) surface. The CI-NEB images are ordered according to their labels (numbers at upper-right corners) defined in Figure S-28. Numbers below each structure indicate the relative energy with respect to the first CI-NEB image.

S-7.1.2 $O_2^* \xrightarrow[\text{Co/HAP}]{\text{TS}} O^* + O^*$

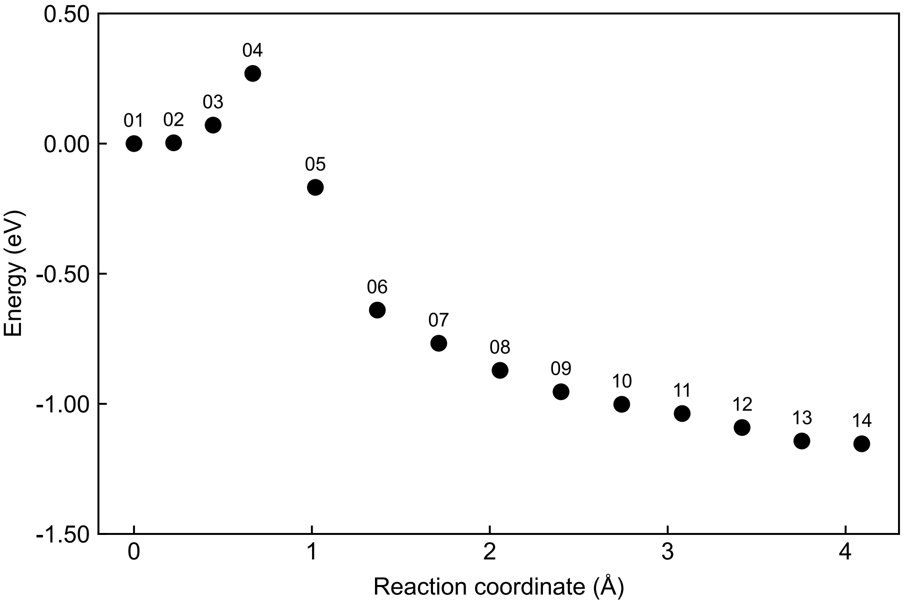

Figure S-30: Energy profile predicted by CI-NEB for the dissociation of O_2 on a Co-SAC supported on the HAP(0001) surface. The top and side views of each CI-NEB image is shown in Figure S-31, as indicated by their respective labels (numbers above markers).

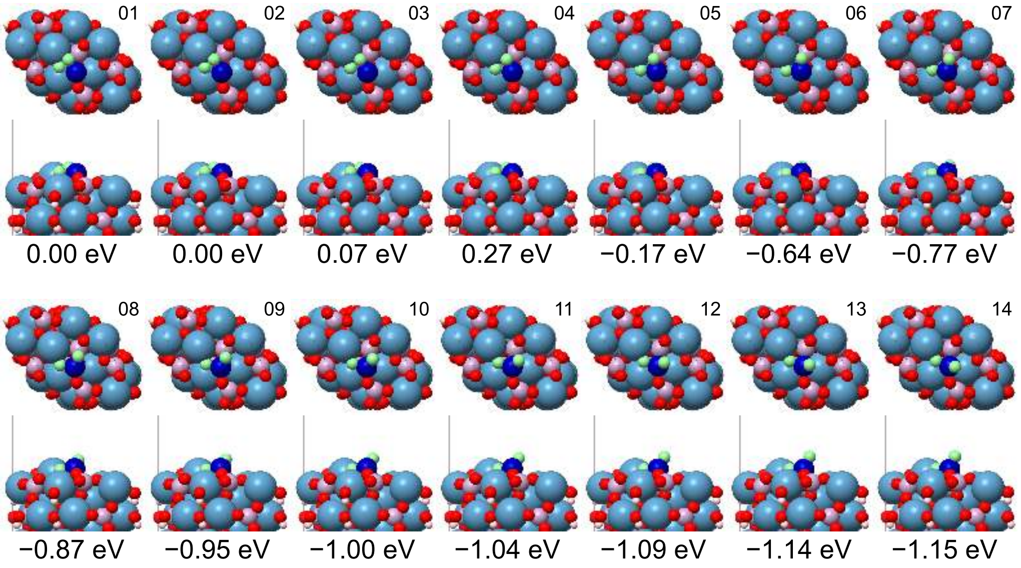

Figure S-31: Top and side views of each CI-NEB image predicted for the dissociation of O_2 on a Co-SAC supported on the HAP(0001) surface. The CI-NEB images are ordered according to their labels (numbers at upper-right corners) defined in Figure S-30. Numbers below each structure indicate the relative energy with respect to the first CI-NEB image.

S-7.1.3 $O_2^* \xrightarrow[\text{Ni/HAP}]{\text{TS}} O^* + O^*$

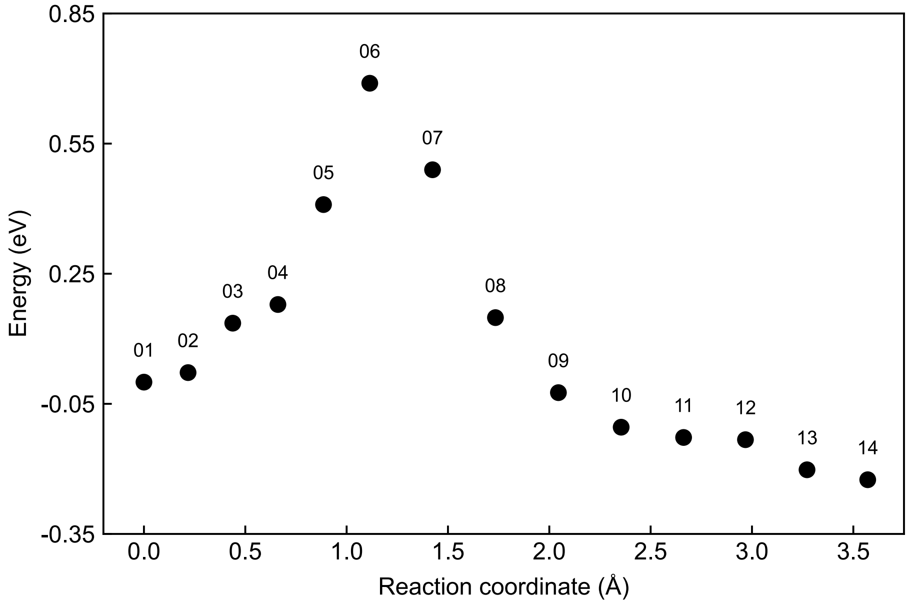

Figure S-32: Energy profile predicted by CI-NEB for the dissociation of O_2 on a Ni-SAC supported on the HAP(0001) surface. The top and side views of each CI-NEB image is shown in Figure S-33, as indicated by their respective labels (numbers above markers).

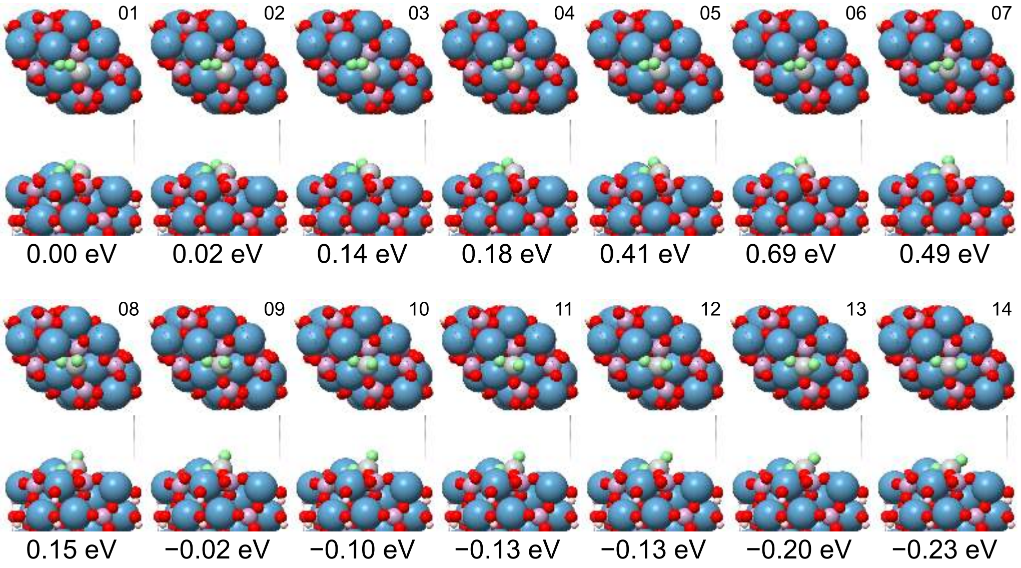

Figure S-33: Top and side views of each CI-NEB image predicted for the dissociation of O_2 on a Ni-SAC supported on the HAP(0001) surface. The CI-NEB images are ordered according to their labels (numbers at upper-right corners) defined in Figure S-32. Numbers below each structure indicate the relative energy with respect to the first CI-NEB image.

S-7.1.4 $O_2{}^* \xrightarrow[\text{Cu/HAP}]{\text{TS}} O^* + O^*$

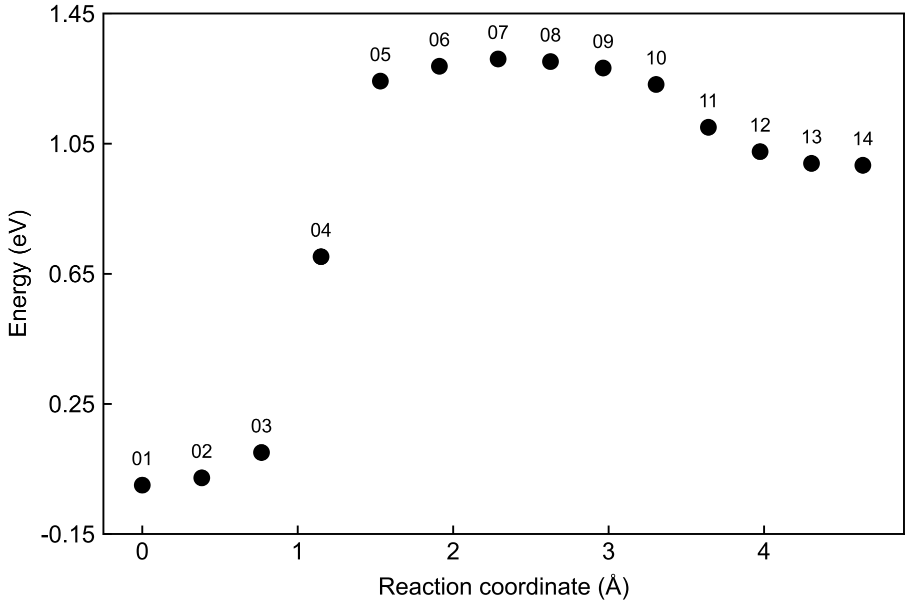

Figure S-34: Energy profile predicted by CI-NEB for the dissociation of O_2 on a Cu-SAC supported on the HAP(0001) surface. The top and side views of each CI-NEB image is shown in Figure S-35, as indicated by their respective labels (numbers above markers).

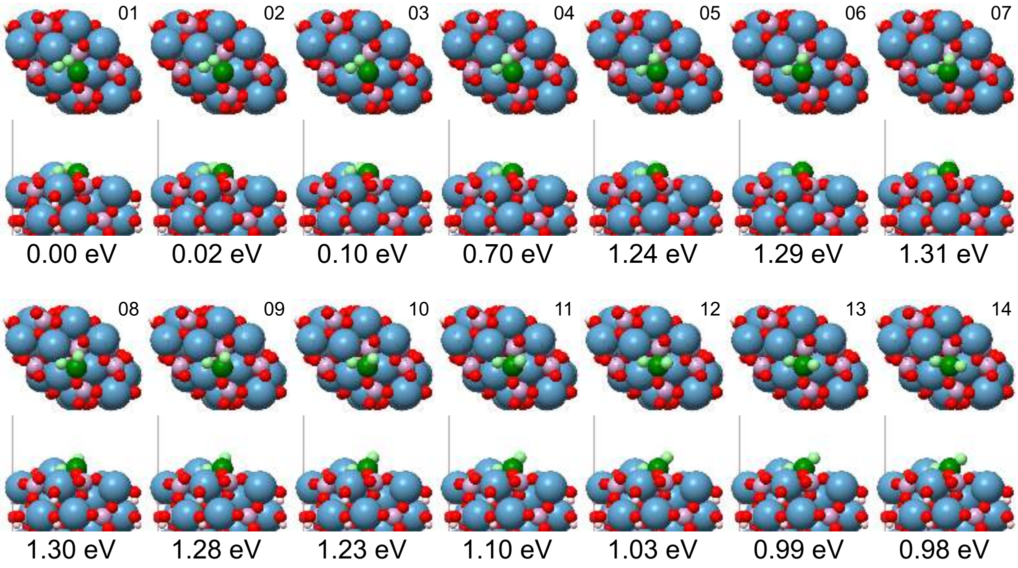

Figure S-35: Top and side views of each CI-NEB image predicted for the dissociation of O_2 on a Cu-SAC supported on the HAP(0001) surface. The CI-NEB images are ordered according to their labels (numbers at upper-right corners) defined in Figure S-34. Numbers below each structure indicate the relative energy with respect to the first CI-NEB image.

S-7.2 Reaction Path for C–H Bond Cleavage

S-7.2.1 $CH_4^* + O^* \xrightarrow[\text{Fe/HAP}]{\text{TS}_1} CH_3^* + OH^*$

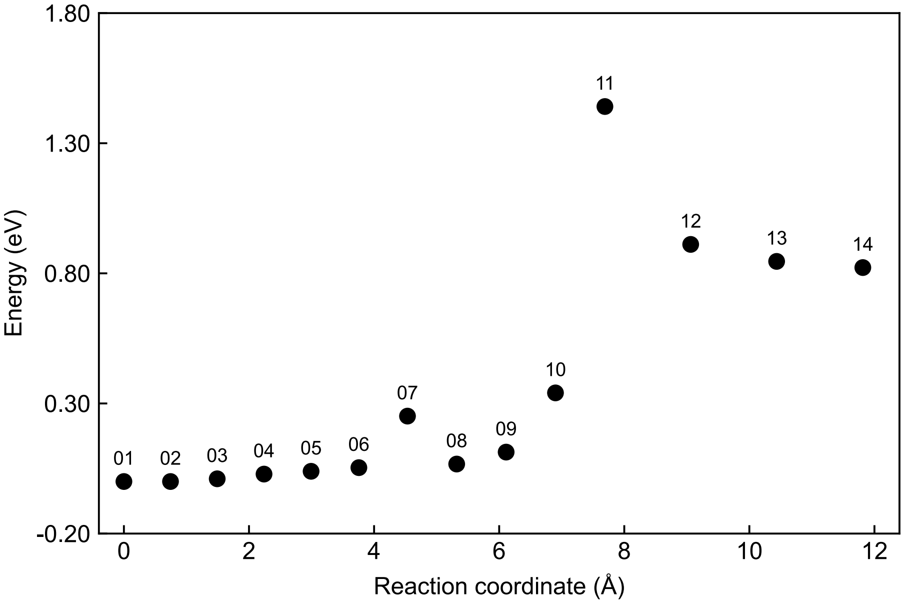

Figure S-36: Energy profile predicted by CI-NEB for the first dehydrogenation of CH_4 on a Fe-SAC supported on the HAP(0001) surface. The top and side views of each CI-NEB image is shown in Figure S-37, as indicated by their respective labels (numbers above markers).

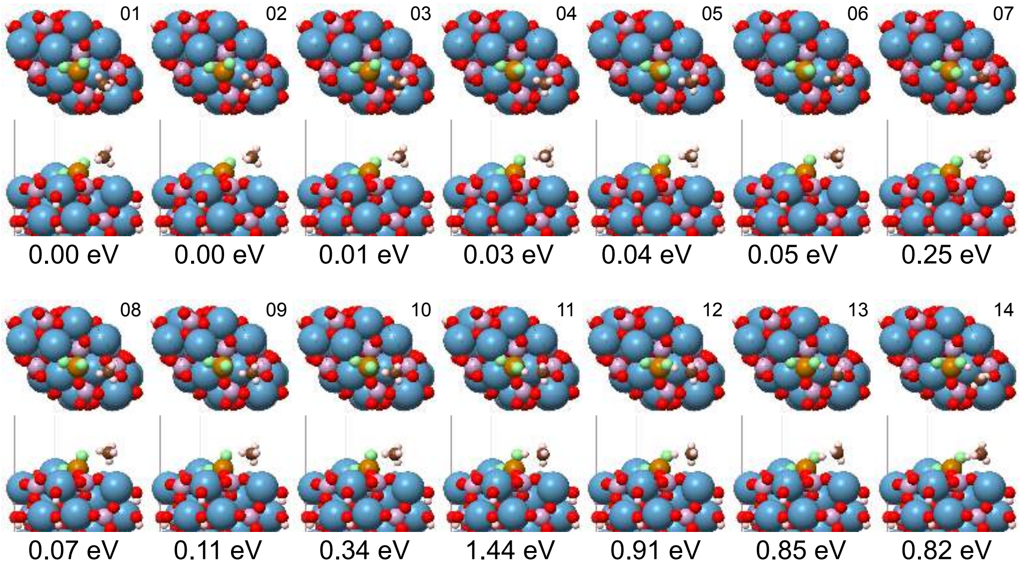

Figure S-37: Top and side views of each CI-NEB image predicted for the first dehydrogenation of CH_4 on a Fe-SAC supported on the HAP(0001) surface. The CI-NEB images are ordered according to their labels (numbers at upper-right corners) defined in Figure S-36. Numbers below each structure indicate the relative energy with respect to the first CI-NEB image.

S-7.2.2 $CH_4^* + O^* \xrightarrow[\text{Co/HAP}]{\text{TS}_1} CH_3^* + OH^*$

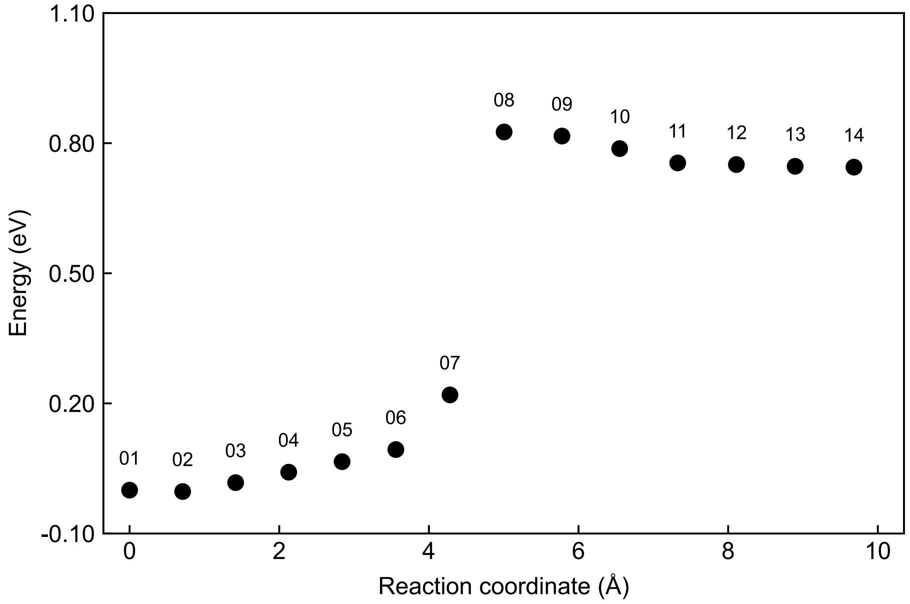

Figure S-38: Energy profile predicted by CI-NEB for the first dehydrogenation of CH_4 on a Co-SAC supported on the HAP(0001) surface. The top and side views of each CI-NEB image is shown in Figure S-39, as indicated by their respective labels (numbers above markers).

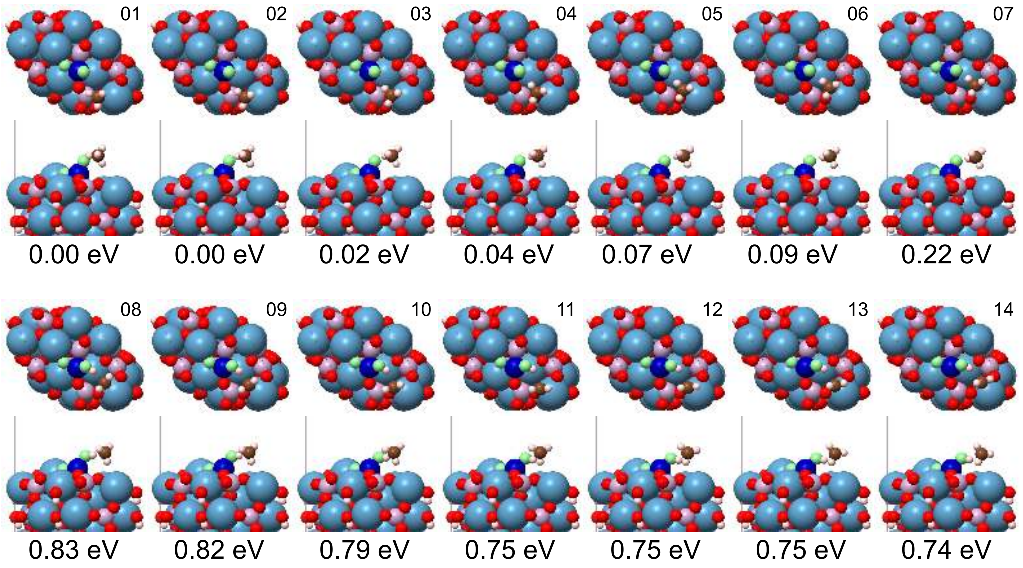

Figure S-39: Top and side views of each CI-NEB image predicted for the first dehydrogenation of CH_4 on a Co-SAC supported on the HAP(0001) surface. The CI-NEB images are ordered according to their labels (numbers at upper-right corners) defined in Figure S-38. Numbers below each structure indicate the relative energy with respect to the first CI-NEB image.

S-7.2.3 $CH_4^* + O^* \xrightarrow[\text{Ni/HAP}]{\text{TS}_1} CH_3^* + OH^*$

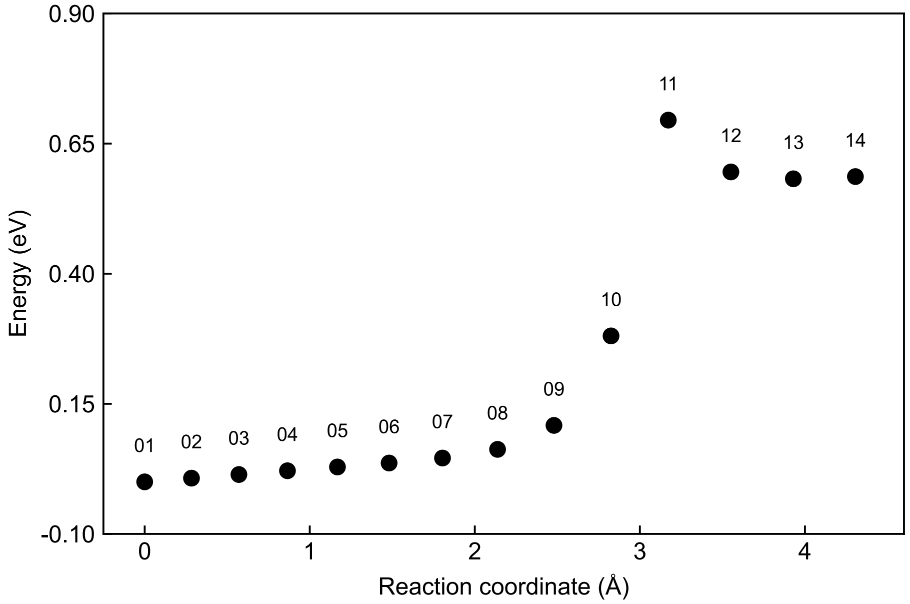

Figure S-40: Energy profile predicted by CI-NEB for the first dehydrogenation of CH_4 on a Ni-SAC supported on the HAP(0001) surface. The top and side views of each CI-NEB image is shown in Figure S-41, as indicated by their respective labels (numbers above markers).

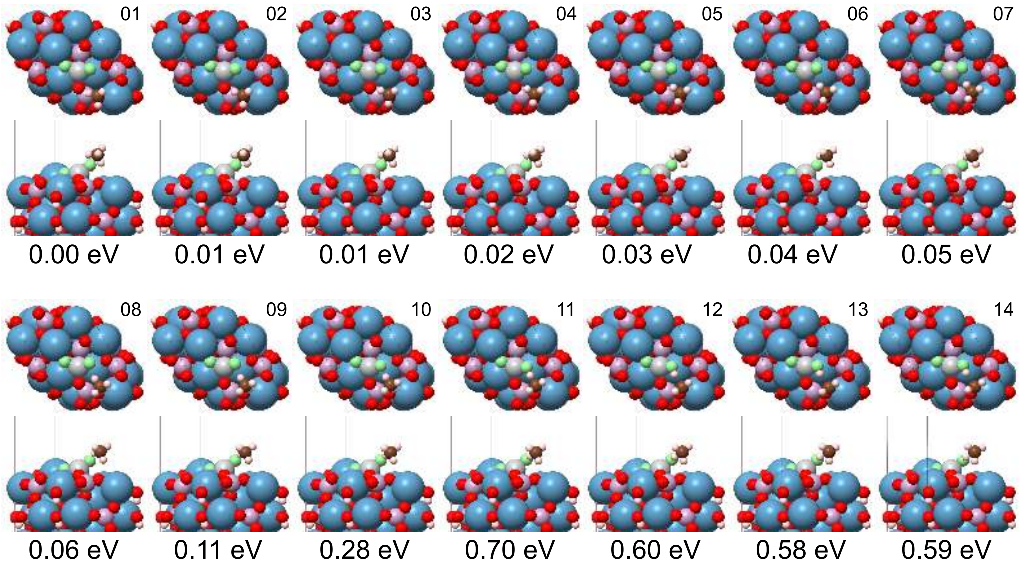

Figure S-41: Top and side views of each CI-NEB image predicted for the first dehydrogenation of CH_4 on a Ni-SAC supported on the HAP(0001) surface. The CI-NEB images are ordered according to their labels (numbers at upper-right corners) defined in Figure S-40. Numbers below each structure indicate the relative energy with respect to the first CI-NEB image.

S-7.2.4 $CH_4^* + O^* \xrightarrow[\text{Cu/HAP}]{\text{TS}_1} CH_3^* + OH^*$

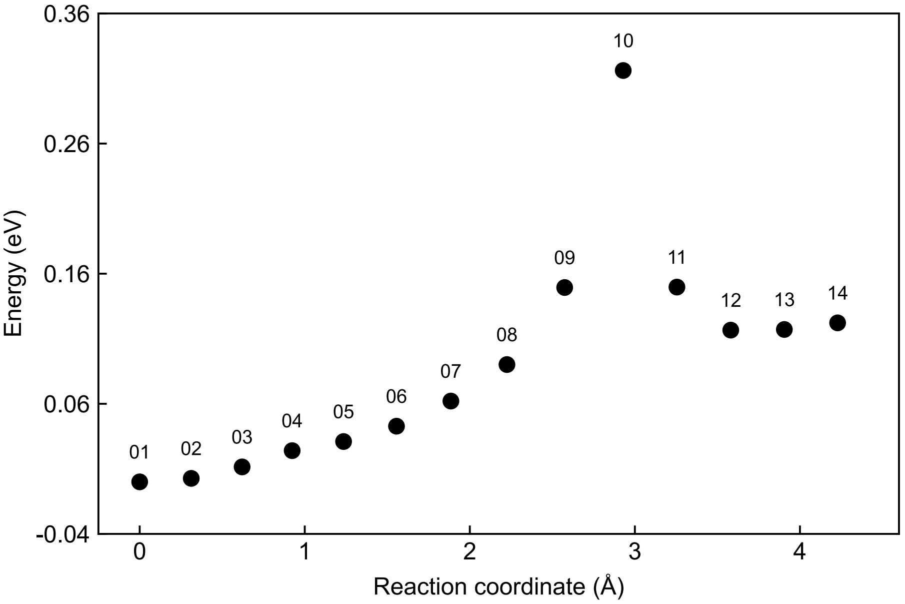

Figure S-42: Energy profile predicted by CI-NEB for the first dehydrogenation of CH_4 on a Cu-SAC supported on the HAP(0001) surface. The top and side views of each CI-NEB image is shown in Figure S-43, as indicated by their respective labels (numbers above markers).

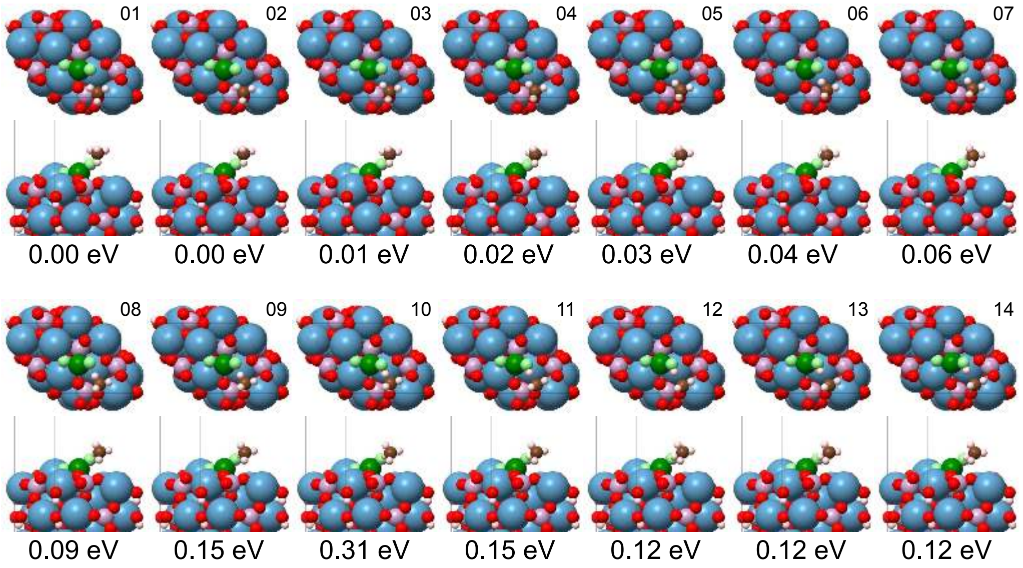

Figure S-43: Top and side views of each CI-NEB image predicted for the first dehydrogenation of CH_4 on a Cu-SAC supported on the HAP(0001) surface. The CI-NEB images are ordered according to their labels (numbers at upper-right corners) defined in Figure S-42. Numbers below each structure indicate the relative energy with respect to the first CI-NEB image.

S-7.3 Reaction Path for Methanol Formation

S-7.3.1 $CH_3^* + OH^* \xrightarrow[\text{Fe/HAP}]{TS_2} CH_3OH^*$

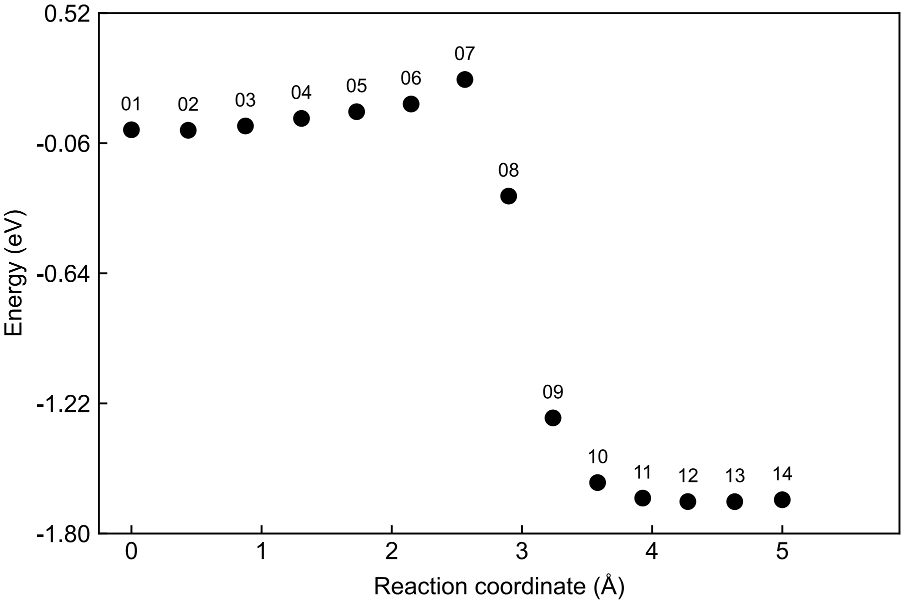

Figure S-44: Energy profile predicted by CI-NEB for the methanol formation on a Fe-SAC supported on the HAP(0001) surface. The top and side views of each CI-NEB image is shown in Figure S-45, as indicated by their respective labels (numbers above markers).

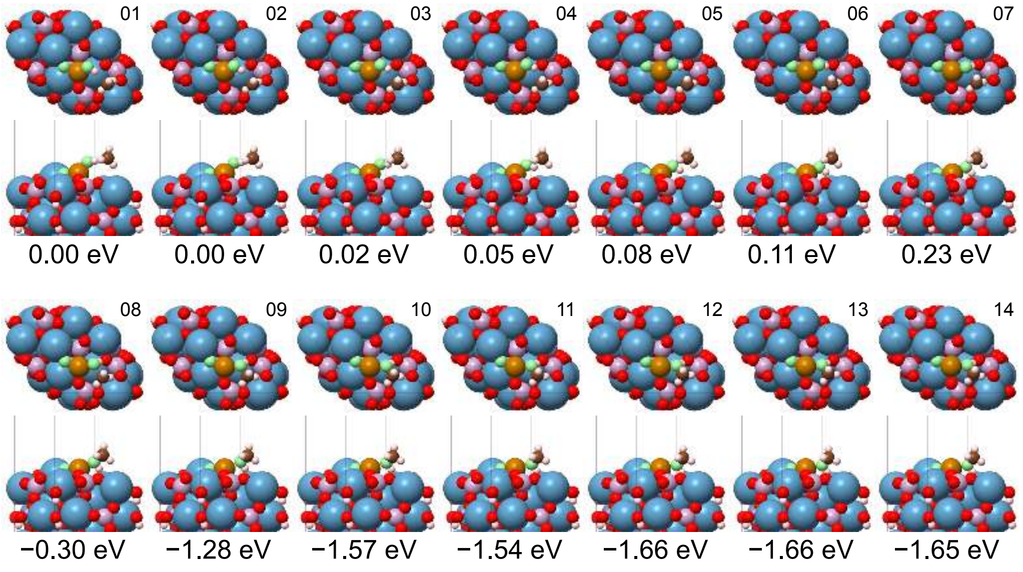

Figure S-45: Top and side views of each CI-NEB image predicted for the methanol formation on a Fe-SAC supported on the HAP(0001) surface. The CI-NEB images are ordered according to their labels (numbers at upper-right corners) defined in Figure S-44. Numbers below each structure indicate the relative energy with respect to the first CI-NEB image.

S-7.3.2 $CH_3^* + OH^* \xrightarrow[\text{Co/HAP}]{\text{TS}_2} CH_3OH^*$

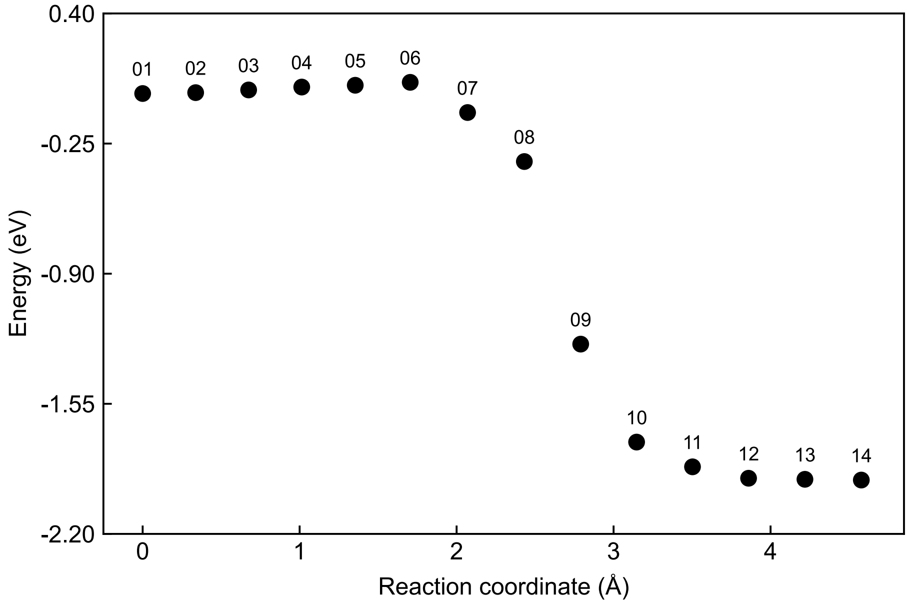

Figure S-46: Energy profile predicted by CI-NEB for the methanol formation on a Co-SAC supported on the HAP(0001) surface. The top and side views of each CI-NEB image is shown in Figure S-47, as indicated by their respective labels (numbers above markers).

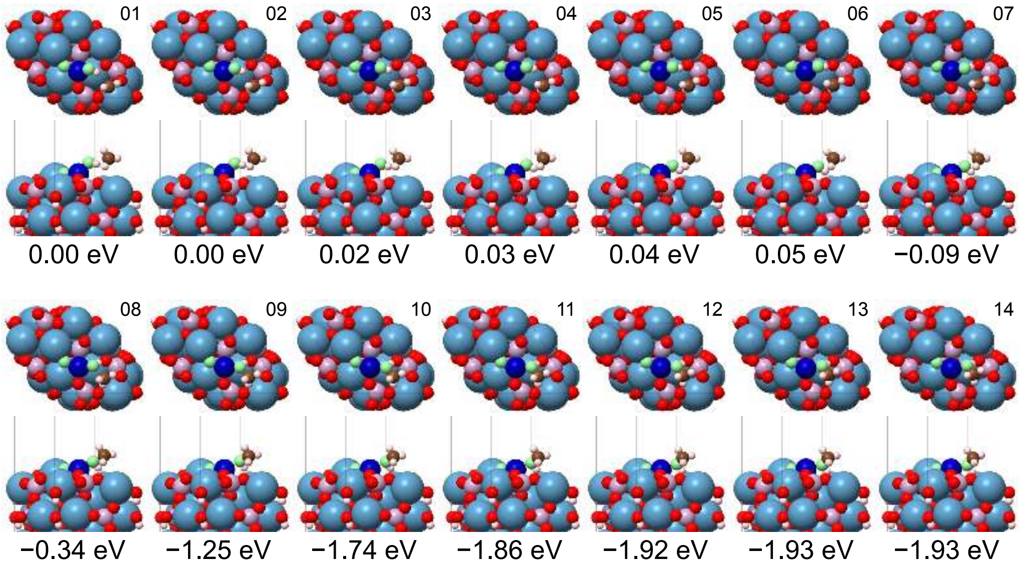

Figure S-47: Top and side views of each CI-NEB image predicted for the methanol formation on a Co-SAC supported on the HAP(0001) surface. The CI-NEB images are ordered according to their labels (numbers at upper-right corners) defined in Figure S-46. Numbers below each structure indicate the relative energy with respect to the first CI-NEB image.

S-7.3.3 $CH_3^* + OH^* \xrightarrow[\text{Ni/HAP}]{\text{TS}_2} CH_3OH^*$

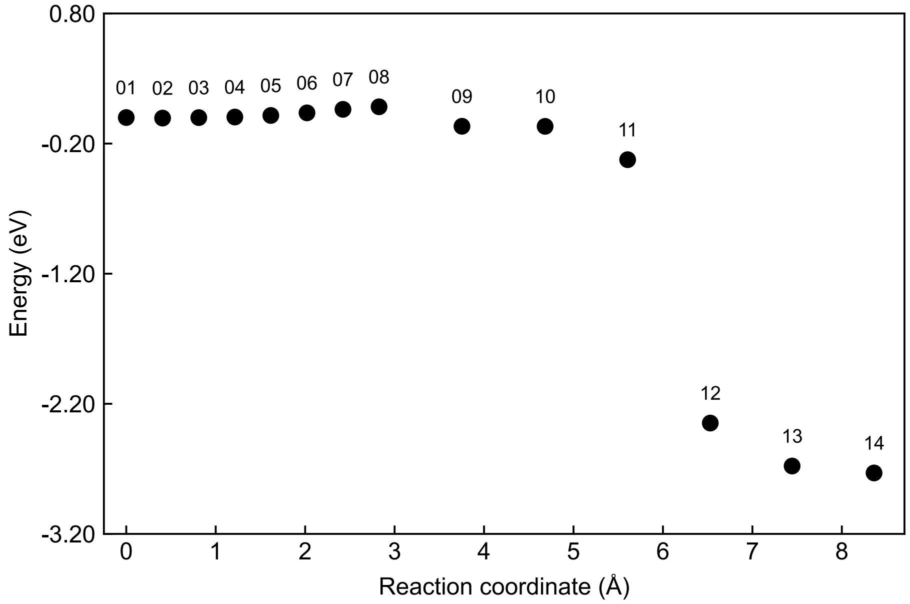

Figure S-48: Energy profile predicted by CI-NEB for the methanol formation on a Ni-SAC supported on the HAP(0001) surface. The top and side views of each CI-NEB image is shown in Figure S-49, as indicated by their respective labels (numbers above markers).

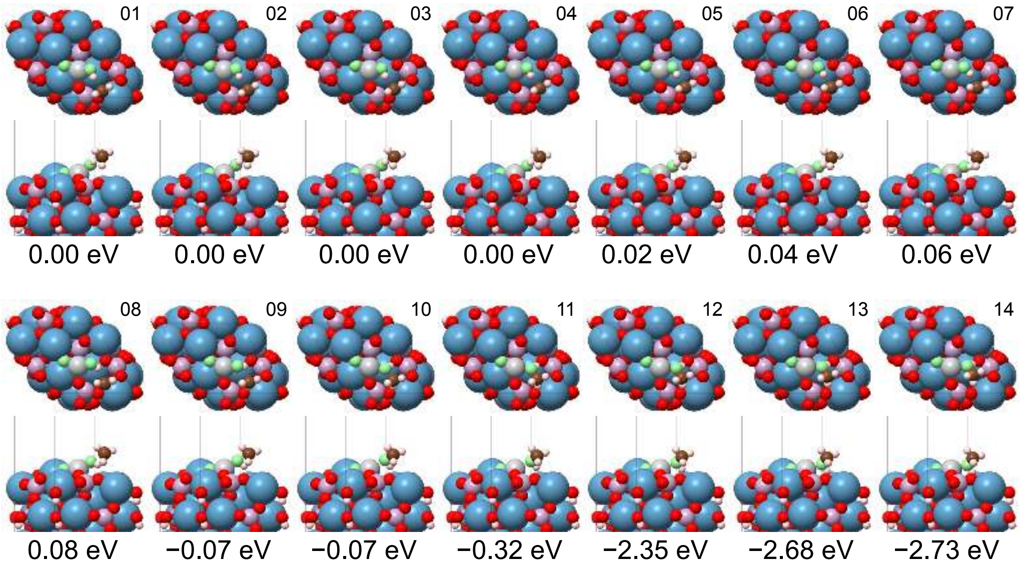

Figure S-49: Top and side views of each CI-NEB image predicted for the methanol formation on a Ni-SAC supported on the HAP(0001) surface. The CI-NEB images are ordered according to their labels (numbers at upper-right corners) defined in Figure S-48. Numbers below each structure indicate the relative energy with respect to the first CI-NEB image.

S-7.3.4 $CH_3^* + OH^* \xrightarrow[\text{Cu/HAP}]{\text{TS}_2} CH_3OH^*$

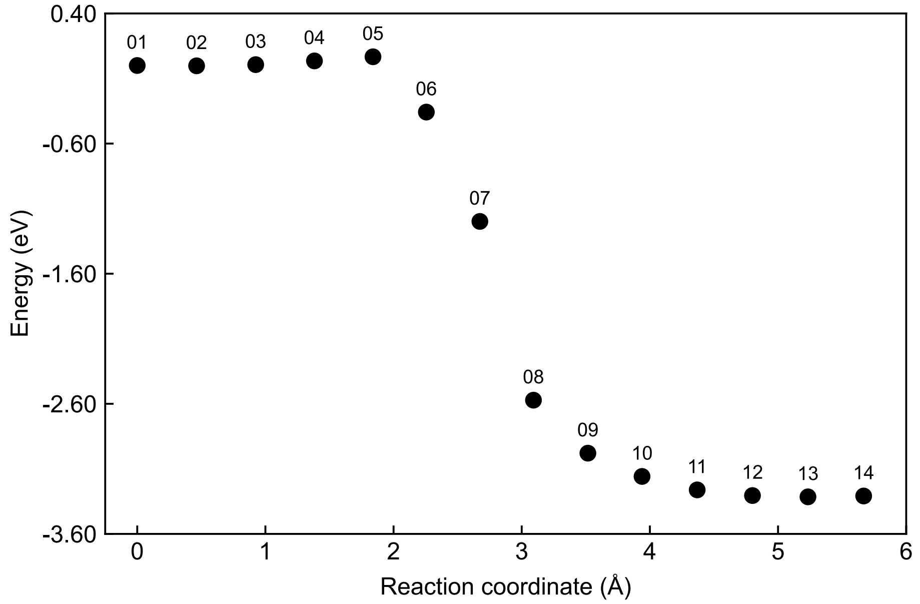

Figure S-50: Energy profile predicted by CI-NEB for the methanol formation on a Cu-SAC supported on the HAP(0001) surface. The top and side views of each CI-NEB image is shown in Figure S-51, as indicated by their respective labels (numbers above markers).

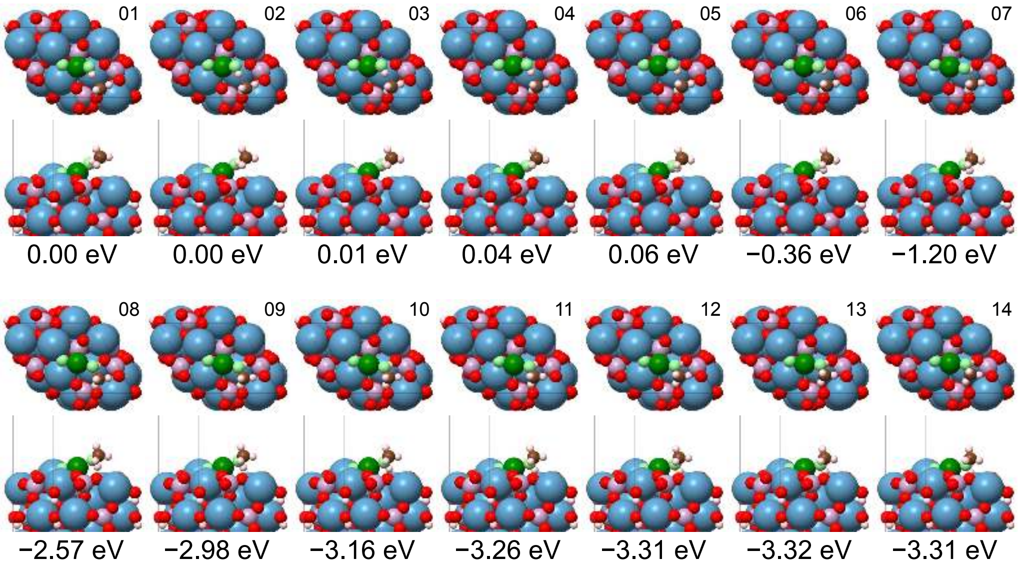

Figure S-51: Top and side views of each CI-NEB image predicted for the methanol formation on a Cu-SAC supported on the HAP(0001) surface. The CI-NEB images are ordered according to their labels (numbers at upper-right corners) defined in Figure S-50. Numbers below each structure indicate the relative energy with respect to the first CI-NEB image.

S-7.4 Reaction Path for Methanol Dissociation

S-7.4.1 $CH_3OH^* \xrightarrow[\text{Fe/HAP}]{\text{TS}_1} CH_3^*/TM + OH^*$

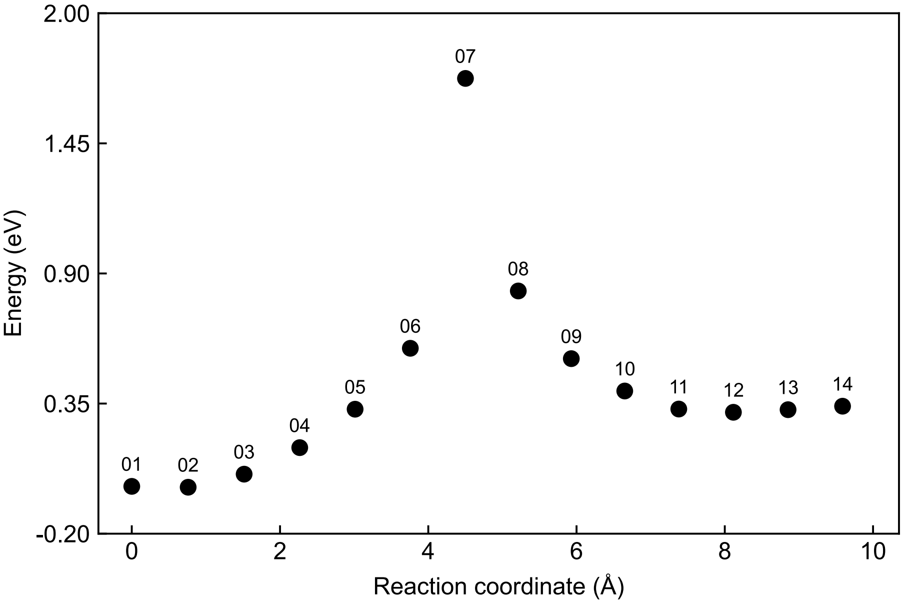

Figure S-52: Energy profile predicted by CI-NEB for the methanol decomposition on a Fe-SAC supported on the HAP(0001) surface. The top and side views of each CI-NEB image is shown in Figure S-53, as indicated by their respective labels (numbers above markers).

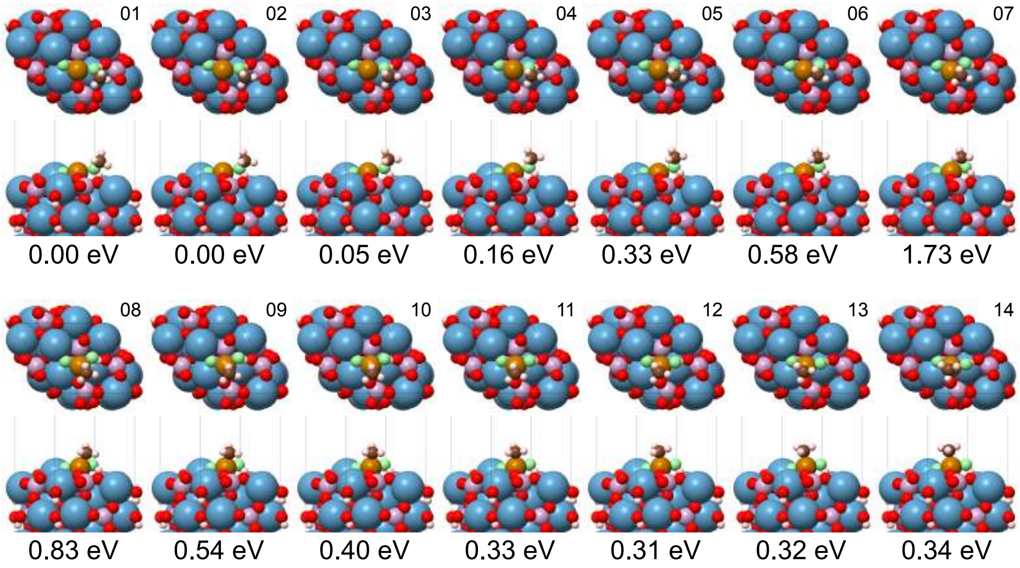

Figure S-53: Top and side views of each CI-NEB image predicted for the methanol decomposition on a Fe-SAC supported on the HAP(0001) surface. The CI-NEB images are ordered according to their labels (numbers at upper-right corners) defined in Figure S-52. Numbers below each structure indicate the relative energy with respect to the first CI-NEB image.

S-7.4.2 $CH_3OH^* \xrightarrow[\text{Co/HAP}]{TS_1} CH_3^*/TM + OH^*$

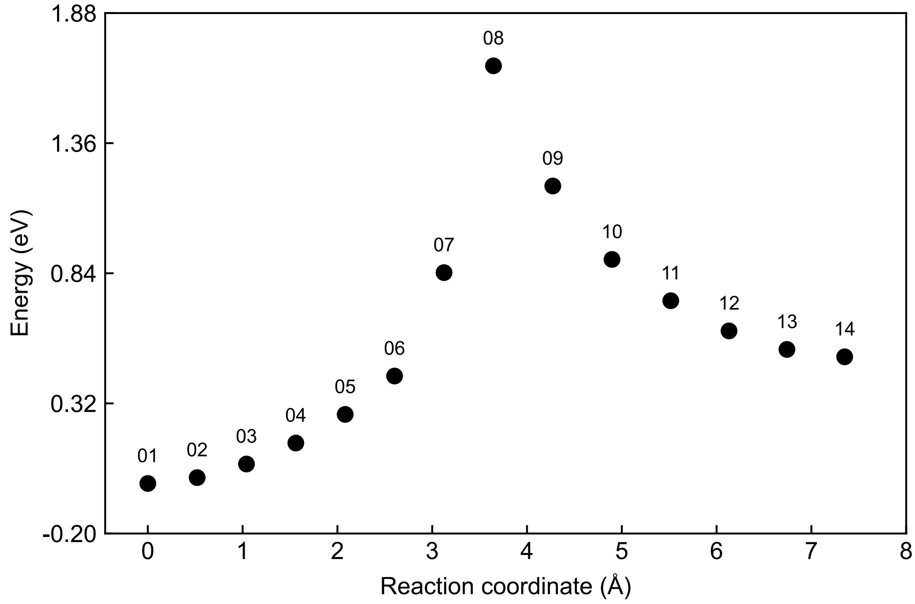

Figure S-54: Energy profile predicted by CI-NEB for the methanol decomposition on a Co-SAC supported on the HAP(0001) surface. The top and side views of each CI-NEB image is shown in Figure S-55, as indicated by their respective labels (numbers above markers).

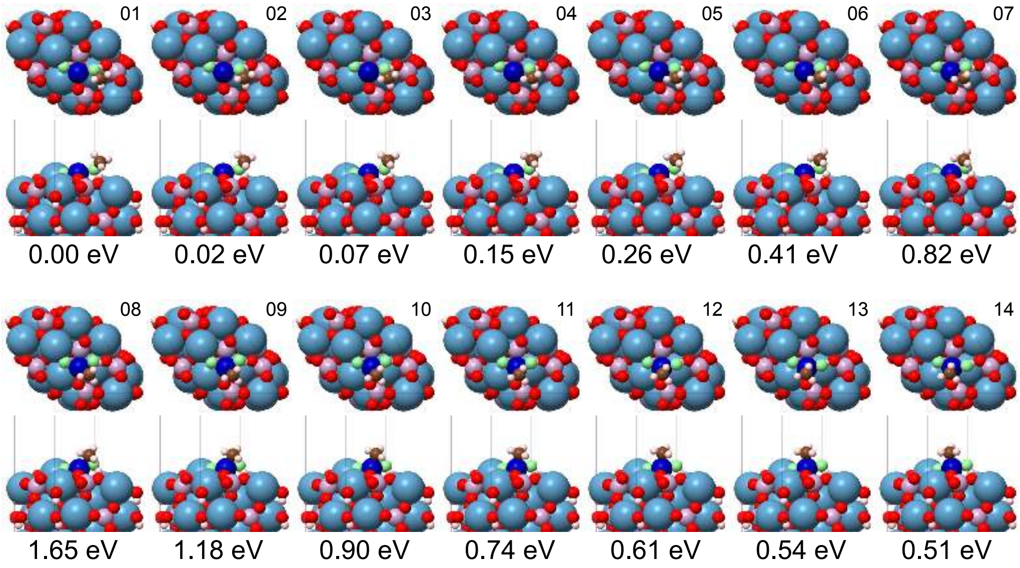

Figure S-55: Top and side views of each CI-NEB image predicted for the methanol decomposition on a Co-SAC supported on the HAP(0001) surface. The CI-NEB images are ordered according to their labels (numbers at upper-right corners) defined in Figure S-54. Numbers below each structure indicate the relative energy with respect to the first CI-NEB image.

S-7.4.3 $CH_3OH^* \xrightarrow[\text{Ni/HAP}]{\text{TS}_1} CH_3{}^*/TM + OH^*$

$CH_3OH^* \xrightarrow[\text{Ni/HAP}]{\text{TS}_1} CH_3{}^*/TM + OH^*$

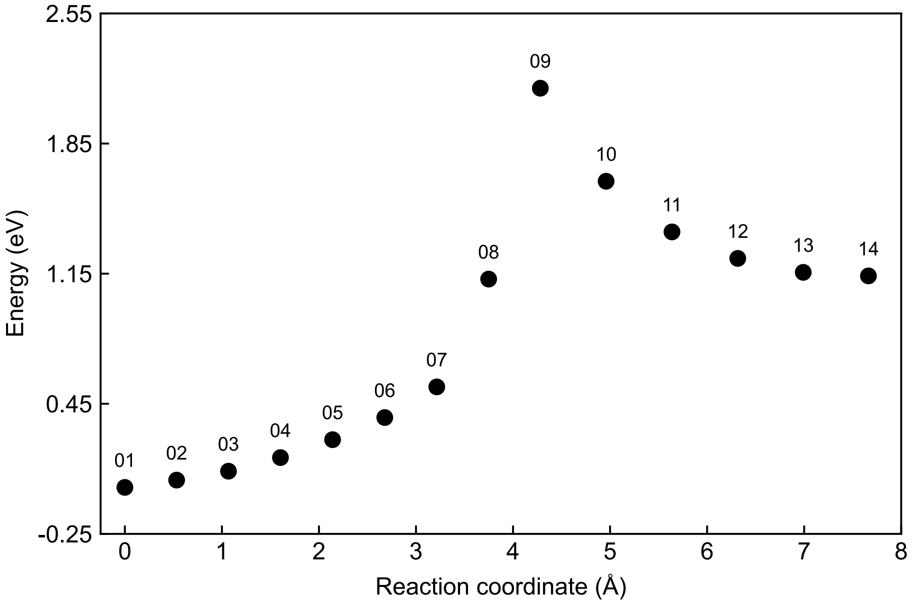

Figure S-56: Energy profile predicted by CI-NEB for the methanol decomposition on a Ni-SAC supported on the HAP(0001) surface. The top and side views of each CI-NEB image is shown in Figure S-57, as indicated by their respective labels (numbers above markers).

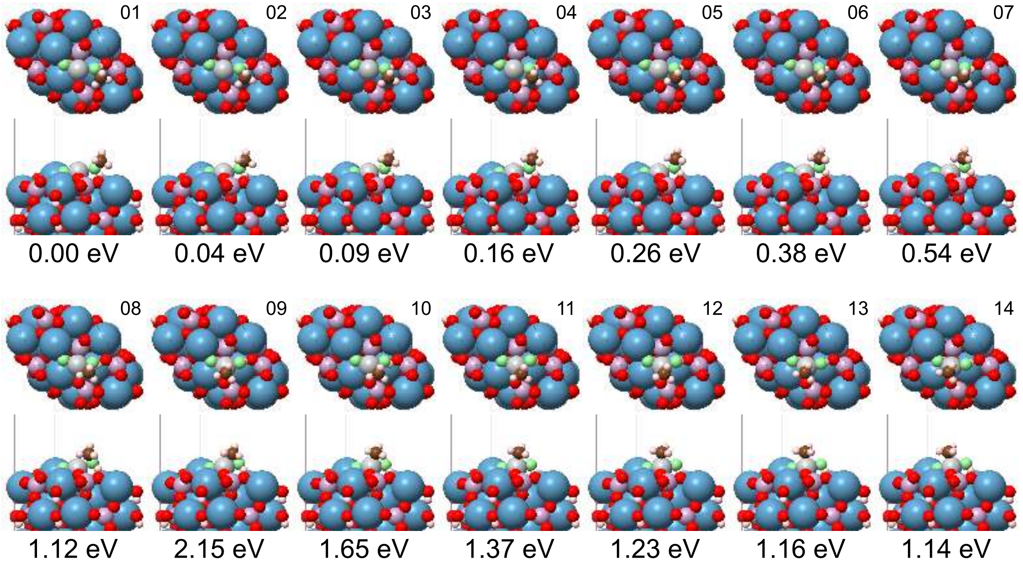

Figure S-57: Top and side views of each CI-NEB image predicted for the methanol decomposition on a Ni-SAC supported on the HAP(0001) surface. The CI-NEB images are ordered according to their labels (numbers at upper-right corners) defined in Figure S-56. Numbers below each structure indicate the relative energy with respect to the first CI-NEB image.

S-7.4.4 $CH_3OH^* \xrightarrow[\text{Cu/HAP}]{\text{TS}_1} CH_3^*/TM + OH^*$

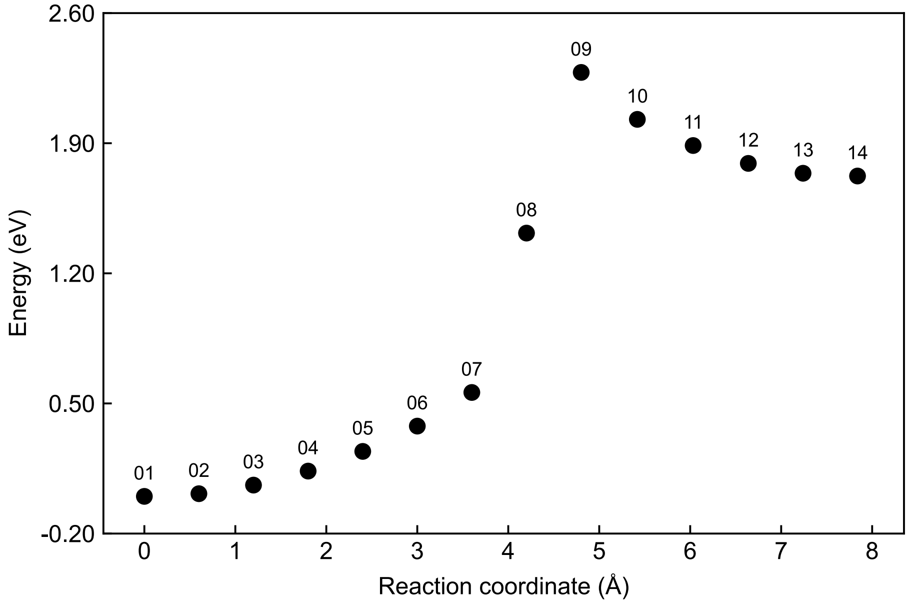

Figure S-58: Energy profile predicted by CI-NEB for the methanol decomposition on a Cu-SAC supported on the HAP(0001) surface. The top and side views of each CI-NEB image is shown in Figure S-59, as indicated by their respective labels (numbers above markers).

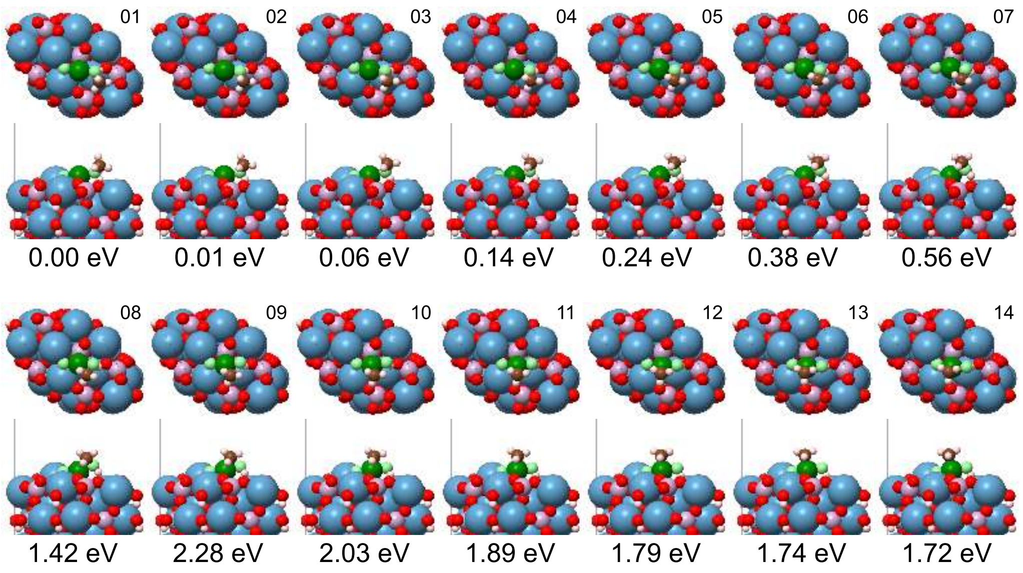

Figure S-59: Top and side views of each CI-NEB image predicted for the methanol decomposition on a Cu-SAC supported on the HAP(0001) surface. The CI-NEB images are ordered according to their labels (numbers at upper-right corners) defined in Figure S-58. Numbers below each structure indicate the relative energy with respect to the first CI-NEB image.

S-7.5 Reaction Path for Diffusion of the CH_3 Fragment

S-7.5.1 CH_3*/TM $\xrightarrow[\text{Fe/HAP}]{\text{TS}_2}$ CH_3*/O

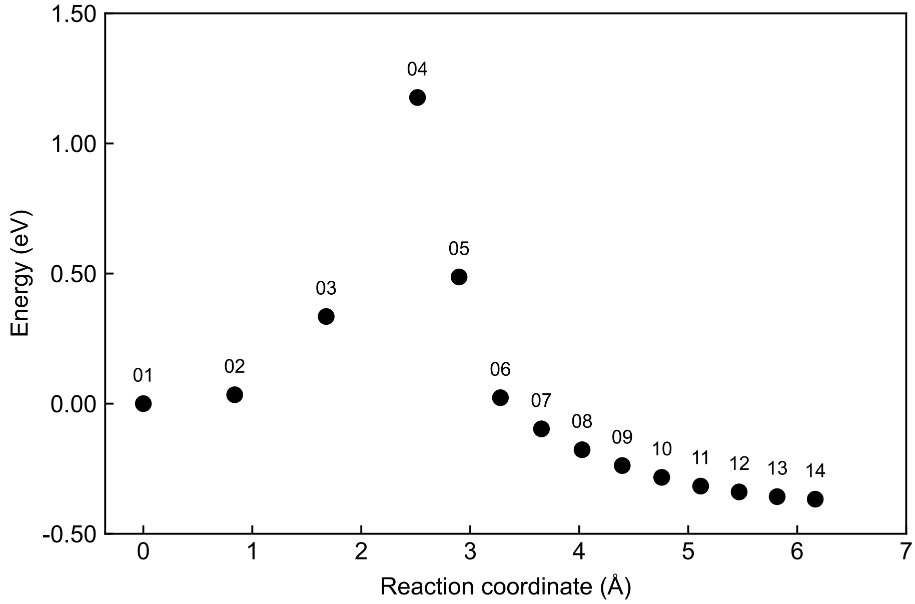

Figure S-60: Energy profile predicted by CI-NEB for the migration of the methyl group on a Fe-SAC supported on the HAP(0001) surface. The top and side views of each CI-NEB image is shown in Figure S-61, as indicated by their respective labels (numbers above markers).

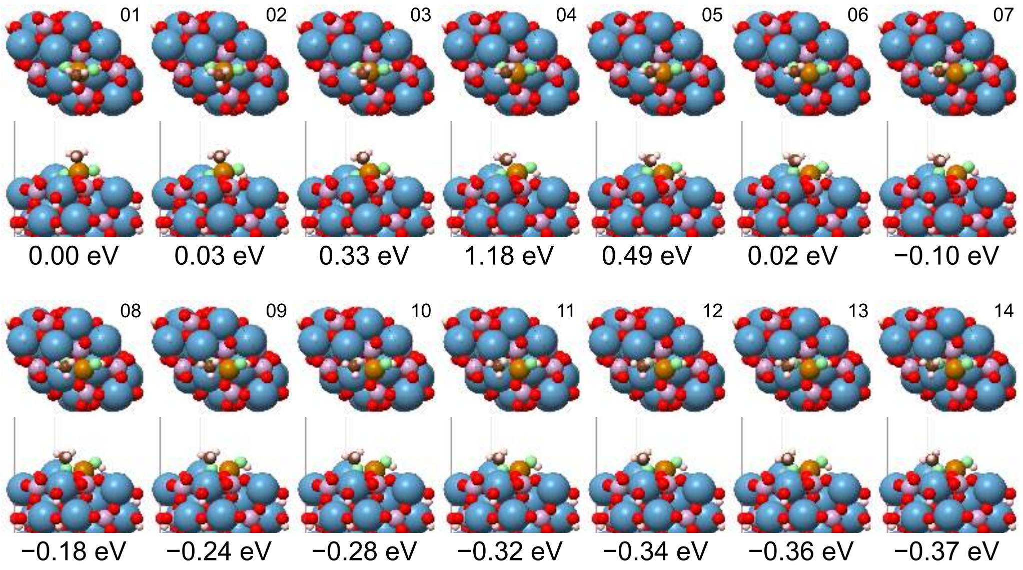

Figure S-61: Top and side views of each CI-NEB image predicted for the migration of the methyl group on a Fe-SAC supported on the HAP(0001) surface. The CI-NEB images are ordered according to their labels (numbers at upper-right corners) defined in Figure S-60. Numbers below each structure indicate the relative energy with respect to the first CI-NEB image.

S-7.5.2 $CH_3^*/TM \xrightarrow[\text{Co/HAP}]{TS_2} CH_3^*/O$

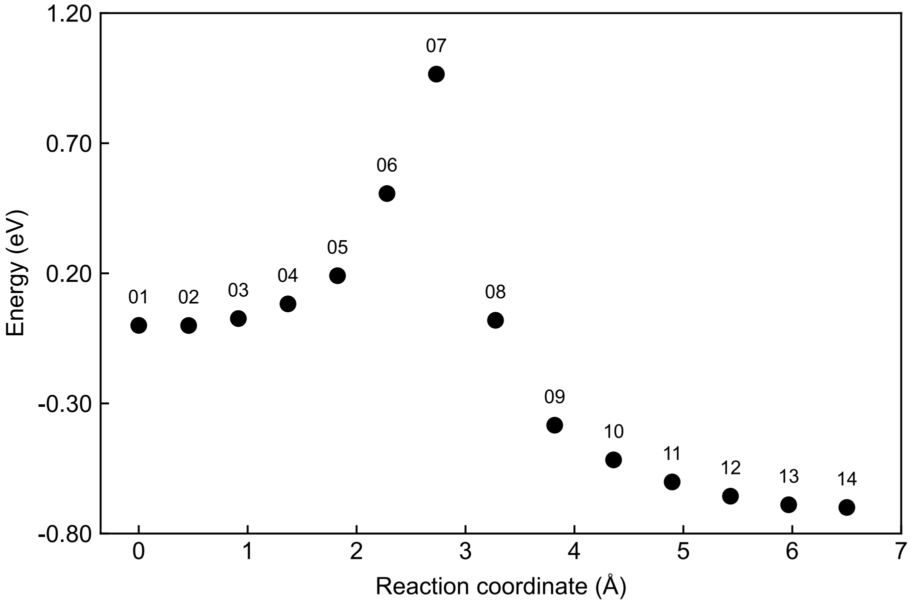

Figure S-62: Energy profile predicted by CI-NEB for the migration of the methyl group on a Co-SAC supported on the HAP(0001) surface. The top and side views of each CI-NEB image is shown in Figure S-63, as indicated by their respective labels (numbers above markers).

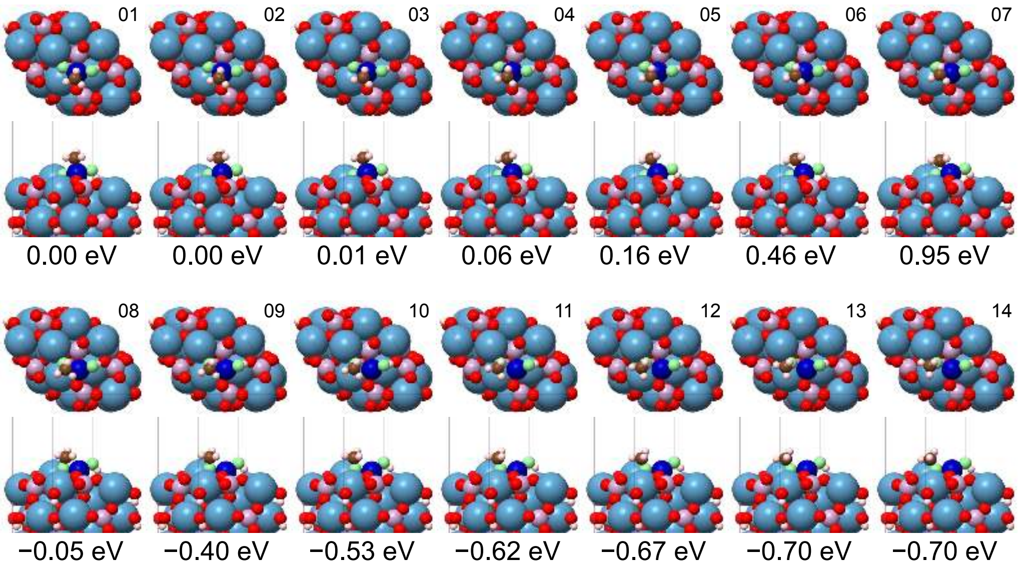

Figure S-63: Top and side views of each CI-NEB image predicted for the migration of the methyl group on a Co-SAC supported on the HAP(0001) surface. The CI-NEB images are ordered according to their labels (numbers at upper-right corners) defined in Figure S-62. Numbers below each structure indicate the relative energy with respect to the first CI-NEB image.

S-7.5.3 $CH_3^*/TM \xrightarrow[\text{Ni/HAP}]{\text{TS}_2} CH_3^*/O$

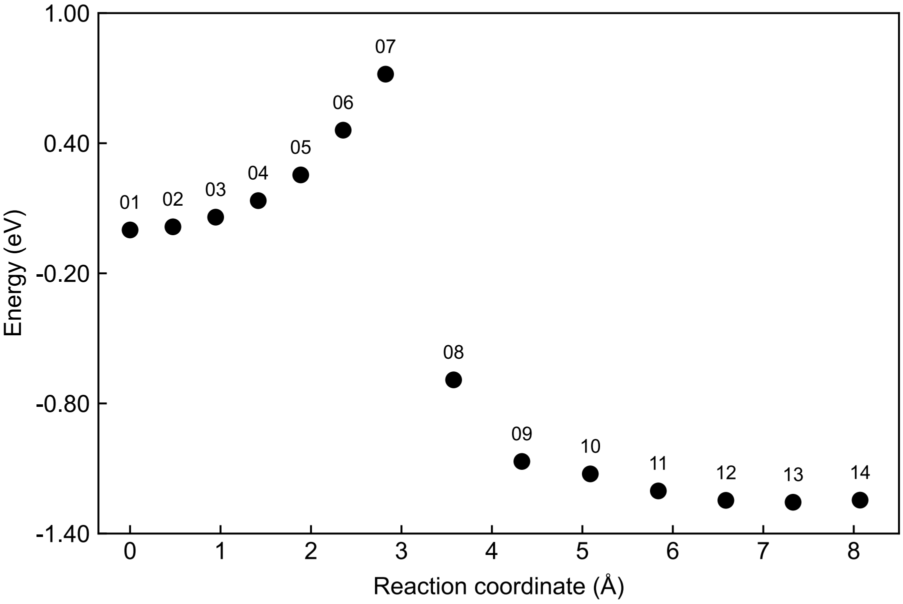

Figure S-64: Energy profile predicted by CI-NEB for the migration of the methyl group on a Ni-SAC supported on the HAP(0001) surface. The top and side views of each CI-NEB image is shown in Figure S-65, as indicated by their respective labels (numbers above markers).

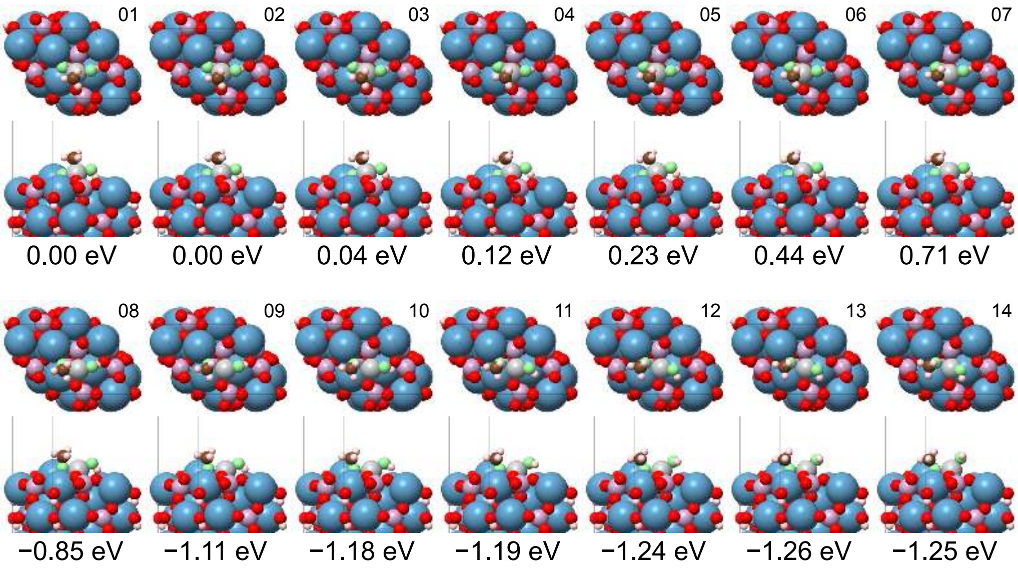

Figure S-65: Top and side views of each CI-NEB image predicted for the migration of the methyl group on a Ni-SAC supported on the HAP(0001) surface. The CI-NEB images are ordered according to their labels (numbers at upper-right corners) defined in Figure S-64. Numbers below each structure indicate the relative energy with respect to the first CI-NEB image.

S-7.5.4 $\mathrm{CH_3^*/TM} \xrightarrow[\mathrm{Cu/HAP}]{\mathrm{TS_2}} \mathrm{CH_3^*/O}$

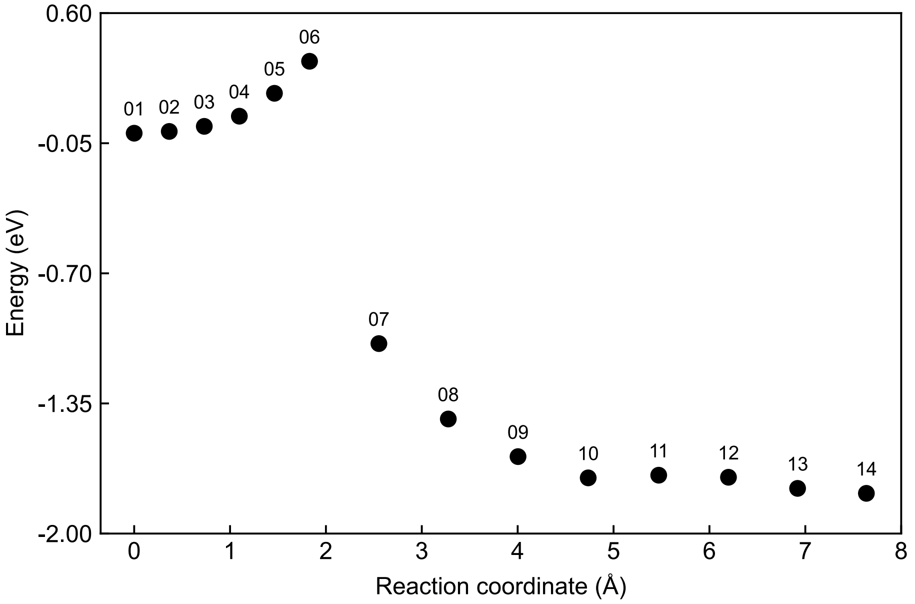

Figure S-66: Energy profile predicted by CI-NEB for the migration of the methyl group on a Cu-SAC supported on the HAP(0001) surface. The top and side views of each CI-NEB image is shown in Figure S-67, as indicated by their respective labels (numbers above markers).

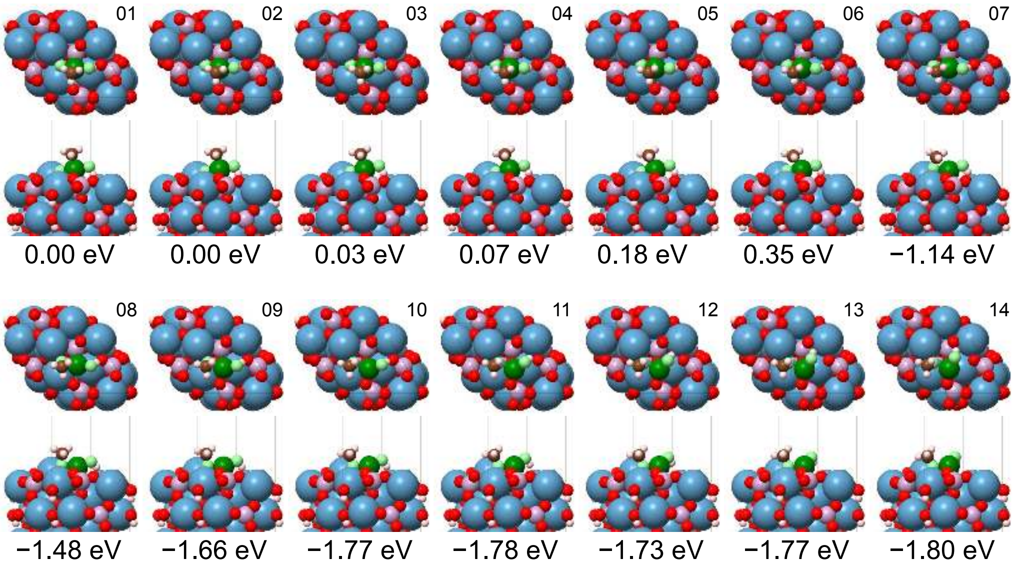

Figure S-67: Top and side views of each CI-NEB image predicted for the migration of the methyl group on a Cu-SAC supported on the HAP(0001) surface. The CI-NEB images are ordered according to their labels (numbers at upper-right corners) defined in Figure S-66. Numbers below each structure indicate the relative energy with respect to the first CI-NEB image.

S-8 Transition State Structures and Vibrational Frequencies

Table S-6: Views and vibrational frequencies of transition state structures located using the climbing image nudged elastic band method for each reaction system.

System	Structure	Vibrational frequencies (cm^{-1})
$O_2^* \xrightarrow[\text{Fe/HAP}]{\text{TS}} O^* + O^*$	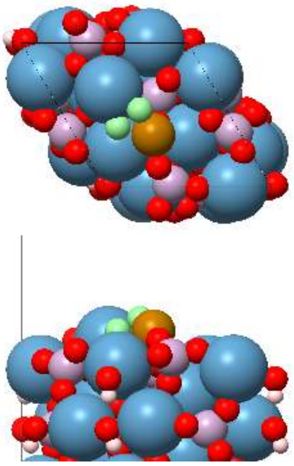	614.94, 614.94, 383.49, 260.50, 234.53, 162.20, −402.59
$O_2^* \xrightarrow[\text{Co/HAP}]{\text{TS}} O^* + O^*$	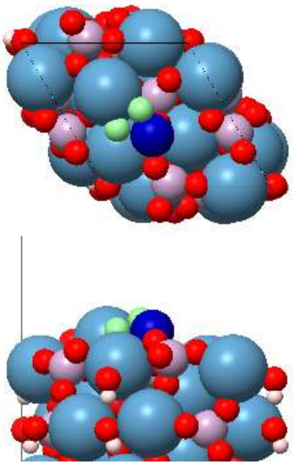	656.19, 379.23, 254.80, 229.18, 176.58, −330.02

Continued on next page

System	Structure	Vibrational frequencies (cm^{-1})
$O_2^* \xrightarrow[\text{Ni/HAP}]{\text{TS}} O^* + O^*$	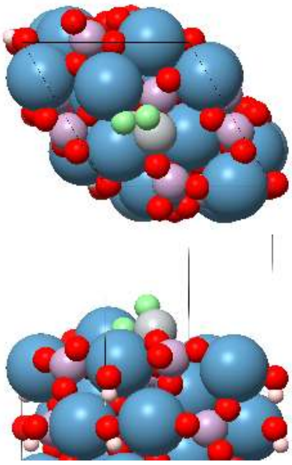	664.85, 448.70, 227.18, 185.17, 67.08, −310.48
$O_2^* \xrightarrow[\text{Cu/HAP}]{\text{TS}} O^* + O^*$	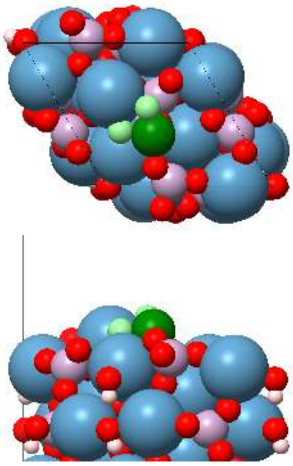	574.13, 447.09, 291.13, 235.45, 144.89, −160.77
$CH_4^* + O^* \xrightarrow[\text{Fe/HAP}]{\text{TS}_1} CH_3^* + OH^*$	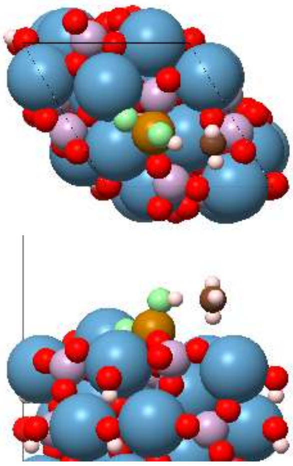	3399.69, 3244.10, 3243.41, 3061.64, 1364.55, 1356.77, 876.46, 649.68, 595.46, 364.86, 318.32, 226.66, 175.36, 118.42, 107.30, 82.54, 50.14, −18.39

Continued on next page

System	Structure	Vibrational frequencies (cm^{-1})
$CH_3^* + OH^* \xrightarrow[\text{Fe/HAP}]{TS_2} CH_3OH^*$	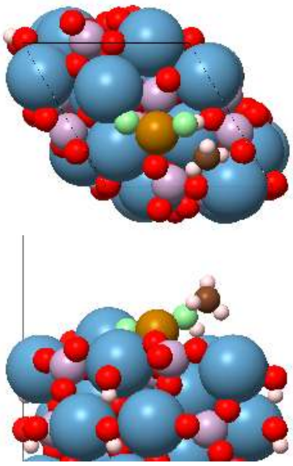	3463.99, 3259.42, 3247.56, 3074.20, 1368.98, 1349.22, 842.81, 717.36, 614.58, 471.01, 421.40, 370.91, 129.89, 99.77, 92.14, 76.33, 42.47, −300.38
$CH_4^* + O^* \xrightarrow[\text{Co/HAP}]{TS_1} CH_3^* + OH^*$	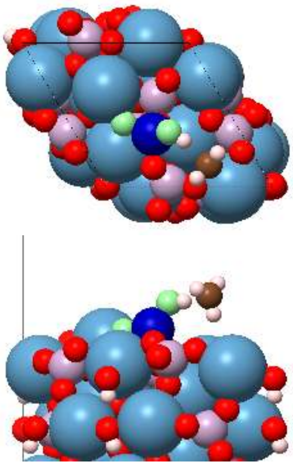	3355.16, 3247.88, 3238.34, 3069.53, 1366.49, 1354.32, 704.13, 663.19, 579.86, 435.89, 335.02, 269.20, 152.89, 109.14, 108.11, 89.85, 57.42, −37.14
$CH_3^* + OH^* \xrightarrow[\text{Co/HAP}]{TS_2} CH_3OH^*$	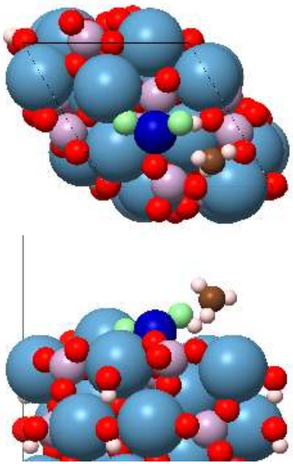	3548.57, 3240.77, 3236.42, 3055.83, 1369.12, 1349.62, 774.94, 645.78, 572.78, 446.45, 247.71, 194.78, 113.78, 108.26, 91.68, 77.96, 30.52, −35.26

Continued on next page

System	Structure	Vibrational frequencies (cm^{-1})
$CH_4{}^* + O^* \xrightarrow[\text{Ni/HAP}]{TS_1} CH_3{}^* + OH^*$	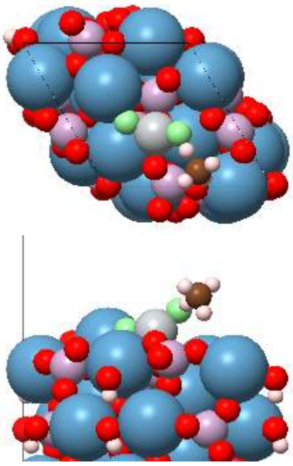	3185.51, 3177.12, 3027.05, 1385.17, 1371.36, 1126.45, 1089.09, 1007.22, 626.54, 528.42, 463.78, 416.41, 154.78, 131.42, 105.07, 67.16, 15.47, −1051.26
$CH_3{}^* + OH^* \xrightarrow[\text{Ni/HAP}]{TS_2} CH_3OH^*$	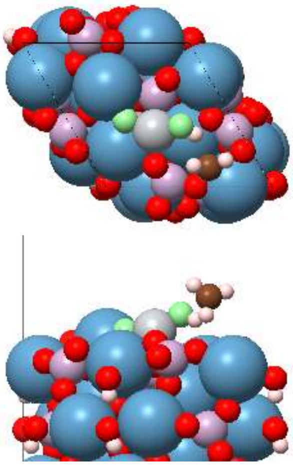	3622.89, 3245.03, 3231.67, 3048.88, 1380.34, 1360.71, 739.99, 659.83, 552.14, 347.21, 257.44, 248.66, 117.11, 115.20, 98.21, 78.50, 47.27, −89.42
$CH_4{}^* + O^* \xrightarrow[\text{Cu/HAP}]{TS_1} CH_3{}^* + OH^*$	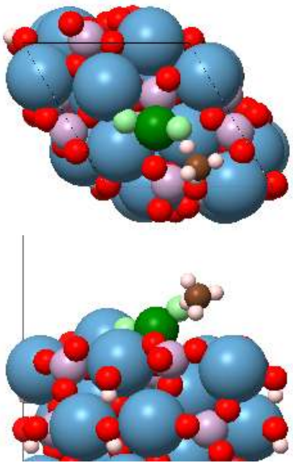	3162.91, 3155.58, 3005.11, 1386.36, 1362.42, 1135.54, 1107.60, 1072.51, 645.25, 522.49, 447.92, 409.34, 145.46, 107.75, 100.54, 75.96, 26.00, −1193.02

Continued on next page

System	Structure	Vibrational frequencies (cm^{-1})
$CH_3^* + OH^* \xrightarrow[\text{Cu/HAP}]{TS_2} CH_3OH^*$	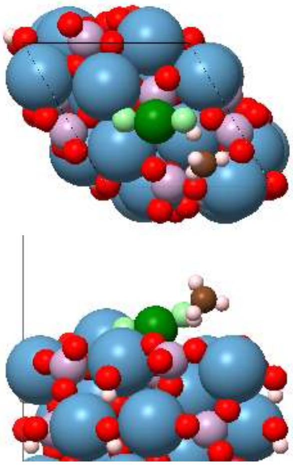	3559.38, 3240.76, 3220.60, 3039.28, 1370.21, 1344.53, 748.00, 723.41, 546.41, 282.32, 175.55, 174.67, 126.87, 108.21, 60.50, 31.96, 21.75, −274.64
$CH_3OH^* \xrightarrow[\text{Fe/HAP}]{TS_1} CH_3{}^*/TM + OH^*$	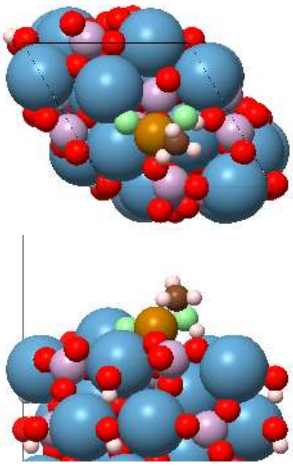	3333.60, 3189.32, 3104.65, 2749.04, 1492.63, 1354.25, 1264.72, 1014.21, 985.12, 870.84, 719.53, 638.42, 323.65, 208.23, 151.37, 105.62, 84.13, −474.08
$CH_3{}^*/TM \xrightarrow[\text{Fe/HAP}]{TS_2} CH_3{}^*/O$	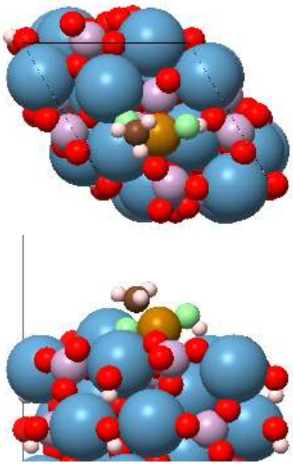	3517.88, 3149.58, 3065.33, 2933.46, 1434.26, 1366.90, 1171.57, 827.65, 812.45, 718.99, 591.18, 503.05, 461.71, 156.15, 133.94, 101.20, 70.95, −325.73

Continued on next page

System	Structure	Vibrational frequencies (cm^{-1})
$CH_3OH^* \xrightarrow[\text{Co/HAP}]{TS_1} CH_3^*/TM + OH^*$	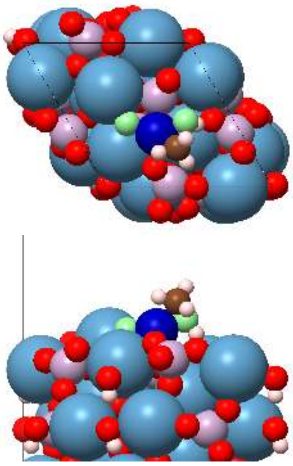	3215.99, 3189.52, 3108.91, 2907.14, 1404.87, 1362.39, 1173.54, 1030.66, 909.36, 804.41, 758.95, 503.18, 353.28, 172.28, 154.14, 111.67, 110.39, −433.24
$CH_3^*/TM \xrightarrow[\text{Co/HAP}]{TS_2} CH_3^*/O$	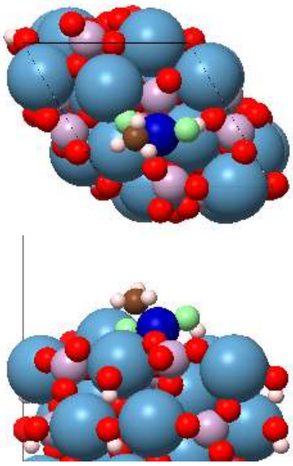	3450.97, 3188.13, 3110.39, 2816.68, 1415.50, 1350.71, 1210.14, 896.79, 844.74, 794.08, 635.00, 572.51, 420.48, 232.70, 172.43, 112.72, 84.68, −287.68
$CH_3OH^* \xrightarrow[\text{Ni/HAP}]{TS_1} CH_3^*/TM + OH^*$	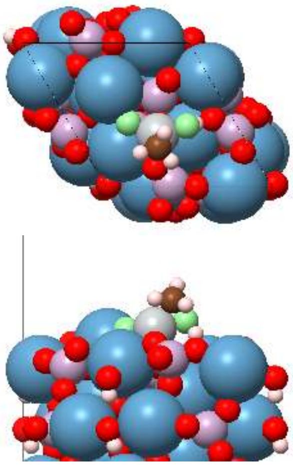	3380.08, 3206.72, 3129.51, 2938.93, 1403.14, 1372.12, 1180.38, 990.85, 888.39, 797.89, 630.50, 477.89, 337.58, 186.21, 168.85, 115.58, 92.76, −420.55

Continued on next page

System	Structure	Vibrational frequencies (cm^{-1})
$CH_3{}^*/TM \xrightarrow[Ni/HAP]{TS_2} CH_3{}^*/O$	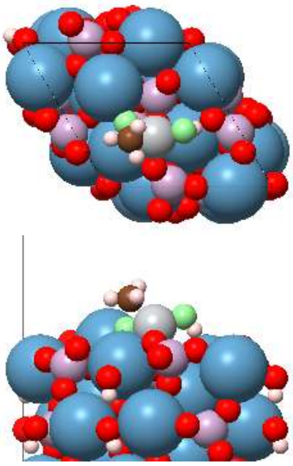	3436.80, 3184.00, 3120.79, 2973.48, 1419.09, 1370.72, 1148.52, 899.91, 832.90, 750.48, 589.38, 566.27, 364.71, 146.37, 121.48, 81.78, 69.40, −285.21
$CH_3OH^* \xrightarrow[Cu/HAP]{TS_1} CH_3{}^*/TM + OH^*$	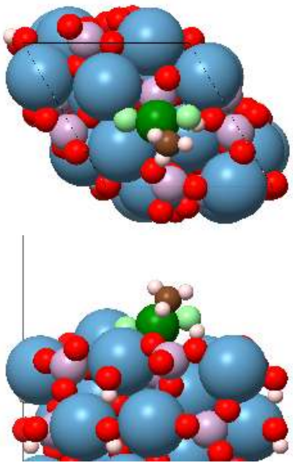	3363.15, 3227.59, 3137.87, 2994.60, 1421.86, 1359.10, 1098.16, 1036.74, 809.50, 734.08, 605.12, 512.68, 384.97, 174.39, 136.66, 113.32, 24.27, −239.92
$CH_3{}^*/TM \xrightarrow[Cu/HAP]{TS_2} CH_3{}^*/O$	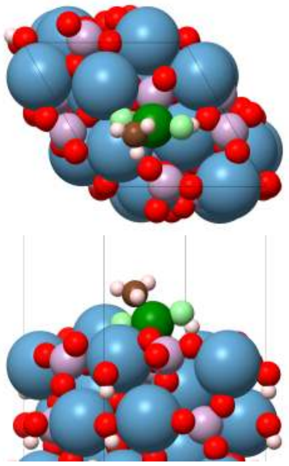	3402.20, 3191.20, 3143.62, 3011.90, 1405.54, 1353.60, 1105.65, 972.53, 752.56, 726.00, 577.14, 526.32, 383.64, 184.28, 110.11, 80.83, 52.68, −266.91

S-9 Additional Computational Parameters and Input Files

The entire set of adsorption structures presented in Section S-4 was optimized based on the slab model for the HAP(0001) 1 × 1 surface unit cell. A cutoff energy of 380 eV was

used, with convergence criteria of 1.0×10^{-4} eV for total energies and 0.050 eV Å^{-1} for geometric optimization. The Brillouin zone integration was performed using only the Γ-point to reduce computational cost in this preliminary part of the study. Subsequently, after identifying the lowest-energy configurations, further structural optimization was performed with stricter convergence criteria of 1.0×10^{-5} eV for total energies and 0.025 eV Å^{-1} for geometric optimization. This optimization used an increased cutoff energy of 489 eV and a **k**-point mesh of $2 \times 2 \times 1$. For the localization of transition states in the reaction path, these computational parameters were applied in conjunction with the climbing image nudged elastic band method (CI-NEB). In the following, we provide two examples of input files (INCAR) used for structural optimization and CI-NEB calculations.

S-9.1 Input File for the Structural Optimization Calculations

```
SYSTEM = chemical_system_name

Electronic relaxation:
ENCUT   = 488.734   ! cutoff energy for plane waves basis set in eV
ALGO    = All       ! electronic minimisation algorithm
NELMIN  = 6         ! minimum number of eletronic selfconsistency (SC) steps
NELM    = 200       ! maximum number of electronic SC steps
NELMDL  = -20       ! number of non-selfconsistency steps
EDIFF   = 1.0E-5    ! global-break condition for the electronic SC-loop
AMIX    = 0.10000
BMIX    = 0.00010
AMIX_MAG= 0.10000
BMIX_MAG= 0.00010
LMAXMIX = 4

Calculation mode:
PREC    = Normal    ! precision-mode
ISPIN   = 2         ! non-spinpolarized calculations
ADDGRID = .TRUE.    ! PAW flag
LASPH   = .TRUE.    ! non-spherical elements in the PAW method
LREAL   = AUTO      ! evaluation of projection operators
IVDW    = 11        ! vdW correction (Grimme DFT-D3)

Ionic relaxation:
NSW     = 100       ! number of ionic steps
EDIFFG  = -0.025    ! stop if all forces are smaller than |EDIFFG|
IBRION  = 2         ! conjugate-gradient algorithm
ISIF    = 0         ! calculate forces and ion relaxation
POTIM   = 0.50

Integration over the Brillouin zone (BZ):
ISMEAR  = 0         ! Gaussian smearing
SIGMA   = 0.01

DOS calculation:
```

```
LORBIT  = 10        ! calculate the DOS without providing the Wigner Seitz radius
NEDOS   = 11        ! number of points to calculate the DOS

OUTCAR size:
NWRITE  = 1         ! determines how much information will be written in OUTCAR
LWAVE   = .FALSE.
LCHARG  = .FALSE.

Key for parallel mode calculation:
NCORE  = 4
LPLANE = .TRUE.

EOF
```

S-9.2 Input File for the CI-NEB Calculations

```
SYSTEM = chemical_system_name

Electronic relaxation:
ENCUT   = 488.734   ! cutoff energy for plane waves basis set in eV
ALGO    = All       ! electronic minimisation algorithm
NELMIN  = 6         ! minimum number of eletronic selfconsistency (SC) steps
NELM    = 200       ! maximum number of electronic SC steps
NELMDL  = -20       ! number of non-selfconsistency steps
EDIFF   = 1.0E-5    ! global-break condition for the electronic SC-loop
AMIX    = 0.10000
BMIX    = 0.00010
AMIX_MAG= 0.10000
BMIX_MAG= 0.00010
LMAXMIX = 4

Calculation mode:
PREC    = Normal    ! precision-mode
ISPIN   = 2         ! non-spinpolarized calculations
ADDGRID = .TRUE.    ! PAW flag
LASPH   = .TRUE.    ! non-spherical elements in the PAW method
LREAL   = AUTO      ! evaluation of projection operators
IVDW    = 11        ! vdW correction (Grimme DFT-D3)

Ionic relaxation:
NSW     = 100       ! number of ionic steps
EDIFFG  = -0.025    ! stop if all forces are smaller than |EDIFFG|
IBRION  = 3         ! must set to disable the built in optimizers
ISIF    = 0         ! must set to disable the built in optimizers
POTIM   = 0

CI-NEB caculation:
LCLIMB  = .TRUE.    ! turn on the climbing image algorithm
ICHAIN  = 0         ! enable NEB calculation
IOPT    = 3         ! "quick-min" optimizer
IMAGES  = 12        ! number of images along the reaction path
SPRING  = -5        ! spring constant

Integration over the Brillouin zone (BZ):
ISMEAR  = 0         ! Gaussian smearing
```

```
SIGMA   = 0.01

DOS calculation:
LORBIT  = 10        ! calculate the DOS without providing the Wigner Seitz radius
NEDOS   = 11        ! number of points to calculate the DOS

OUTCAR size:
NWRITE  = 1         ! determines how much information will be written in OUTCAR
LWAVE   = .FALSE.
LCHARG  = .FALSE.

Key for parallel mode calculation:
NCORE  = 4
LPLANE = .TRUE.

EOF
```

References

1 Kresse, G.; Hafner, J. *Ab initio* Molecular Dynamics for Open-Shell Transition Metals. *Phys. Rev. B* **1993**, *48*, 13115–13118, DOI: 10.1103/physrevb.48.13115.

2 Kresse, G.; Furthmüller, J. Efficient Iterative Schemes For *Ab Initio* Total-Energy Calculations Using a Plane-Wave Basis Set. *Phys. Rev. B* **1996**, *54*, 11169–11186, DOI: 10.1103/physrevb.54.11169.

3 Blöchl, P. E. Projector Augmented-Wave Method. *Phys. Rev. B* **1994**, *50*, 17953–17979, DOI: 10.1103/PhysRevB.50.17953.

4 Kresse, G.; Joubert, D. From Ultrasoft Pseudopotentials to the Projector Augmented-Wave Method. *Phys. Rev. B* **1999**, *59*, 1758–1775, DOI: 10.1103/physrevb.59.1758.

5 Bittencourt, A. F. B.; Mendes, P. C. D.; Valença, G. P.; Da Silva, J. L. F. Acid-Base Properties of Hydroxyapatite(0001) by the Adsorption of Probe Molecules: An *Ab Initio* Investigation. *Phys. Rev. Mater.* **2021**, *5*, 075003, DOI: 10.1103/PhysRevMaterials.5.075003.

6 Brasil, H.; Bittencourt, A. F. B.; Yokoo, K. C. E. S.; Mendes, P. C. D.; Verga, L. G.; Andriani, K. F.; Landers, R.; Da Silva, J. L. F.; Valença, G. P. Synthesis Modification of Hydroxyapatite Surface for Ethanol Conversion: The Role of the Acidic/Basic Sites Ratio. *J. Catal.* **2021**, *404*, 802–813, DOI: 10.1016/j.jcat.2021.08.050.

7 Bittencourt, A. F. B.; Valença, G. P.; Da Silva, J. L. F. Elucidating the Catalytic Valorization of Ethanol over Hydroxyapatite for Sustainable Butanol Production: A First-Principles Mechanistic Study. *J. Phys. Chem. C* **2024**, *128*, 14663–14673, DOI: 10.1021/acs.jpcc.4c03937.
